# East African cichlid lineages (Teleostei: Cichlidae) might be older than their ancient host lakes: new divergence estimates for the east African cichlid radiation

**DOI:** 10.1186/s12862-019-1417-0

**Published:** 2019-04-25

**Authors:** Frederic Dieter Benedikt Schedel, Zuzana Musilova, Ulrich Kurt Schliewen

**Affiliations:** 1 Department of Ichthyology, SNSB - Bavarian State Collection of Zoology, Münchhausenstr. 21, 81247 Munich, Germany; 2 Department of Zoology, Faculty of Science, Charles University, Vinicna 7, CZ-128 44 Prague, Czech Republic

**Keywords:** East African cichlid radiation (EAR), Molecular clock, Lamprologini, Congo River, African Great Lakes, *Tugenchromis*

## Abstract

**Background:**

Cichlids are a prime model system in evolutionary research and several of the most prominent examples of adaptive radiations are found in the East African Lakes Tanganyika, Malawi and Victoria, all part of the East African cichlid radiation (EAR). In the past, great effort has been invested in reconstructing the evolutionary and biogeographic history of cichlids (Teleostei: Cichlidae). In this study, we present new divergence age estimates for the major cichlid lineages with the main focus on the EAR based on a dataset encompassing representative taxa of almost all recognized cichlid tribes and ten mitochondrial protein genes. We have thoroughly re-evaluated both fossil and geological calibration points, and we included the recently described fossil †*Tugenchromis pickfordi* in the cichlid divergence age estimates.

**Results:**

Our results estimate the origin of the EAR to Late Eocene/Early Oligocene (28.71 Ma; 95% HPD: 24.43–33.15 Ma). More importantly divergence ages of the most recent common ancestor (MRCA) of several Tanganyika cichlid tribes were estimated to be substantially older than the oldest estimated maximum age of the Lake Tanganyika: Trematocarini (16.13 Ma, 95% HPD: 11.89–20.46 Ma), Bathybatini (20.62 Ma, 95% HPD: 16.88–25.34 Ma), Lamprologini (15.27 Ma; 95% HPD: 12.23–18.49 Ma). The divergence age of the crown haplochromine H-lineage is estimated to 22.8 Ma (95% HPD: 14.40–26.32 Ma) and of the Lake Malawi radiation to 4.07 Ma (95% HDP: 2.93–5.26 Ma). In addition, we recovered a novel lineage within the Lamprologini tribe encompassing only *Lamprologus* of the lower and central Congo drainage with its divergence estimated to the Late Miocene or early Pliocene. Furthermore we recovered two novel mitochondrial haplotype lineages within the Haplochromini tribe: ‘*Orthochromis*’ *indermauri* and ‘*Haplochormis*’ *vanheusdeni.*

**Conclusions:**

Divergence time estimates of the MRCA of several Tanganyika cichlid tribes predate the age of the extant Lake Tanganyika basin, and hence are in line with the recently formulated “Melting-Pot Tanganyika” hypothesis. The radiation of the ‘Lower Congo *Lamprologus* clade’ might be linked with the Pliocene origin of the modern lower Congo rapids as has been shown for other Lower Congo cichlid assemblages. Finally, the age of origin of the Lake Malawi cichlid flock agrees well with the oldest age estimate for lacustrine conditions in Lake Malawi.

**Electronic supplementary material:**

The online version of this article (10.1186/s12862-019-1417-0) contains supplementary material, which is available to authorized users.

## Background

The exceptional diversity and propensity to generate adaptive radiations have made cichlid fishes one of the most important vertebrate model systems for evolutionary biology research [1–4]. Much effort has been invested in the reconstruction of the evolutionary time scale and biogeographic history of cichlids distributed in the Americas, Africa, the Middle East, Madagascar and the Indian subcontinent [5–9]. The primary focus has been on the biogeographic origin of the cichlids from the so-called East African Radiation (EAR), i.e., the clade that comprises the famous megadiverse radiations of the East African Lakes Tanganyika (LT), Malawi (LM) and Victoria (LV). Nevertheless, there remains debate over the divergence age estimates of their origin, as well as a lack of a precise reconstruction of their paleogeographic environments providing the stage for these spectacular radiations. One of the reasons is that unambiguous and important calibration points for molecular clock estimates, e.g. a consolidated root age of the family Cichlidae or a lack of cichlid fossils within EAR with the phylogenetically clear position.

Two major hypotheses relating to the problem of the cichlid root age have been proposed, i.e., the *Vicariance Hypothesis* and the *Dispersal Hypothesis*. The former places the cichlid origin before the Gondwana fragmentation and is supported by evidence for reciprocally monophyletic cichlid lineages on in Africa (Pseudocrenilabrinae) and the Americas (Cichlinae), a pattern that is more difficult to envisage under the second hypothesis. This postulates a marine dispersal of early cichlids after tectonic separation of South America, Africa and Madagascar and is supported by the fact, that the oldest cichlid fossil, †*Mahengechromis*, is of only Eocene age (approx. 46 Ma, [10]). Both the Gondwana divergence date based on tectonics as well as the cichlid fossil calibrations have been previously used as calibration priors in molecular clock studies on cichlids and yield, not surprisingly, dramatically different divergence time estimates and biogeographic implications, not only for the EAR evolution ([5–8, 11, 12]). For example, the most recent study on this subject based on the yet most comprehensive dataset inferred a mean divergence age of New World and African cichlid lineages of approximately 82 Ma, i.e. soon after the final separation of Africa and South America ([9]), whereas other recent studies infer either substantially younger (approx. 46 Ma; [7]) or substantially older (approx. 147 Ma; [13]) divergence ages for this split. Consequently, different age estimates for EAR-lineages turned out to be highly divergent as well [5, 14]. To further complicate the issue, inferred lake ages of the African great lakes or their lake level histories have frequently been used to calibrate cichlid molecular clocks under the assumption that endemic clades diverged only after lake formation or, following complete lake basin desiccation, after refilling events [14–16]. This approach is problematic for several reasons. Firstly, the geological history of the formation of the East African rift lakes is highly complex and still not fully understood; therefore, the geological age and onset of truly lacustrine conditions of LT continues to be under debate (e.g. [17]). Several EAR molecular clock studies used an age of 9–12 Ma as a calibration point for the formation of the LT lacustrine basin (e.g. [14, 15]). This age was based on extrapolation of recent sedimentation rates in LT under the assumption of roughly uniform sedimentation rates over the past million years. This assumption is most likely too simplistic, because dramatic climate changes as well as the highly dynamic East African rift tectonics and their associated volcanism must have influenced sedimentation rates substantially [17–19]. Indeed, more recent studies based on thermochronology and sedimentology constrain pre-rift formation of the Albertine Rift system to 4–11 Ma, and the onset of true rifting activity at around 5.5 Ma in the norther LT basin; this in turn implies a much younger age for modern LT than 9–12 Ma [20–23]. Secondly, some of recent endemic and sympatric LT cichlid lineages are likely to have evolved independently in the larger proto-LT drainage area, and only later met and possibly hybridized in the extant LT basin [17]. Hence, as the true age of extant lake basin formation of LT remains unknown and as the assumption of all LT cichlid lineages having originated in situ is not unambiguously supported, studies using a presumably precise age 9–12 Ma as calibration prior for the origin of endemic lacustrine LT fish radiations are potentially misleading. In a analogous case, the age of endemic Lake Malawi lacustrine cichlid lineages has previously been constrained in molecular clock analyses [15, 16] to be younger than the postulated complete desiccation of Lake Malawi either at around 1.6–1.0 Ma, or at the post-drought re-establishment of truly lacustrine conditions at 1.0–0.57 Ma [24]. This approach is in conflict with a recent study reporting continuous sedimentation in the geological LM basin over the last 1.3 Ma, i.e. raising doubts about the previously used LM calibration points [25] Therefore, the use of the sedimentology-based lake age estimates as molecular clock calibration points for the origin of cichlid taxa endemic to large and paleogeographically complex rift lakes appears problematic and might result in highly misleading node age estimates.

Nevertheless, relative and absolute divergence time estimates remain essential for the study of the cichlid biogeographic origin and the history of evolutionary processes whose interplay generated the yet unrivaled vertebrate diversity within the dynamic landscape of the East African rift and its lakes (e.g., [17, 26]). Ultimately, the spatiotemporal reconstruction of phylogenetic relationships between riverine and lacustrine East African cichlid lineages may not only inform evolutionary biology but will also help to reconstruct geomorphological landscape evolution in the identification of lake colonization routes and river capture events [27].

Here we present new divergence age estimates based on eighteen different calibration sets, which includes up to seven different calibration points represented by three neotropical cichlid fossils, up to three pseudocrenilabrine cichlid fossils and one geological event; the root was calibrated with three different secondary constraints (see below). In particular, the recently described African cichlid fossil †*Tugenchromis pickfordi* is included [28]. Our primary sequence alignment consists of ten mitochondrial protein coding genes including representatives of almost all recognized cichlid tribes with emphasis on the EAR. The application of eighteen different calibration sets enabled us to compare recent cichlid molecular clock studies with our findings, as well as to infer the impact of †*Tugenchromis pickfordi* as a calibration point. There is a long-standing debate about the applicability of mitochondrial DNA data for molecular clock estimates. For example, Matschiner [29] pointed out that nuclear marker-based studies (e.g. [7, 29]) often yield younger divergence time estimates than mitochondrial marker-based studies (e.g. [5, 6]). Therefore, we complement our mtDNA-based analyses (calibration Sets 1–17) with an independent nuclear DNA-based alignment (calibration Set 18) to infer whether or not an identical calibration strategy would result in similar node age estimates for both data sets. We provide a new relative divergence time frame for the African Pseudocrenilabrinae and especially for the mtDNA lineages belonging to the EAR, which is critical in the context of clarifying the phylogeographic history and origin of the famous adaptive radiations of LT, LM and LV but also of several smaller haplochromine lineages. In this study, we include several riverine lineages for the first time allowing for new insights into the complex evolutionary history of the EAR.

## Methods

### Taxon and nucleotide sampling

Ten mitochondrial protein coding genes were sequenced or obtained from Genbank for 180 cichlid species (Additional file 1: Table S1). The focus of the data set was on taxa representing all major lineages of the East African cichlid Radiation (EAR), but members of all other cichlid subfamilies were included as well: Madagascan and Asian Etroplinae (*N* = 2) and Ptychrominae (*N* = 2), American Cichlinae (*N* = 31) and African Pseudocrenilabrinae (*N* = 145). The latter are represented by almost all major tribes including Tylochromini (N = 1), chromidotilapiines (*N* = 2), hemichromines (N = 2), pelmatochromines (*N* = 1); haplotilapiine lineages (sensu Schliewen & Stiassny, 2003) are represented by the mouthbrooding Oreochromini (*N* = 14), substrate brooding Pelmatotilapiini (*N* = 1) and boreotilapiines (sensu Schwarzer et al. 2009, Dunz et al., 2013) including Coptodonini (*N* = 2) and Gobiocichlini (*N* = 1) and austrotilapiines (*N* = 121). The austrotilapiine lineage is represented by Tilapiini (*N* = 3), Steatocranini (*N* = 3) and EAR lineages. The taxon sampling of the EAR lineages (*N* = 115) comprised members of all formally described Lake Tanganyika tribes (sensu Takahashi [30] and Koblmüller et al. [31]), i.e. Boulengerochromini (*N* = 1), Bathybatini inclunding Hemibatini (*N* = 7), Trematocarini (*N* = 5), Lamprologini (*N* = 16) including nine riverine taxa of the Congo basin sensu stricto and the Lufubu River (a southern affluent to Lake Tanganyika), Eretmodini (*N* = 2), Cyphotilapiini (N = 2), Limnochromini (*N* = 2), Ectodini (*N* = 6), Perissodini (N = 2), Cyprichromini (*N* = 2), Benthochromini (N = 1) and Tropheini (*N* = 9; a subgroup of Haplochromini). Moreover, the dataset contains representatives of several additional riverine taxa representing informally named lineages. Since the placement of many recently discovered riverine Haplochromini in Greenwood’s classification [32] of *Haplochromis* and related taxa is problematic, we accounted for these taxonomic uncertainties by placing species of unsettled generic status in the catch-all genera ‘*Haplochromis*’, ‘*Orthochromis*’ or ‘*Ctenochromis*’; this follows the practice first suggested by Hoogerhound [33] and later adopted by several studies (e.g. [17]). Nomenclature for most of these lineages follows Schwarzer et al. [34] and Weiss et al. [17], i.e. we included Northern-Zambia-*Orthochromis* (4), LML-*Orthochromis* occurring at Luapula-Mweru system and the Lualaba/Congo main stem (*N* = 1), Malagarasi-*Orthochromis* (*N* = 4) as well most rheophilic mtDNA lineages of the Congo basin, i.e. ‘*Orthochromis*’ *indermauri*, ‘*Orthochromis*’ *torrenticola*, ‘*Orthochromis*’ *stormsi*; further included are ‘*Haplochromis*’ *vanheusdeni* (N = 1), *Astatoreochromis straelini* (N = 1), Congo-basin *‘Haplochromis’* (*N* = 3)*, Ctenochromis pectoralis (N = 1)*, *‘Pseudocrenilabrus-*group’ (*N* = 9; including the Northern-Zambia-*Orthochromis*) and serranochromines-mtDNA-lineage (*N* = 11; including the Congo-basin *‘Haplochromis’ and* LML-*Orthochromis*) as well as two undescribed species referred here as “New Kalungwishi Cichlid” and “New Lufubu Cichlid”. We further included representative members of all major lineages of the Lake Malawi species flock (*N* = 22) as well as riverine and ‘modern’ Haplochromini of East Africa (*N* = 10). Selection of representative taxa was optimized to encompass the oldest divergence events of clades within austrotilapiine mitochondrial clades and is based on previous studies (e.g. [35–39]). This approach was chosen to infer the oldest mtDNA divergence age estimates for each of these lineages.

In addition to the mitochondrial data set we generated a second data set based on partial sequences of four nuclear loci, i.e. RAG1, ENC1, RH1 and TMO-4c4. All these sequences were obtained from GenBank with the aim to compile a widely comparable taxon sampling to our mitochondrial data set. Since more than one sequence per locus was available for several species, only the most complete sequence of each locus was chosen, and, where ever possible, sequences of the same species would derive from the same study and individual. Furthermore, to obtain a dataset with few missing data only taxa with two or more loci represented in Genbank were kept (except *Gymnogeophagus balzanii* and *Gymnogeophagus setequedas* for which only one locus was available). In total, the nuclear data set included 117 species representing all cichlid subfamilies and most of the major lineages of the EAR (Additional file 2: Table S3). Nevertheless, sequences of several comparatively recently diverged lineages were not available in Genbank, e.g. Malagarasi-*Orthochromis,* ‘*Haplochromis*’ *vanheusdeni,* the Congo-basin *‘Haplochromis,* LML-*Orthochromis, Astatoreochromis, Ctenochromis pectoralis,* ‘*Haplochromis*’ *vanheusdeni* and ‘*Orthochromis*’ *indermauri*.

### Sampling procedures

Material for this study was obtained from the commercial cichlid fish trade in Germany, private collection of aquarium hobbyists or collected on previous field trips. Individual fish were either caught using various fishing methods (gill net, beach seine net, gill net, hand net) or bought freshly fished from local fishermen. Freshly caught fish were sacrificed by an overdose of approved fish anesthetic (Benzocaine, MS-222). Subsequently, fin clips were fixed in 96% ethanol and entire specimens were fixed in 10% formalin, as explained in [40]. We followed all applicable international and national guidelines of animal use and ethical standards for the collection of samples.

### Molecular methods

Total genomic DNA was extracted by using the DNeasy Blood & Tissue Kit (Qiagen) following the manufacturer’s protocol and the DNA concentration was standardized to 25 ng/μl. We either amplified the whole mitochondrial genome or three large fractions using the following three primer pairs: Primer pair A (L2508KAW: 5’-CTC GGC AAA CAT AAG CCT CGC CTG TTT ACC AAA AAC-3’; [41]; and ZM7350R: 5’-TTA AGG CGT GGT CGT GGA AGT GAA GAA G-3`), Primer pair B (ZM7300F:5`-GCA CAT CCC TCC CAA CTA GGW TTT CAA GAT GC-3’ and ZM12300R: 5’-TTG CAC CAA GAG TTT TTG GTT CCT AAG ACC-3’) and Primer pair C (ZM12200F: 5’-CTA AAG ACA GAG GTT AAA ACC CCC TTA TYC-3’ and ZM2100R: 5’-GAC AAG TGA TTG CGC TAC CTT TGC ACG GTC-3; all ZM primers taken from [9]; the number in the primer names refers to an approximate position within the mitogenome starting by the tRNA-Phe). The amplified fragments overlapped and enabled the assembly of contiguous mitochondrial genome fragments across primer sites. Long-range PCR were conducted using the TaKaRa LA Taq DNA polymerase kit (TaKaRa) with the following thermal profiles: initial denaturation at 98 °C (60 s), followed by 35 cycles of denaturation 98 °C (10 s), annealing at60°C (Primer pair A), 62 °C (Primer pair B) or 60 °C (Primer pair C) for 60s, elongation at 68 °C (15 min), and a last extension step at 72 °C (10 min). Amplification products were purified using the QIAquick Gel Extraction Kit (Qiagen) following the manufacturer’s protocol. DNA concentration of purified amplification products were adjusted to 0.21 ng/μl and fragments of each species were pooled equimolarly. The Nextera XT DNA Sample Preparation Kit (Illumina) was used for library preparation following the manufacturer’s protocol until the normalization step. Library pooling and sequencing was conducted at the Sequencing Service of the Ludwig Maximilian University of Munich on an Illumina MiSeq platform. Alternatively, several samples were sequenced on the Ion Torrent PGM platform following the library preparation using the Ion Xpress™ Plus Fragment Library Kit and the template preparation on the Ion OneTouch™ 2 System (following OT2 protocol). Adaptor trimming, quality control and assembly of the sequencing reads were done by using the CLC Genomics Workbench (Qiagen). Annotation of the assembled sequences (mean coverage: 6820; mean sequence length: 9923 bp) was performed in Geneious v.7.05 [42] using the complete mitochondrial genome of *Oreochromis niloticus* as a reference genome (GenBank accession number: GU370126; [43]). Sequence data were deposited in Genbank under the accession numbers (MK144668 – MK144786 and MK170260 – MK170265, Additional file 1: Table S1). To complement our data set we included published mitochondrial genomes from previous studies that were deposited in GenBank (Additional file 1: Table S1).

### Phylogenetic analysis, divergence time estimate and fossil calibration

We extracted protein coding sequence information of ten mitochondrial protein-coding genes (ND1, ND2, COX1, COX2, ATP8, ATP6, COX3, ND3, ND4L, ND4) for all taxa from of our data set. If sequences of a particular gene were missing (e.g. due to poor quality of sequence) a multi-N string was inserted into the alignment in the respective positions (Additional file 1: Table S1). For *Lamprologus tigripictilis* three genes were missing, therefore we used the ND2 sequence of another specimen of the sampled at the same river location (Genbank accession number: JX157061) to complement sequence information for this species. Sequences were aligned for each gene separately using the Geneious alignment tool with default settings and then checked by eye. Single gene alignments were concatenated in Geneious, resulting in a total alignment of 7893 bp with 4529 variable sites and relative base frequencies (excluding gaps and ambiguous sites) of A = 0.25, T = 0.28, C = 0.32 and G = 0.15. Each codon position was tested for saturation by calculating the number of transitions and transversions for all taxon pairs for each codon position separately in PAUP v. 4.0 [44] and plotting them against each other.

The complementary four nuclear loci alignment with sequences from Genbank (RAG1, ENC1, RH1 and TMO-4c4) comprised 117 taxa. Missingness was as follows: 16 species had no RAG1 sequence, 8 had no ENC1, 40 had no RH1 and 41 had no TMO-4c4, and missing data were replaced by Ns. Genbank sequences were individually aligned using the Geneious alignment tool with default settings and subsequently checked by eye and trimmed to equal length. All single locus alignments were concatenated in Geneious resulting in a total alignment of 3483 bp and 35.8% missing data. Relative base frequencies (excluding gaps and ambiguous sites) of this alignment are A = 0.24, T = 0.25, C = 0.24 and G = 0.27.

Selection of the best-fitting substitution model (GTR + I + G) for each gene was conducted using the program jModeltest [45] based on Akaike information criterion (AIC). Maximum likelihood (ML) inference of phylogenetic relationships was conducted with RAxML v8.2.6 [46] on the CIPRES Science Gateway [47]. For this step, the data set was further partitioned into first, second and third codon positions and the two Etroplinae taxa *Etroplus maculatus* and *Paretroplus maculatus* were defined as outgroup, based on consilient evidence from previous phylogenetic studies [7, 8]. Bootstrap replications were automatically halted by RAxML (using the majority rule criterion) after 108 replications followed by ML search. Relative divergence times of clades were estimated using the Bayesian software BEAST v2.3.2 [48] under a relaxed lognormal clock model with a birth-death speciation model on the CIPRES Science Gateway. Again, the data set was partitioned in first, second and third codon position. Moreover, for the BEAST analysis we defined five clades as monophyletic based on the results of the Maximum Likelihood analysis (see above): Clade 1 (Ptychochrominae + Pseudocrenilabrinae + Cichlinae), Clade 2 (Pseudocrenilabrinae + Cichlinae), Clade 3 (Pseudocrenilabrinae), Clade 4 (Cichlinae) and Clade 5 (containing: austrotilapiines, Pelmatolapiini, Oreochromini). These clades were supported by high bootstrap values (except Clade 2 and Clade 5) in our analysis and were concordant by previous studies (e.g. [5, 7, 8]).

Calibration points were chosen conservatively based on a critical evaluation of all previously used calibration points in cichlid phylogenetic studies. Up to six fossils (three Neotropical cichlid fossils and three fossils belonging to the Pseudocrenilabrinae) and one geological event (geological age of the crater lake Barombi Mbo maar) were finally selected. Justifications for their inclusion is detailed below; for reasons why previously used cichlid fossils and geological calibration points were excluded, see the Additional file 3. Ninety five percent quantiles of prior-probability-densities width for fossil calibration points laid between 29.2 and 39.1 Ma, which roughly matches the recommendation by [9]). Generally, only fossils with well evaluated evidence for their phylogenetic position and with equally well corroborated ages were included.

The three neotropical cichlid fossil are: †*Plesioheros chaulidus, †Gymnogeophagus eocenicus* and †*Tremembichthys* (e.g. *†T. paulensis and †T. garciae)*.

†*P. chaulidus* and †*G. eocenicus* were described from lacustrine “Faja Verde” deposits of the uppermost section of the Lower Lumbrera formation in Northwestern Argentina [49, 50]. The exact age of “Faja Verde” deposits remains under debate, but it is possible to constrain the youngest possible age of the whole Lumbrera formation to 39.9 Ma based on U/Pb dating of its uppermost layer [51]; and it is possible to constrain the cichlid bearing layer to a maximum age of 45.4–38.0 Ma based on accompanying mammal fossils, whose association suggests an Casamayoran-Vacan age (for a more detailed discussion of the age of Lumbrera formation see [9, 52], who used the same calibration). The phylogenetic placement of †*Plesioheros chaulidus* within the Cichlinae tribe Heroini is well supported by several morphological synapomorphies, but a refined placement of †*Plesioheros* is hampered by the presence of lingual cusps on the teeth in the fossil, which are not present in the two heroine genera *Hypselecara* and *Hoplarchus* [53]. Since phylogenetic analyses of Heroini intrarelationships based on morphological [53] and molecular datasets (e.g [52, 54]) are partially incongruent, and since our Heroini taxon sampling is limited to a few key taxa, we conservatively place †*Plesioheros* at the node uniting only Heroini with lingual cusps being present, i.e. after the divergence of *Hypselecara.* The phylogenetic placement of *†Gymnogeophagus eocenicus* in the extant genus *Gymnogeophagus* is well supported based on two unambigous apomorphies [50]. We conservatively place the calibration point at a node uniting our single *Gymnogeophagus* species (*G. balzanii*) with two other geophagine taxa (*Mikrogeophagus ramirezi*, ‘*Geophagus*’ *brasiliensis*).

*†Tremembichthys* has been recorded from the Entre-Córregos Formation (Aiuruoca Tertiary Basin) and from the Tremembé formation (Taubaté Basin) in Brazil [55] The Entre-Córregos Formation was suggested to be of Eocene-Oligocene age based on palynological evidence [56, 57], whereas lacustrine shales of Tremembé formation are dated to Oligocene-Miocene based on geological and paleontological studies [58, 59]. Phylogenetic analysis based on the character matrix of Kullander [60] placed †*Tremembichthys* within Cichlasomatini, a tribe which is supported by several morphological apomorphies. Of those, however, only the square shaped lachrymal is preserved in †*Tremembichthys* [55]. We accept the placement of †*Tremembichthys* within Cichlasomatini for most of our calibrations, and following [52] we apply a conservative time range of 55.8–23.03 Ma for *†Tremembichthys* as no precise age estimate is available for the Entre-Córregos formation. Nevertheless, it is worth mentioning that *†Tremembichthys* has three pterygiophores articulated with the first haemal arch [55], a condition unknown from any extant Cichlasomatini member. Generally, cichlasomatines have one to two pterygiophores articulated to the first haemal spine whereas some Heroini lineages have three or even more [60]. Therefore, we calibrated one analysis with *†Tremembichthys* at the base of Heroini to evaluate the impact of the alternative plausible placement of *†Tremembichthys*. Phylogenetic placement of all neotropical cichlid fossils was based on Kullander [60] or on López-Fernandez et al. [61]. However, recent molecular studies ([54, 62]) might differ slightly from these phylogenetic hypotheses.

The three included Pseudocrenilabrinae cichlid fossils are: †*Mahengechromis (*e.g. †*Mahengechromis plethos,* †*Mahengechromis rotundus), †Oreochromis lorenzoi* and †*Tugenchromis pickfordi*.

†*Mahengechromis* represents the oldest known cichlid fossil and was discovered in the ancient crater lake Mahenge which is part of the Singida kimberlite field on the Singida Plateau in Tanzania [10, 63]. The age of the Mahenge maar is estimated to 45.83 ± 0.17 Ma based on U/Pb isotope dating, and a maar lake mostly likely persisted for only 0.2–1.0 Ma [64]. The presence of a single supraneural bone places †*Mahengechromis* in a lineage encompassing all Pseudocrenilabrinae except for *Heterochromis*, *Tylochromis* and *Etia* which have two supraneural bones. The phylogenetic position of †*Mahengechromis* has already been discussed in several studies and different positions have been suggested depending on which data sets and characters were used. It was either placed within the EAR, as a basal offshoot within Pseudocrenilabrinae or as a sister group to *Hemichromis* [10, 63, 65]. A sister-group relationship of †*Mahengechromis* and *Hemichromis* was inferred to be most parsimonious based on an osteological character matrix including representatives of all cichlid subfamilies with a focus on the Pseudocrenilabrinae lineages (but missing several important lineages, e.g., pelmatochromines, pelmatolapiines tilapiines, steatocranines), which was mapped on a composite tree with predefined character evolution based on the knowledge of the time. However, when solely based on osteological characters, the relationship between †*Mahengechromis* and *Hemichromis* was not supported [65]. As additional support for a relationship of †*Mahengechromis* and *Hemichromis* Murray [65] stated that both exhibit a low number of total vertebrae (fewer than 26), however this is also the case in other African cichlid genera of the tribes, e.g., Etiini, chromidotilapiines and pelmatochromines [66, 67]. Therefore, we consider the exact phylogenetic placement of †*Mahengechromis* as unresolved, except that it represents an early branching member of Pseudocrenilabrinae. Therefore, we use the fossil age to restrict the maximum ages of the calibration points of †*Oreochromis lorenzoi* and †*Tugenchromis pickfordi* as these taxa undoubtedly represent more derived lineages within Pseudocrenilabrinae (see below).

†*Oreochromis lorenzoi* was described from the Gessoso-Solfifera Formation (Messinian) in Italy [68]. The Messinian age is dated from 7.24–5.33 Ma based on astronomical chronology and ^40^Ar/^39^Ar dating while fossil bearing euxinic shale interstrata of lower evaporite cycles of the Gessoso-Solfifera formation are dated by magnetostratigraphy to 5.96 ± 0.2 Ma [69–71]. No comprehensive phylogenetic analysis is available for †*O. lorenzoi* but its current placement in the tribus Oreochromini is convincingly supported by characters characterizing *Sarotherodon* and *Oreochromis* [68]. Unfortunately, diagnostic characters of several oreochromine genera are often not well preserved in fossils, and moreover, [68] had not compared the fossil with additional genera placed today in Oreochromini, e.g. *Tristramella* and *Danakilia*, rendering the placement of †*O. lorenzoi* to some extent ambiguous [35, 72]. For a conservative approach we therefore decided to use †*O. lorenzoi* as calibration point for the crown age of Oreochromini and not for the genus *Oreochromis*, i.e. with a time range of 5.98–46 Ma based on the age lower of lower evaporite cycles of the Gessoso-Solfifera formation and the maximum age of †*Mahengechromis.*

†*Tugenchromis pickfordi* was recently described from the Waril site of the Ngorora fish Lagerstätte in the Central Kenya Rift Valley [28]. Based on a particular horse (Equidae) tooth fragment of the paleosol above the lacustrine sediments and lithostratigraphy, the Ngorora fish Lagerstätte was assigned to the upper Miocene 9–10 Ma, [73–75]. †*T. pickfordi* can be safely assigned to the family Cichlidae based on several osteological and squamation patterns [28]. Within the Pseudocrenilabrinae it is suggested to be an extinct lineage within the ‘most ancient Tanganyika tribes’ (sensu [17]) based on the character state “lacrimal which bears six lateral line foramina”; this state is present only in six Lake Tanganyika tribes Bathybatini, Perissodini, Limnochromini, Ectodini, Lamprologini and Eretmodini. It most likely represents a stem lineage of the ‘ancient Tanganyika mouth-brooders’ (sensu [17]) as it shares a mosaic-like character set of a tripartite lateral line (present only in two genera of Ectodini from the Lake Tanganyika *Xenotilapia* and *Grammatotria*), a lacrimal with six lateral line foramina, and the shape of the trapezeoid lacrimal and arrangement of tubules resembling strongly those of Limnochromini. Further, its meristics are similar Ectodini and Limnochromini. We therefore accept †*Tugenchromis pickfordi* as a potential precursor lineage of the ‘ancient Tanganyika mouth-brooders’ (sensu Weiss et al. [17]) as this appears to be the most probable phylogenetic position of †*T. pickfordi;* and, alternatively, we use it as a calibration point encompassing the Lake Tanganyika C-lineage (sensu Clabaut et al. [76]), which includes not only the ‘ancient Tanganyika mouth-brooders’, but also the ‘Malagarasi-*Orthochromis’* and Haplochromini (alternative calibration: E1). Nevertheless, we applied two additional alternative calibrations to account for remaining uncertainties of the phylogenetic placement of this fossil. The first included in the C-lineage but also Eretmodini (= H-lineage sensu Nishida [77]; alternative calibration: E2) as Eretmodini exhibit six lateral line foramina as †*Tugenchromis*. The second alternative position of †*Tugenchromis* is at the EAR-bases (alternative calibration: E3), thus accounting for the vague possibility that †*Tugenchromis pickfordi* might be an extinct lineage within the ‘most ancient Tanganyika tribes’, because of its plesiomorphic cycloid flank scales.

We further used one geological event for calibration, i. e. the geological origin of the Cameroonian crater lake Barombi Mbo. The lake harbors an endemic monophyletic radiation of eleven species which must have radiated in situ, and whose riverine founder species, *Sarotherodon galilaeus* is still extant [78, 79]. Based on K/Ar dating the Barombi Mbo maar was active around 1.05 ± 0.7 Ma [80], suggesting a slightly younger age as the maximum age for the onset of the divergence of the cichlid radiation in the lake. In contrast to the complex tectonic history of the East African Great Lakes the volcanic history of the Barombi Mbo maar is far better understood. We therefore decided to include the formation of Barombi Mbo as a maximum age constraint for the MRCA of the strictly endemic Lake Barombi Mbo species flock.

As root calibrations we applied three alternative age-range priors and associated probabilities. One time-range (R1) was set very conservatively by allowing the age prior to range between 46 and 174.78 Ma, either with a lognormal prior (R1a) and or with a uniform probability (R1b). This range covers all possible probabilities for the first emergence of cichlids: the younger bound is based on the age of the oldest known cichlid fossil (46 Ma, †*Mahengechromis*) and the older bound on the oldest maximum age estimate for the family Cichlidae based on independent cichlid molecular clock results (95% HPD: 128.2–174.78 Ma; [13]. The second time-range (R2) is taken from study of Matschiner et al. [9], which is so far the most comprehensively evaluated age estimate for Cichlidae. Their estimate (95% HPD: 82.17–98.91 Ma) is based on a sequence dataset encompassing over 1000 teleost species, 40 mitochondrial and nuclear loci and a calibration with 147 teleost fossils, as well as a critical re-evaluation of previous publications.

To evaluate the effects of inclusion and alternative placement of calibration points, and moreover the impact of different prior distributions (lognormal vs. uniform) for the important root calibration divergence time estimates, we conducted seventeen different BEAST runs based on the mitochondrial dataset and with the following settings. Node calibrations were set to log-normal distributions except for the root calibration (R1b) which in one run was set to a uniform distribution (for more calibration prior details see Table 1): calibration Set 1, Set 2, Set 5, Set 7 and Set 9 were root calibrated using the conservative calibration R1a (prior range of 46–174.78 Ma), while Set 3, Set 4, Set 6, Set 8, Set 10, Set 12, Set 13, Set 14 and Set 15 were calibrated with the root calibration R2 (prior range of 82.17–98.91 Ma). Set 1 and Set 3 were calibrated with †*Tugenchromis* placed on the node of the MCRA of the C-lineage and Eretmodini while Set 2 and Set 4 excluded the Eretmodini in the placement. Set 5 and Set 6 did not include †*Tugenchromis* as a calibration point. Set 9 and Set 10 were calibrated with †*Tugenchromis*, but this time at the base of the EAR*.* †*Oreochromis lorenzoi* was excluded as calibration point from Set 7 and Set 8. Sets 13, 14 and 15 were calibrated as Set 4 except that *†Tremembichthys* was excluded from Set 13 as calibration point, *†Gymnogeophagus eocenicus* as a calibration point from Set 14 and the age of the Barombi Mbo maar as calibration point from Set 15. Set 17 was calibrated as Set 4 except for *†Tremembichthys,* which was placed as a calibration point for the Heroini rather than Cichlasomatini. The calibration of Set 11 was identical to the calibration of Set 2 with the only exception being that the root was calibrated with a uniform distribution (R1b). Set 16 was calibrated as Set 4 but without root calibration. Several studies demonstrated that saturation can lead to the effect of compressing basal branches resulting in overestimated divergence dates of shallow nodes [81–83]. For the evaluation of this effect we designed an additional Set 12 identical to Set 4 but with the third codon position removed of the alignment. Finally, we calibrated the comparative nuclear dataset applying identical settings as the calibration Set 4 to investigate whether mitochondrial and nuclear DNA data calibrated and analysed with identical priors would yield comparable node age estimates. Although the taxon sampling of the comparative nuclear dataset is slightly reduced and not fully identical as comparted to the mitochondrial dataset it covered all major cichlid lineages, hereby enabling a meaningful comparison at least for some divergence time estimates of several key nodes.

**Table 1:** Overview of calibration prior details

Cichlid fossils and geological events used as calibration points:				Parameter settings (Beast):			
Calibration point	Fossil/event	Estimated age	Calibrated clade	Offset	Standard deviation	Mean	Distribution
A1	†*Tremembichthys*	55.8–23.03 Ma (Tremembé formation)	Cichlasomatini	23.03	0.67	2.39	Log normal
A2	†*Tremembichthys*	55.8–23.03 Ma (Tremembé formation)	Heroini	23.03	0.67	2.39	Log normal
B	†*Gymnogeophagus eocenicus*	45.4–39.9 Ma (Lumbrera formation)	*Mikrogeophagus ramirezi*, *Gymnogeophagus balzanii*, ‘*Geophagus*’ *barsiliensis*	39.9	0.8	2.4	Log normal
C	†*Plesioheros chaulidus*	45.4–39.9 Ma (Lumbrera formation)	Heroini (except: of Pterophyllum and Hypselecara)	39.9	0.8	2.4	Log normal
D	†*Oreochromis lorenzoi*	7.24–5.33 Ma (Gessoso-Solfifera formation)	Oreochromini	5.98	1.148	1.8	Log normal
E1	†*Tugenchromis pickfordi*	9–10 Ma (Ngorora Formation)	C-lineage (sensu Clabaut et al., 2005): ‘ancient Tanganyika mouth-brooders’, ‘Malagarasi-*Orthochromis’*, *‘Ctenochromis’ pectoralis* and Haplochromini	9	0.98	2	Log normal
E2	†*Tugenchromis pickfordi*	9–10 Ma (Ngorora Formation)	H-lineage (sensu Nishida, 1991): ‘ancient Tanganyika mouth-brooders’, ‘Malagarasi-*Orthochromis’*, *‘Ctenochromis’ pectoralis,* Haplochromini and Eretmodini	9	0.98	2	Log normal
E3	†*Tugenchromis pickfordi*	9–10 Ma (Ngorora Formation)	East African Radiation (EAR)	9	0.98	2	Log normal
F	Onset Lake Barombi Mbo	1.12–0.98 Ma	Barombi Mbo species flock	0.0	0.07	0.98 (real space)	Log normal
–	†*Mahengeochromis*	45.83 ± 0.17 (Singida kimberlite field)	–	–	–	–	–
Root calibration							
R1a	Time range: 46–174.78 Ma	Based on: Age of †Mahengeochromis & the oldest maximum age estimate for the family Cichlidae (López-Fernández et al. 2013)		46	0.44	3.99	Log normal
R1b	46–174.78 Ma	as for R1a		0	Lower Bound: 46	Upper bound: 174.78	uniform
R2	82.2–98.9 Ma	Estimated divergence age for the family Cichlidae by Matschiner et al. (2016)		82.17	0.455	2.07	Log normal
Combination of calibration points of the different calibration sets:							
	Included calibration points		Included calibration points:		Included calibration points:		
Set 1	A1, B, C, D, E2, F, R1a	Set 7	A1, B, C, E1, F, R1a	Set 13	B, C, D, E1, F, R2		
Set 2	A1, B, C, D, E1, F, R1a	Set 8	A1, B, C, E1, F, R2	Set 14	A1, C, D, E1, F, R2		
Set 3	A1, B, C, D, E2, F, R2	Set 9	A1, B, C, D, E3, F, R1a	Set 15	A1, B, C, D, E1, R2		
Set 4	A1, B, C, D, E1, F, R2	Set 10	A1, B, C, D, E3, F, R2	Set 16	A1, B, C, D, E1, F		
Set 5	A1, B, C, D, F, R1a	Set 11	A1, B, C, D, E1, F, R1b	Set 17	A2, B, C, D, E1, F, R2		
Set 6	A1, B, C, D, F, R2	Set 12 (third codon position stripped)	A1, B, C, D, E1, F, R2	Set 18 (Nuclear data)	A, B, C, D, E1, F, R2		

Fossil taxa are indicated by †

Each BEAST run was performed three times independently (180 million generations per run) and sampling of parameters and trees was done every 15,000 generation. The three independent runs (for each alternative BEAST run configuration) were combined using LogCombiner after accounting for a burn-in of 15%. We used Tracer v1.6 [84] for inspection of effective sample size (ESS) of all parameters of the different BEAST runs. All EES had acceptable values (> 200) and appeared to converge to stationary distributions, indicating an acceptable sample size for the posterior distribution of parameters of individual analyses. Maximum clade credibility trees (posterior probability limit: 0.5, mean heights) were retrieved from the posterior tree distribution.

## Results

**Fig. 1:**
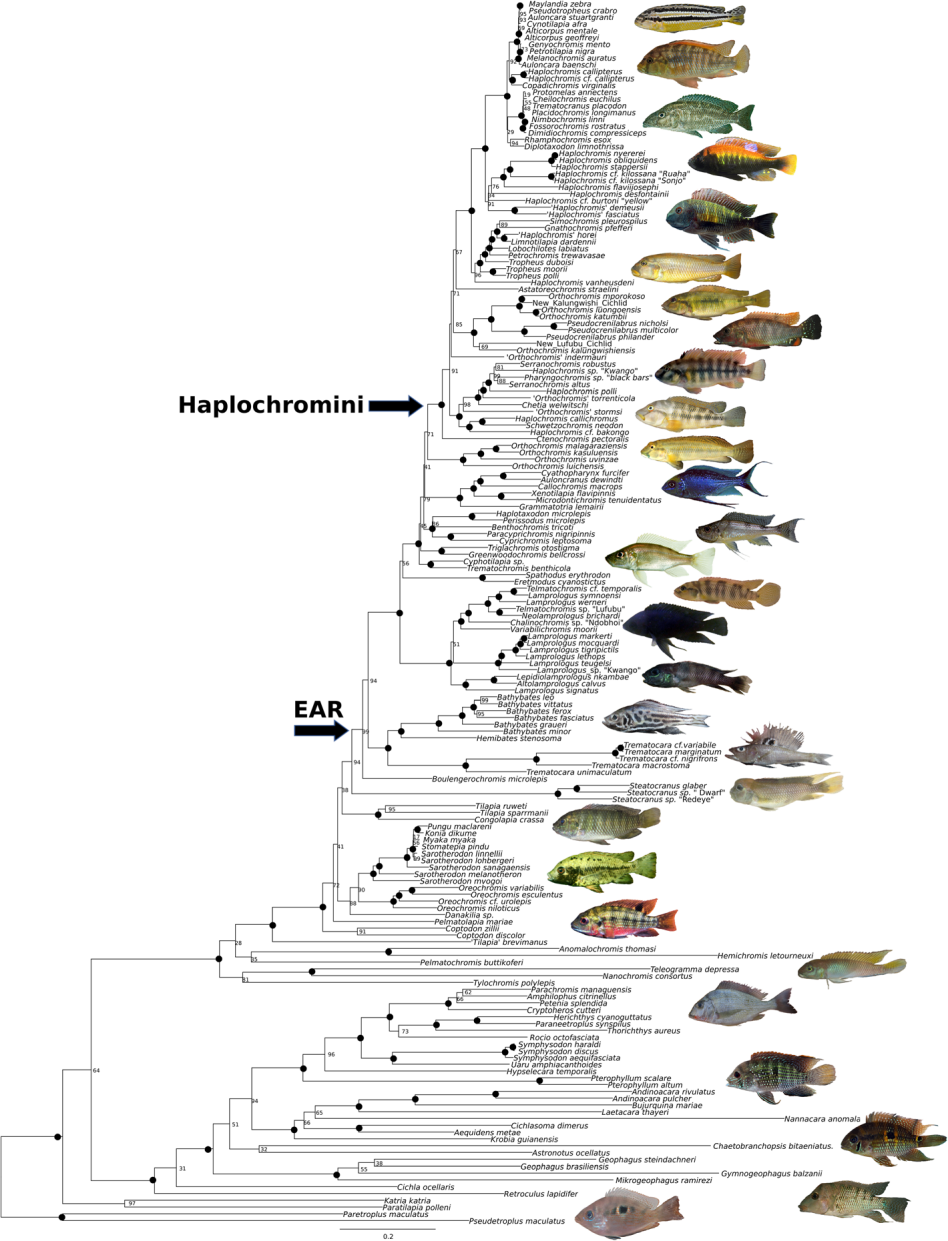
ML-phylogeny (RAxML) based on ten protein coding mitochondrial genes (ND1, ND2, COX1, COX2, ATP8, ATP6, COX3, ND3, ND4L, ND4) of 180 cichlid taxa representing all cichlid subfamilies. Focus of the taxon sampling was put on members of the East African cichlid Radiation represented by 115 taxa. Numbers at nodes refer to bootstrap-values while black dots represent bootstrap support of 100. Specimens depicted from top to bottom (photographersin brackets): *M. auratus* (E. Schraml), *H. callipterus (U.K. Schliewen), N. linni* (E. Schraml)*, H. nyererei* (E. Schraml)*, T. moorii* (Z. Musilová), *H. vanheusdeni* (J. Geck), *O. luongoensis* (F.D.B. Schedel), New Lufubu Cichlid (F.D.B. Schedel), ‘*O.*’ *indermauri* (F.D.B. Schedel), ‘*O.*’ *stormsi* (J. Geck), *O. uvinzae* (J. Geck), *C. furcifer* (Z. Musilová), *H. microlepis* (Z. Musilová), *G. bellcrossi* (E. Schraml), *L. symoensi* (E. Vreven), *V. moorii* (F.D.B. Schedel), *L. teugelsi* (F.D.B. Schedel), *H. stenosoma* (F.D.B. Schedel), *T. macrostoma* (F.D.B. Schedel), *S. glaber* (F.D.B. Schedel), *T. ruweti* (F.D.B. Schedel), *P. maclareni* (J. Geck), *C. zillii* (J. Geck), *N. consortus* (F.D.B. Schedel), *T. polylepis* (F.D.B. Schedel), *A. pulcher* (Z. Musilová), *N. anomala* (Z. Musilová), *G. steindachneri* (Z. Musilová), *P. maculatus* (F.D.B. Schedel)

**Fig. 2:**
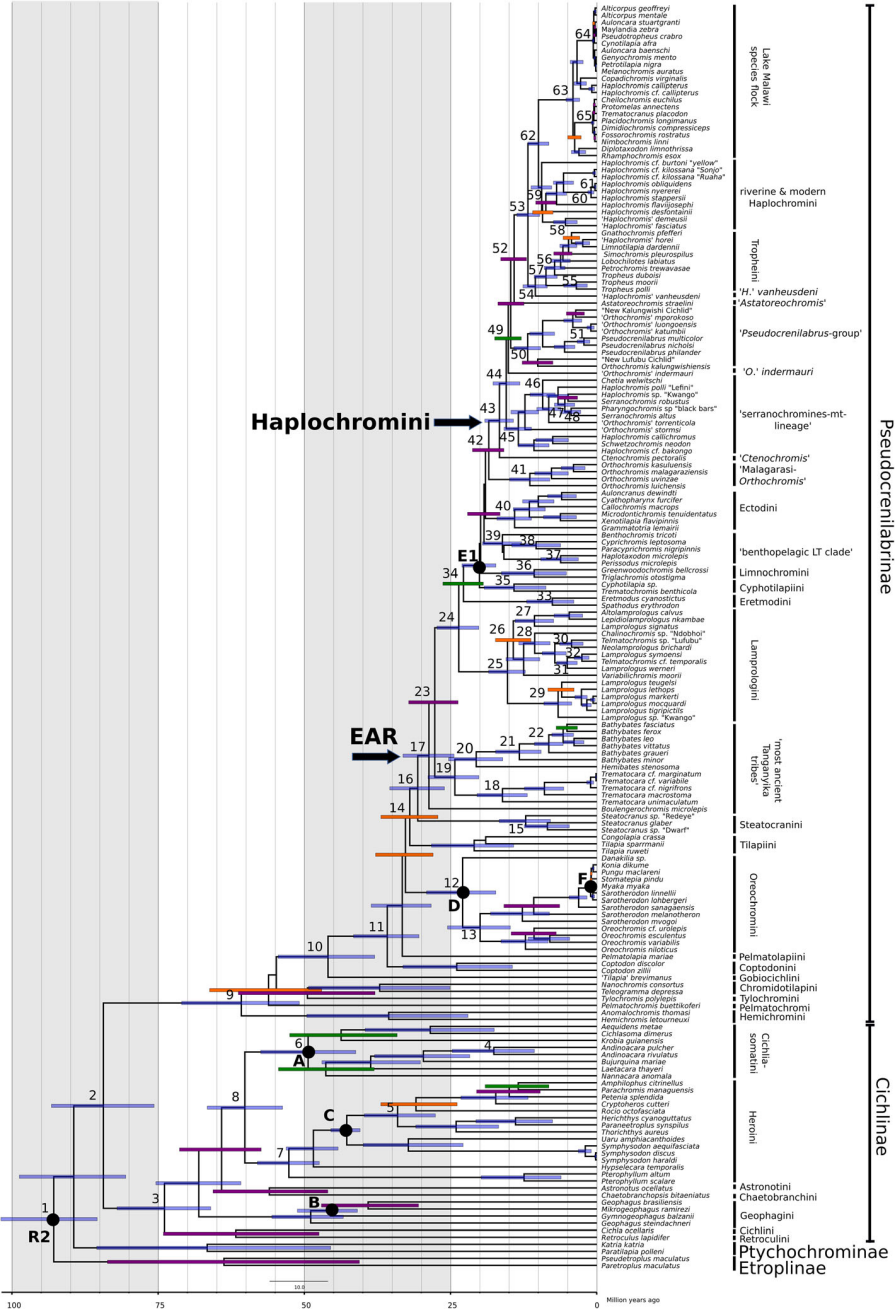
Time-calibrated phylogeny (BEAST, relaxed normal molecular clock) of 180 cichlid taxa based on ten protein coding mitochondrial genes and on the calibration Set 4 (see Table 1). Time constrained nodes (black circles) were calibrated using fossils, i.e. A: †*Tremembichthys,* B: *†Gymnogeophagus eocenicus*, C: *†Plesioheros chaulidus*, D: *†Oreochromis lorenzoi,* E: *†Tugenchromis pickfordi*), one geological event (F: age of Lake Barombi Mbo maare) or as in the case of the root using secondary constraint (divergence time estimate for the age of the MRCA of cichlids taken from Matschiner et al. [9]; 82.17–98.91 Ma). Node bars indicate 95% HPD intervals of divergence events and are coloured according to their Bayesian Posterior Probability (blue: BPP 1.0; violet: BPP 0.99–0.95; green: BPP 0.94–0.8; orange: BPP 0.79–0.5, node bars with BPP < 0.5 are not depicted). Numbers next to the nodes correspond to the numbers in the Additional file 4: Table S2

The alternatively calibrated BEAST runs (Calibration Set 1–11 and Sets 13–17) yielded maximum-clade credibility (MCC) trees which were largely identical to the topology of the ML tree. The few inconsistencies include (a) the position of ‘Lower Congo *Lamprologus* clade’, which is placed as a sister group to all remaining Lamprologini in the Bayesian MCC trees but as a sister group to the ‘non-ossified Lamprologini’ in the ML tree; and (b) the position of Cyphotilapiini which are either a sister group to Limnochromini, or in the ML tree, or a sister group to the clade encompassing Limnochromini and all remaining members of the EAR (see Fig. 1 and Fig. 2). The topology of the maximum-clade credibility (MCC) tree based on the BEAST runs of calibration Set 12 (third codon positions removed) is compatible with those of the ML tree and the MCC trees of the other calibrations sets but show several inconsistencies within the Pseudocrenilabrinae. For example, the Steatocranini are placed as the sistergroup to the EAR in the ML tree and other MCC trees (Sets 1–11 and Sets 13–17) but they are placed as the sister group to a clade comprising Oreochromini, Pelmatolapiini and Tilapiini (*T. ruweti and T. sparrmanii*) in the MMC tree of Set 12. Both *T. ruweti, T. sparrmanii* and *C. crassa* form the sister group to a clade consisting off the EAR and Steatocranini in the ML and all other MCC trees (Set 1–11 and Sets 13–17). The Malagarasi-*Orthochromis* are placed as sistergroup to the Haplochromini in the MCC trees (Set 1–11 and Sets 13–17) and the ML tree but are sistergroup to a clade consisting of Perissodini, Cyprichromini, Benthochromini and Limnochromini in the MMC tree of Set 12. Moreover, the placement of *H. vanheusdeni* and ‘*Orthochromis*’ *indermauri* differed from the ML tree and the other MCC trees (Set- 1 – 11 and Sets 13–17). However, all of these alternative placements in the MMC tree (Set 12) are only weakly supported.

The topology of the MCC tree based on the nuclear dataset resembled those of the mitochondrial dataset to some extent except for several topological differences within the Pseudocrenilabrinae and Cichlinae. Within the Cichlinae, for example, *Astrontus ocellatus* and *Chaetobranchiopsis orbicularis* were resolved as sister taxa to Geophagini instead of forming the sister group to Cichlasomatini and Heroini. There were comparatively minor topological differences of the taxa within Cichlasomatini and Heroini, e.g. the placement of *Krobia* within the Cichlasomatini, and the placement of *Rocio*, *Uaru* and *Symphysodon* within Heroini. Major topological differences within Pseudocrenilabrinae were: ‘*Tilapia*’ *brevimanus* formed a clade together with *Pelmatolapia mariae* which was resolved as a sister clade to the EAR; Steatocranini and Tilapiini were resolved as sister taxa; and within the EAR differences arose for the placement of several tribes endemic to Lake Tanganyika (e.g. *Boulengerochromis* and Bathybatini incl. Hemibatini formed a monophyletic clade; the monophyly of the benthopelagic LT clade (see below) was not recovered; a clade composed of Eretmodini, Ectodini and Lamprologini was resolved as the sister group to Haplochromini; Tropheini and *Serranochromis macrocephalus* were resolved as sister taxa). In general, nodes of the topology based on the nuclear dataset were weakly supported as compared to the mitochondrial based topology.

The divergence time estimates based on the different calibration sets of the full mitochondrial alignment differed only slightly from each other. Divergence ages based on the Calibration Set 1, Set 2, Set 5, Set 7, Set 9 and Set 11 (with the root age range of 46–174.78 Ma) were only slightly older and had a wider 95% HPD interval than those based on the calibration Set 3, Set 4, Set 8, Set 10, Set 13, Set 14 and Set 15 (root age range of 82.17–98.91 Ma). Application of a log-normal distributed prior for the root (Set 2) or a uniform distribution of the root (Set 11) had only marginal impact on node ages, which were only slightly older for Set 11. If no root calibration was applied (Set 16), divergence ages were slightly older than those of Set 4 but their 95% HPD intervals still widely overlapped, even so they were generally wider than those of calibration Set 4. On the other hand, divergence ages of our non-rooted calibration set were younger than those of Set 2 but again 95% HPD intervals of both sets overlapped to some extent.

Three alternative placements of the fossil †*Tugenchromis*, i.e. either including the C-lineage and Eretmodini (Set 1 and Set 3) or excluding Eretmodini (Set 2 and Set 4) or alternatively at the base of the EAR (Set 9 and Set 10), had marginal impact on divergence age estimates, too. Divergence ages based on calibration sets without †*Tugenchromis* (Set 5 and Set 6) usually yielded slightly older ages than calibrations sets including †*Tugenchromis*. Likewise, divergence ages obtained by calibration sets excluding †*Oreochromis lorenzoi* (Set 7 and Set 8) were slightly older than divergence ages based on comparable calibration sets including the fossil (Set 2 and Set 4). The same was true for the calibration Set 13 excluding the neotropical cichlid fossil *†Tremembichthys,* which yielded only slightly older divergence ages in comparison to calibration Set 4. If *†Tremembichthys* was placed at the base of Heroini instead of Cichlasomatini (Set 17) divergence ages were revealed to be only slightly older than those of calibration Set 4. Divergence ages of the calibration set excluding the age of the Barombi Mbo maar (Set 15) as a calibration point were in general slightly older than those of the calibration Set 4, but confidence intervals overlapped widely. The exclusion of *†Gymnogeophagus eocenicus* (Set 14) as a calibration point resulted in marginally younger divergence age estimates in comparison to those of calibration Set 4.

The small impact on divergence age estimates of both taxa might be explained by the fact that we applied six additional calibration points, including one root calibration point. Root calibration points are affecting time estimates more than shallow ones and estimates become more consistent when multiple calibration points are applied [85, 86].

**Fig. 3:**
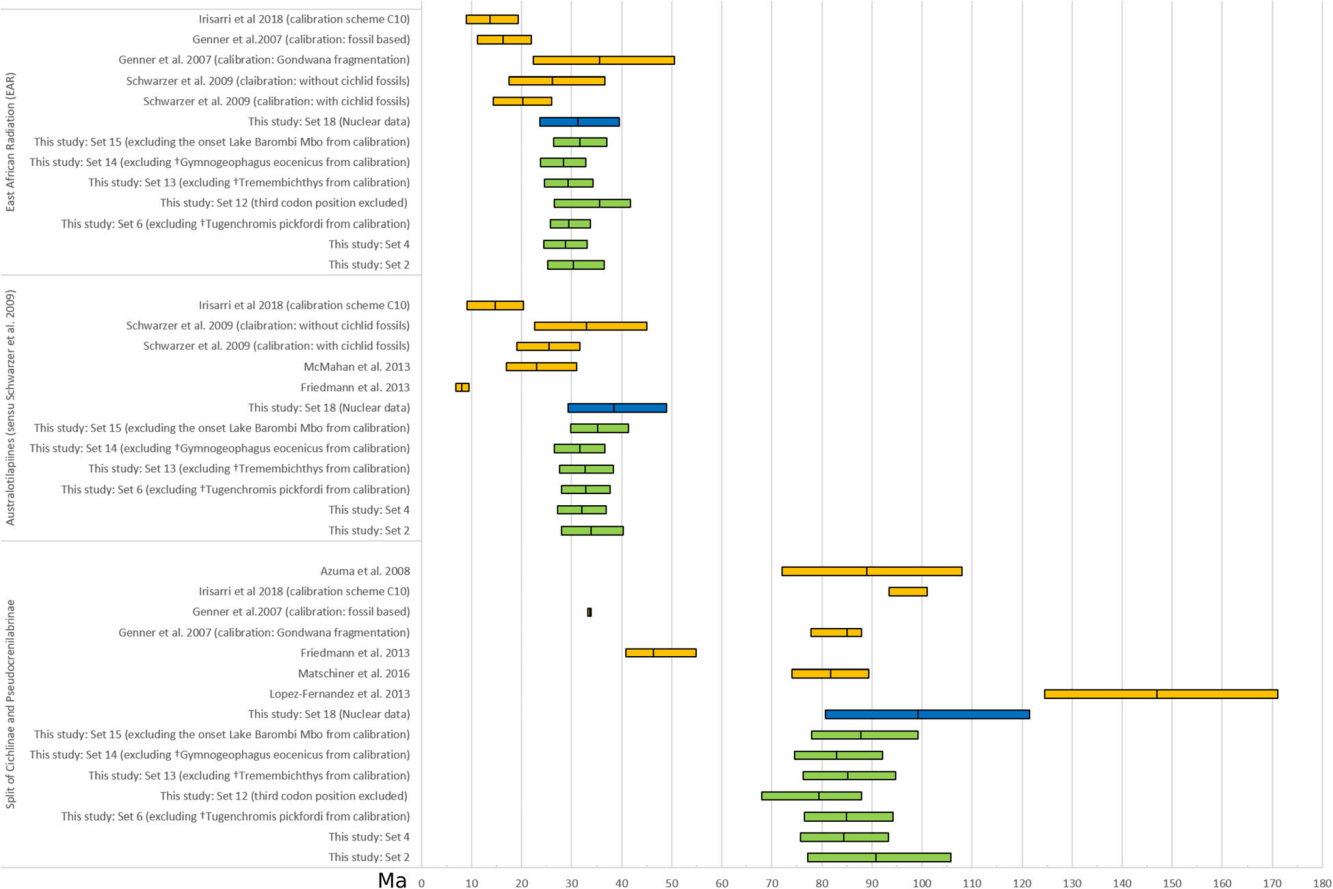
Overview of divergence age estimates from this study. Calibration Sets based on the mitochondrial dataset (Set 2, Set 4, Set 12, Set 13, Set 14 and Set 15) are depicted in green. Calibration Set 18 based on the nuclear dataset is depicted in blue and several previous studies for selected cichlid groups (Cichlinae and Pseudocrenilabrinae, austrotilapiines and the East African Radiation) are depicted in orange. Depicted are either 95% HPD (highest posterior intervals), 95% credibility intervals, 95% confidence intervals or standard deviations depending on the source study. Mean ages are indicated by middle bar of each interval

**Fig. 4:**
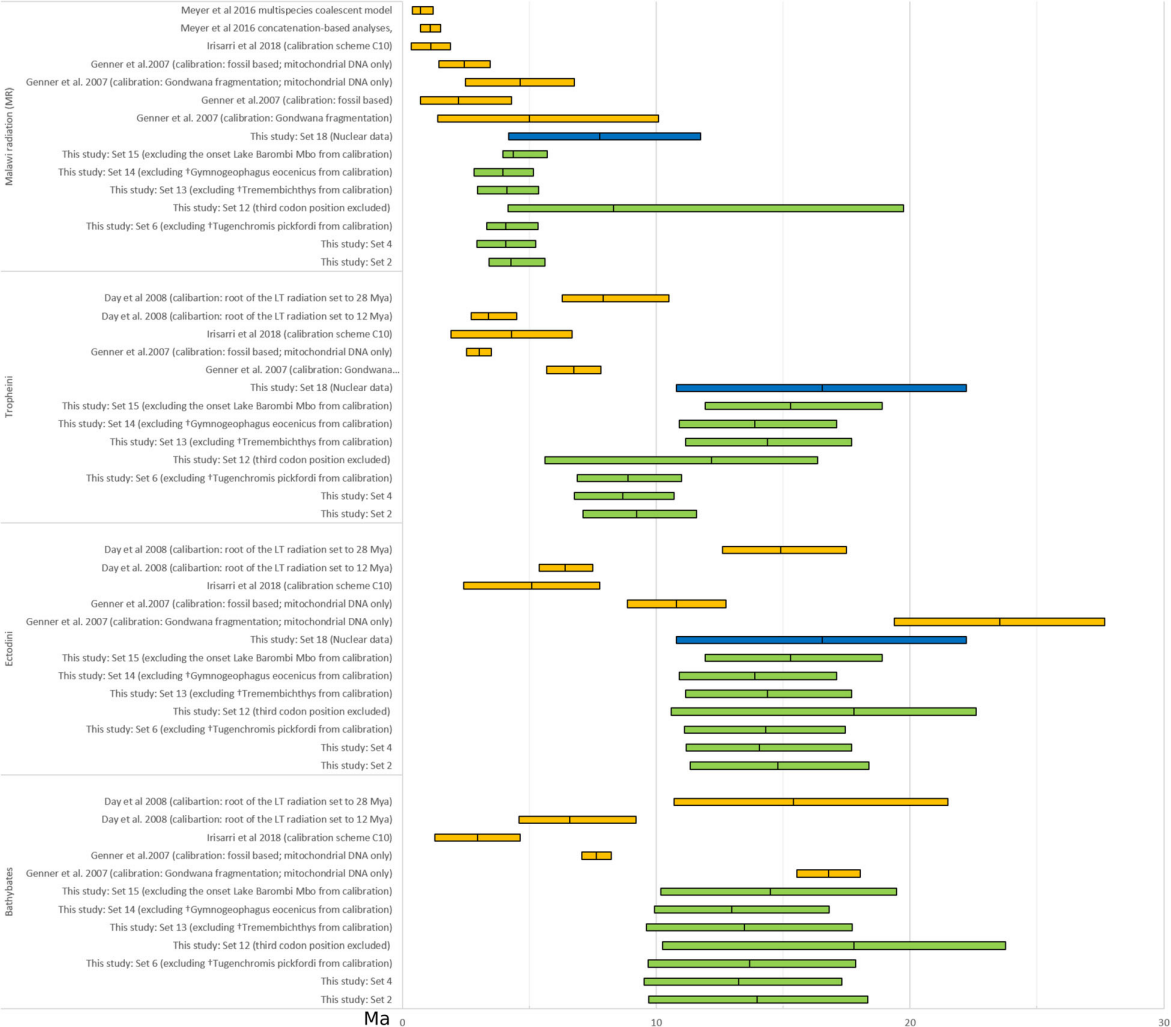
Overview of divergence age estimates from this study. Calibration-Sets based on the mitochondrial dataset (Set 2, Set 4, Set 12, Set 13, Set 14 and Set 15) are depicted in green. Calibration Set 18 based on the nuclear dataset is depicted in blue and several previous studies for selected cichlid groups (*Bathybates*, Ectodini, Tropheini and the Malawi radiation) are depicted in orange. Depicted are either 95% HPD (highest posterior intervals), 95% credibility intervals, 95% confidence intervals or standard deviations depending on the source study. Mean ages are indicated by middle bar of each interval

The divergence times of deep nodes (e.g., the root of Cichlidae; split of Pseudocrenilabrinae and Cichlinae; crown age of Pseudocrenilabrinae; crown age of Cichlinae) of the calibration Set 12 (mitochondrial alignment with third codon positions removed) were comparatively younger than those of the corresponding Set 4 (third positions included) but nevertheless widely overlapped with their 95% HPD intervals (see Figs. 3 and 4). These younger estimates for comparatively old nodes in Calibration Set 12 contrasted with comparatively older divergence time estimates of shallower nodes (i.e., EAR, Tanganyika tribes, haplochromine lineages) in the same analysis. Further, divergence ages of Set 12 had wider 95% HPD ranges, especially those of more recent splits (e.g., Lake Malawi species flock divergence). In summary, we could not detect severe effects of basal branch compressions by including the partially saturated third codon position in our analyses but rather found even younger age estimates for shallow nodes when doing so. Therefore, we conclude that despite partial saturation of third codon positions, their exclusion had no drastic impact on node age estimates but actually removed informative data, which were particularly relevant for resolution of shallower nodes. Divergence time estimates based on the comparative nuclear dataset with calibrations as for Set 4 had wider 95% HPD ranges and were generally older than corresponding estimates of the mitochondrial data set with calibration Set 4 (Figs. 3 and 4). However, 95% HPD intervals overlapped widely, sometimes even completely, as e.g. for the MRCA of the EAR. In summary, divergence estimates based on the nuclear dataset did not contradict the results of the mitochondrial datasets. Furthermore, these findings suggest that the use of only nuclear versus mitochondrial data alone is not entirely responsible for older divergence estimates observed on previous studies using mitochondrial data only.

A comprehensive list of mean divergence ages and their corresponding 95% HPD age ranges of selected nodes is given in the Additional file 4: Table S2. Here, we focus on age estimates obtained by the Calibration Set 4 because alternative estimates were highly similar and because Set 4 represents in our view the most likely setting, as the root calibration was constrained based on the most comprehensive data set [9], and it accounted for the more likely placement of †*Tugenchromis* [28]. We may want to point out that our age estimates are mitochondrial haplotype divergence ages, which do not fully reflect speciation events, but rather are a first solid hint to minimum divergence ages. Moreover, comparatively young divergence age estimations (especially those younger than 1 Ma) might be inaccurate and most probably overestimate the actual diversification ages due to several reasons: For instance divergence time estimations are influenced by to the time-dependence nature of molecular rates which are reflected by the fact that there is a measurable transition from low, long-term substitution rates to increased, short-term mutation rates, most likely as a result from multiple factors (e.g., purifying selection, ancestral polymorphism) but also due to sequencing errors and calibration errors that can account for time-depended molecular rates [5, 87, 88].

### Mitochondrial phylogeny and divergence time estimates of selected lineages

The ML-analysis (Fig. 1) and all BEAST-analyses recovered the monophyly of all recognized cichlid subfamilies, and additional major lineages and general relationships are consistent with most previously published studies (e.g. [5, 7–9]). The Etroplinae outgroup (Madagascar, southern India and Sri Lanka) formed the sister group to all other Cichlidae, and Ptychochrominae (Madagascar) were recovered as a sister group to a weakly supported clade of African Pseudocrenilabrinae + Neotropical Cichlinae (BS: 64). Mean divergence age (calibration Set 4) of the MRCA of African Pseudocrenilabrinae and Neotropical Cichlinae were estimated to be of Late Cretaceous age: 84.37 Ma (95% HPD: 75.71–93.25 Ma). Monophyly of Cichlinae was well supported (BS: 100) and the MRCA divergence age estimate is dated to 73.93 (95% HPD: 66.27–82.33 Ma). Internal relationships of tribes and lineages of Cichlinae were widely congruent with previous studies except for the poorly supported monophylum of Chaetobranchini and Astronotini (BS: 32), which was recovered as a sister group of the Cichlasomatini + Heroini monophylum. Similarly, monophyly of Pseudocrenilabrinae was well supported (BS: 100), but the divergence age of Pseudocrenilabrinae MRCA in our dataset was dated younger than that of Cichlinae, i.e. 60.79 Ma (95% HPD: 50.87–71.10 Ma). However, the estimate would have been substantially older if *Heterochromis,* which is the early branching sister group to all remaining Pseudocrenilabrinae, had been included (eg. [7, 8]). Thus, MRCA age estimates for two cichlid subfamilies Pseudocrenilabrinae and Cichlinae are largely compatible with several previous studies, e.g. [5] (based on Gondwanan landmass fragmentation), [6] (2008; based on 21 teleost fossils of different lineages) and [9] (based on 147 fossil clade age calibration points).

Intrarelationships of major African cichlid tribes (tylochromines, chromidotilapiines, hemichromines pelmatochromines) were only poorly supported as it was the case in previous studies (e.g. [5, 35, 89]. Monophyly of haplotilapiines (sensu [67]) was, however, well supported (BS: 100) with an estimated Eocene divergence age of 45.38 Ma (95% HPD: 37.98–54.49 Ma). Within haplotilapiines, Oreochromini (BS: 88) and austrotilapiines (BS: 38) were recovered as sister groups for the first time based on mitochondrial data alone, albeit with very weak support (BS: 41). Monophyly of boreotilapiines was not recovered in our analysis. Divergence age of the MRCA of Oreochromini was dated to 22.95 (95% HPD: 17.27–29.11 Ma) and of austrotilapiines to 31.98 Ma (95% HPD: 27.17–36.92 Ma). Within austrotilapiines, Steatocranini were resolved as a sister group to the EAR with relatively high support (BS: 94) and the divergence age of the MRCA was estimated to 30.62 Ma (95% HPD: 26.59–35.40 Ma).

Monophyly of the EAR was well supported (BS: 100) and the onset of divergence for this lineage was estimated to be of Late Eocene/Early Oligocene age: 28.71 Ma (95% HPD: 24.43–33.15 Ma). Boulengerochromini were recovered as the earliest diverging EAR lineage followed by a strongly supported clade (BS: 100) of Bathybatini + Trematocarini. This is congruent with two previous mtDNA studies (e.g. Day et al. 2008), but contrasts with other mtDNA studies which retrieved Boulengerochromini, Bathybatini and Trematocarini as the sistergroup to the remaining lineages of the EAR (e.g. [36, 90, 91]. Trematocarini were estimated to have diverged 16.13 Ma ago (95% HPD: 11.89–20.46 Ma) while Bathybatini started diverging 20.62 Ma (95% HPD: 16.88–25.34 Ma). In contrast with previous studies, which found Lamprologini and Eretmodini to form a sister group to the remaining members of EAR (e.g. [14, 17, 36, 76] Lamprologini were resolved as the sister group to the H-lineage (C-lineage including the Eretmodini) in our analyses. Divergence of the MRCA of Lamprologini and the H-lineage was well supported (BS 100) and estimated to 23.6 Ma (95% HPD: 20.18–27.33 Ma).

According to our data, Lamprologini diverge into three strongly supported (BS: 100) lineages during the Miocene at around 15.27 Ma (95% HPD: 12.23–18.49 Ma). The first clade was composed of the ‘non-ossified Lamprologines’ with taxa mainly endemic to LT but included some riverine taxa of disjunct distributions in the Congo Basin, e.g. *L. werneri* and *L. symoensi*. This clade diverged at around 12.51 Ma (95% HPD: 9.75–15.51). The second Lamprologini clade was composed of the LT endemics belonging to the ‘ossified Lamprologines’ and diverged at around 10.66 Ma (95% HPD: 7.39–13.97 Ma). Surprisingly and for the first time a mtDNA clade encompassing only *Lamprologus* of the lower and central Congo drainage (*L. mocquardi*, *L. markerti*, *L. tigripictilis*, *L. lethops*, *L. teugelsi* and *L.* sp. Kwango) was recovered; we refer to it as the ‘Lower Congo *Lamprologus* clade’, because most members of this clade are only known form the Lower Congo area. Its divergence was dated substantially younger than the other two clades, i.e. to Late Miocene or early Pliocene at around 6.62 Ma (95% HPD: 4.31–9.49 Ma). Interrelationships of the three lamprologine clades were poorly supported.

Monophyly support for each of the ancient Tanganyika mouthbrooder tribes (Cyphotilapiini, Limnochromini, Ectodini, Perissodini, Benthochromini and Cyprichromini) was strong (BS: 100). For the first time a clade composed mostly three pelagic or epibenthic clades Perissodini, Cyprichromini and Benthochromini was recovered with strong support (BS 100), based on mitochondrial markers,. We refer to this clade as the “benthopelagic LT clade”. It was only weakly supported in previous mtDNA based studies [36, 92], but is well supported by nuclear DNA data and more recently by AFLP and RAD based studies [7, 17, 93]. Divergence of the MRCA of the benthopelagic LT clade was dated to the Middle to Early Miocene age: 16.14 Ma (95% HPD: 12.83–19.54 Ma). Divergence of Perissodini took place at around 6.18 Ma (95% HPD: 3.16–9.59 Ma), of Cyprichromini at around 10.38 Ma (95% HPD: 6.20–14.53 Ma) and of Limnochromini at around 10.71 Ma (95% HPD: 5.20–16.30 Ma). Further, monophyly of Eretmodini were recovered with strong support (BS 100) and their divergence age is 7.60 Ma (95% HPD: 3.92–11.99 Ma) which was comparable with those of Perissodini. In contrast, Ectodini and Cyphotilapini diverged slightly earlier with mean ages of 14.06 Ma (95% HPD: 11.18–17.70 Ma) and 14.16 Ma (95% HPD: 8.71–19.25 Ma), respectively.

The Malagarasi-*Orthochromis* are recovered as the sister group of Haplochromini with moderate support (BS 71), which contrasts with the placement of Malagarasi-*Orthochromis* of previous mtDNA based studies, which recovered for example a relationship of Malagarasi-*Orthochromis* and Ectodini (e.g. [15, 76]). Several nuclear DNA based studies however recovered the Malagarasi-*Orthochromis* as a sister group of the Haplochromini as is the case in this study (e.g. [17, 76]). Monophyly of Haplochromini was highly supported (BS 100) and the onset of diversification of Haplochromini was dated to Early Miocene: 16.64 Ma (95% HPD: 14.25–19.16 Ma). ‘*Ctenochromis*’ *pectoralis* from the Pangani River drainage (Tanzania, Kenya) was placed as a sister group to all remaining Haplochromini with high support (BS 100), hereby confirming previous studies which however only found poor support for this node ([15, 26, 94].

Intrarelationships and monophyly of previously recognized haplochromine mtDNA lineages (e.g serranochromines-mt-lineage s.l.; *‘Pseudocrenilabrus-*group’ incl. Northern-Zambian-*Orthochromis*, ‘New Kalungwishi cichlid’, ‘New Lufubu cichlid’; *Astatoreochromis*, LT Tropheini; Lake Malawi species flock and ‘modern Haplochromini’ incl. Lake Victoria Region Superflock and riverine east and central African haplotypes) were in large part congruent with previous mtDNA based studies (e.g. [15, 37, 39]. The serranochromines-mtDNA-lineage sensu *lato*, i.e. the southern-central African lineage including the Lake Fwa cichlids and ‘*O*.’ *stormsi* (mean age: 13.41 Ma; 95% HPD: 11.18–15.85 Ma) are estimated to be slightly older than all remaining major lineages, i.e. the *‘Pseudocrenilabrus-*group’ (mean age: 11.82 Ma; 95% HPD: 9.63–14.18 Ma), Tropheini (mean age: 8.69; 95% HPD: 6.77–10.70 Ma), ‘modern Haplochromini’ (mean age: 9.42 Ma; 7.72–11.23 Ma) and Lake Malawi species flock (mean age: 4.07 Ma; 2.93–5.26 Ma). The well supported (BS 99) clade encompassing the serrranochromines-mtDNA-lineage *s. str.* (following Joyce et al. [95] and Musilová et al. [39]) represented in our study by *Serranochromis robustus*, *S. altus*, *Pharyngochromis sp*. and the undescribed taxon *‘Haplochromis’* sp. Kwango are of Pliocene to Early Late Miocene age: 5.46 Ma (95% HPD: 3.79–7.24 Ma). The Lake Victoria Region Superflock (LVRS, following Verheyen et al. [96] and was recovered with strong support (BS: 100) and its divergence started in the Pleistocene age: 0.31 Ma (95% HPD: 0.12–0.53 Ma). In addition to these lineages, two novel mitochondrial haplotype lineages within Haplochromini were recovered here for the first time. ‘*Orthochromis*’ *indermauri* was recovered as the sister lineage of a clade encompassing the *‘Pseudocrenilabrus-*group’ and the ‘ocellated eggspot Haplochromini’ (BS: 85). It is endemic to rapids on the lower Lufubu, the largest affluent of the southern Lake Tanganyika basin ([97]). Divergence of ‘*Orthochromis*’ *indermauri* and remaining Haplochromini was dated to around 15.15 Ma (95% HPD: 12.91–17.46 Ma). The second novel lineage was ‘*Haplochromis’ vanheusdeni* from the Great Ruaha drainage system, which was recovered with strong support (BS: 96) as sister taxon to all LT endemic Tropheini, a subgroup of Haplochromini. Divergence of this East African coastal drainage species and LT Tropheini was estimated to have taken place in the Miocene at around 10.51 Ma (95% HPD: 8.47–12.61 Ma).

Even so a large fraction of the nodes of the ML tree was well supported (BS: 100), it is worth mentioning that several nodes were comparatively weakly supported (Fig. 1). Most of the latter referred to early diversification events within Pseudocrenilabrinae and Cichlinae, or they are located among rapidly diversifying EAR clades, e.g. among early diverging EAR-tribes or among haplochromine cichlids. In contrast, different BEAST analyses resolved many more nodes with high support supported (BBP = 1) and consequently comparatively fewer nodes had low support (see e.g. Figure 2). This contrast might have several reasons. Generally, Bayesian analyses tend to yield on average higher node support than ML analyses and therefore might be overoptimistic ([98, 99]). Moreover, in the addition to the different calibration points we predefined five monophyla based on the ML analysis in our BEAST analyses, which may have strengthened BPPs for nodes related to these phylogenetic constraints.

## Discussion

The present study represents a comparatively comprehensive and robust data set in terms of number of mitochondrial markers (10 coding genes) and taxa (180 species of Cichlidae), with a novel calibration set including the recently described fossil species (†*Tugenchromis*; [28]). We recovered novel mitochondrial haplotype phylogenies based on the improved taxon sampling, which in combination with novel and re-evaluated node age estimates allow for a refined phylogeographic view on the origin and diversification of Cichlidae, especially those of the EAR.

### Divergence age estimates in comparison of previous studies

Overall, our attempts to date the evolutionary history of cichlids based on the conservative selection of five well-corroborated fossils, one geological and two alternative root calibrations yielded robust divergence age estimates. These are congruent with several preceding studies while other studies resulted in partially different divergence age estimates. These discrepancies can be partially attributed to different calibration priors. By integrating extreme age estimates from previous studies into our alternative root age calibration strategy, we endevaored to evaluate these results with the background of our conservative internal node calibration strategy.

Divergence age estimates for two cichlid subfamilies Pseudocrenilabrinae and Cichlinae of this study are largely compatible with several previous studies (e.g. [5, 6, 9]). In contrast, they are in more or less dramatic conflict with other studies (Fig. 3), substantially with those López-Fernandez et al. [13], Friedman et al. [7], less dramatic with studies by McMahan et al. [8, 100] and Irisarri [101], and partially with some partial results presented by Genner et al. [5] and Day et al. [14].

López-Fernandez et al. [13] had estimated much older divergence age for Cichlidae (mean: 147 Ma, 95% HPD: 124.49–171.05 Ma), but their age estimates were recently challenged as they predate the oldest first spiny-rayed teleost (Acanthomorpha) and because multiple taxon concatenations in their alignment appeared to be composed of sequences from different taxa [52, 102]. At the other extreme, Friedman et al. [7] estimated a much younger divergence age for the MRCA of Pseudocrenilabrinae and Cichlinae (mean: 46.4 Ma, 95% HPD: 40.9–54.9 Ma). They had used ten non-cichlid fossils for calibration and no cichlid fossil. Again, such young divergence ages were questioned later [52] because they strongly contradicted the fossil record. The oldest Lumbrera formation cichlid fossils are at least 39.9 Ma old, which is considerably older than Friedman et al.´s (2013) divergence age estimate for Cichlinae (=Neotropical cichlidae) with a mean age of 29.2 Ma (95% CI: 25.5–34.8 Ma). Finally, application of one of the two calibration sets of Genner et al. (2007) using seven cichlid fossils resulted in substantially younger node age estimates than ours and also partially conflicts with the fossil record. Their age estimate for divergence of Pseudocrenilabrinae (33.6 Ma, 95% HPD: 33.2–33.9 Ma) substantially postdated the oldest known African cichlid fossil by about 12 million years (†*Mahengechromis* – 46 Ma); and the age of the MRCA of Cichlasomatini was younger (14.2 Ma; 95% HPD: 7.6–21.1 Ma) than the oldest known fossil for this tribe, i.e. †*Tremembichthys,* 55.8–23.03 Ma, although the placement of †*Tremembichthys* as a member of Cichlasomatini might be considered as problematic due to its high “Heroine-like” number of pterygiophores articulated with the first haemal arch. Such young age estimates are most likely the result of calibration with fossils which are not the oldest fossils of their respective lineage (e.g. †*Aequidens saltensis* from Argentina with an estimated age of 5.33–23.03 Ma has been used as a calibration for the entire tribe Cichlasomatini), whose priors were calibrated with hard upper and lower bounds. Moreover, following Malabarba et al. [103] they placed †*Proterocara argentina* from the Lumbrera formation as the earliest member of a clade uniting Geophagini, Cichlasomatini and Chaetobranchini; later however, Smith et al. [104]) revised †*Proterocara argentina* to be related with *Crenicichla* and *Teleocichla*. In addition, the use of further fossils with very uncertain phylogenetic placements like ? *Tylochromis* [105] and ? *Heterochromis* [106] as a calibration points in the analyses of Genner et al. [5] might have led to these young divergence age estimates obtained in their study. A more recent study by Meyer et al. [100] was based on two different divergence estimation methods and two secondary constraints taken from results of McMahan et al. [8]. Divergence ages obtained by McMahan et al. [8] are, based on calibrations with four cichlid fossils and one early acanthomorph fossil, in large parts compatible to our divergence estimates in the range of the 95% confidence intervals; their mean age estimates are, however, consistently younger than ours. One possible explanation for the younger estimates of McMahan et al. [8], and consequently that of Meyer et al. [100], is a that they placed two neotropical fossils †*Plesioheros* and †*Tremembichthys* at the basis of a clade consisting of the Heroini and Cichlasomatini, which is different from our placement accepting †*Plesioheros* as Heroini and †*Tremembichthys* as Cichlasomatini. Finally, the only study using the recently described fossil †*Tugenchromis pickfordi* as a calibration point obtained for basal divergence events, e.g. the divergence of Pseudocrenilabirinae and Cichlinae, older estimates but for more recent events substantially younger estimates as compared to ours (Figs. 3 and 4) [101]. This study was based on an anchored loci approach with 533 nuclear loci for a total of 149 taxa. Due to their massive dataset, the usage of the software BEAST [48] for inference of divergence time estimates was not possible and therefore the non-Bayesian method RelTime [107] was used ([101]). The discrepancies in the divergence time estimations between this and our study might be partially attributable to the use of different analytical approaches as implemented in the different software packages, since RelTime divergence time estimates for comparatively old nodes appear to be inferred with a strict clock model, which was subsequently contradicted by a recent study of the software developers [29, 108, 109]. Apart from this disputable feature of RelTime, it is worth mentioning that RelTime only allows for hard boundaries for age constraints, and those were applied in the study of Irisarri et al. [101] partially for fossils with disputable phylogenetic placement, i.e. †*Tylochromis* (see discussion of this fossil in Additional file 3), or with conservative maximum age boundaries secondarily taken from the Gondwana-set of the study of Genner et al. [5]. Therefore, it would be interesting if a Bayesian analysis with a reduced dataset with a comparable calibration scheme, as suggested by Matschiner [29], would yield comparable results to ours.

In contrast to the aforementioned conflicts with previous studies, our divergence age estimates, especially those for the age, origin and diversification of the EAR, are compatible with results from other studies, i.e. the Gondwana breakup calibration inference of Genner et al. [5] and Day et al. [14] Day et al. (2008) and the fossil based inference of Schwarzer et al. [35]. In the light of the recently published findings and overlooked calibration problems, conflicts of our age estimates with previous studies appear explainable. Since our study conservatively incorporates carefully selected calibration points, includes for the recently described EAR fossil (†*Tugenchromis)*, and carefully accounts for remaining uncertainties by evaluating alternative placements of critical fossils in molecular clock analyses, we provide an improved framework for the discussion of the phylogeographic history of the exact cichlid diversity, in particular the one of East African cichlids of Lake Tanganyika.

### Divergence age estimates of Pseudocrenilabrinae and Cichlinae favor a short-distance dispersal scenario across the emerging proto-Atlantic

The recent geographic distribution of the two reciprocally monophyletic cichlid subfamilies Cichlinae (Americas) and Pseudocrenilabrinae (Africa) is a matter of the long-standing debate. Such a pattern can be interpreted as a result of either the Gondwana breakup (“Vicariance Hypothesis”), or, alternatively, by a trans-Atlantic dispersal event (“Marine Dispersal Hypothesis”) if the radiation of extant Cichlinae and Pseudocrenilabrinae took place after the fragmentation of Gondwana. [5–8, 11, 12, 29, 110]. Unfortunately, evaluation of these alternative hypotheses has been and still remains difficult. This is due to the different geological age estimates for the final separation of South America and Africa, which according to recent estimates took place at around 103 Ma at the Ghanaian Ridge and the Piauí-Ceará margin [111]. Genner et al. [5], for example, calibrated the South America and Africa separation with a range of 86 to 101 Ma whereas Azuma et al. [6] calibrated the same event with 100 to 120 Ma. The most comprehensive previous study dates the separation of Cichlinae and Pseudocrenilabrinae to 81.8 Ma (95% HDP: 89.4–74.0 Ma), i.e. a few million years thereafter [9]). The latest comprehensive review on this discussion argues that the divergence of Pseudocrenilabrinae and Cichlinae occurred probably around 60 to 75 Mya after evaluating potential sources creating observed differences in divergence time estimation studies [29].

Our divergence age estimates for the split of Neotropical and African cichlids (84.37 Ma (95% HPD: 75.71–93.25 Ma) tentatively support the “Marine Dispersal Hypothesis”, which is in accordance with Vences, Friedman et al. [7], and Matschiner [29], as well as with, importantly, one of the most comprehensive teleost-scale study [9]. Nevertheless, our age estimates are older than the estimate of 65 to 75 Ma for the recently suggested trans-Atlantic dispersal event of cichlids [29]. If the log-normal or uniform root prior including the extremely old age ranges for the cichlid origin (prior range of 46.0–174.78 Ma) are taken into account, the combined minimum and maximum 95% HPD ranges of all our estimated scenarios is 75.61 and 107.83 Ma, i.e. it slightly overlaps with the period of the final separation of the two continents (103 Ma), but the mean ages (84.38 to 91.94 Ma) clearly postdate the split event. Nevertheless, it is important to stress here that the nascent southern Atlantic was only a few hundred kilometers wide around that time of the continent split [111], such that freshwater plumes of several large rivers (e.g. the proto-Congo or proto-Niger River) most likely extended far offshore into the narrow oceanic gap [112], and that multiple island clusters existed there along the Rio Grande Rise and the Walvis Ridge until approx. 30 Ma ago [113, 114]). In combination, these factors imply an island-hopping scenario of euryhaline cichlids over comparatively short distances rather than a long-distance marine dispersal. This inference is supported by the fact that not a single oceanic cichlid species is known today. In contrast, quite a few members of several distantly related cichlid lineages are inshore brackish water species [63, 115, 116] or are known from hypersaline inland habitats [72, 117]. Further, Matschiner [29] argues that even longer distances (650–900 km) might have been possible to cross. Alternatively, a vicariant origin of Cichlinae and Pseudocrenilabrinae cannot be falsified completely, if the 95% HPD intervals are taken into account.

### Divergence of the Lake Tanganyika tribes supports the “melting-pot Tanganyika” hypothesis

Considering our age estimates for the Lake Tanganyika (LT) cichlid tribes and all estimated ages for the formation of a LT basin (e.g. 5.5 Ma or 12 Ma) that could have served as a habitat for lacustrine cichlid radiations an intra-lacustrine origin of divergence for the LT tribes appears highly improbable. For instance, our age estimates for the MRCA of the EAR but also of the two MRCA of the most ancient Lake Tanganyika tribes (e.g., Bathybatini - 20.62 Ma, and Trematocarini - 16.13 Ma), and the MRCA of Lamprologini and H-lineage (23.6 Ma) are estimated to be substantially older than 12 million years. Hence, they predate the often-cited maximum age of LT of 12 Ma, which itself might even represent an overestimated age for the origin of the extant LT basin as those estimates are based on the probably incorrect assumption of uniform sedimentation rates (see below). Likewise, divergence age estimates for the MRCA of H-lineage and the MRCA of the benthopelagic LT clade are substantially older than the maximum estimate for the origin of LT. Ectodini and Cyphotilapiini mean divergence age estimates are still around two million years older than 12 Ma. While Cyprichromini and Limnochromini divergence ages fall in the time range of the older maximum age estimate of LT (9–12 Ma), the estimates were still older than the younger estimate of 5.5 Ma for the age of LT. Only the MRCA of Perissodini and Eretmodini had 95% HPD intervals which were partly younger than 5.5 Ma but mean ages still remain slightly older. It is worth mentioning that several of these lineages with a clear lacustrine ecology (such as Bathybatini, Trematocarini and the pelagic LT clade) started to radiate, according to our data, well before the onset of LT basin formation, even though their extant diversity evolved with high probability later under lacustrine conditions.

There is an ongoing debate about the geological age of the Lake Tanganyika basin and the onset of persistent lacustrine conditions which would have allowed for the evolution of the lacustrine species flocks of the EAR [17]. In the cichlid literature, the most commonly cited maximum age of the opening of the oldest central segment of the proto-Lake Tanganyika is 9 to 12 Ma [18]. This estimate is based on extrapolation of Quarternary sedimentation rates on seismically inferred deep-lake sediment layers assuming roughly uniform sedimentation rates [18, 118]. The northern Bujumbura LT basin and the southern Mpulungu LT basin are estimated to be younger with of ages of 7–8 Ma and 2–4 Ma, respectively. In contrast to the assumption of uniform sedimentation rates, episodes of regional tectonic, volcanic and climatic changes in the LT area rather suggest that sedimentation rates strongly fluctuated in the past and were higher especially during the late Miocene/early Pliocene. This would potentially translate into overestimated dates for the origin of lacustrine conditions of LT [17, 19]. Indeed, Cohen et al. [18] already stipulated that their age estimates are only maximum ages, and several more recent studies based on thermochronology and sedimentology date the onset of pre-rift formation of the Albertine Rift to 4–11 Ma and the earliest onset of true rifting activity that could possibly have created deep rift lakes in the northern basins to only 5.5 Ma [20–23]. Due to the complex geological history and the remaining uncertainties regarding the age of LT we will compare both age estimates (9–12 Ma and 5.5 Ma) of the origin of LT with our divergence age estimates.

Several hypotheses of the origin and timing of diversification of Lake Tanganyika cichlid tribes have been proposed over the past years. One scenario postulates that the diversification of Lake Tanganyika lineages took place within the limits of the extant LT basin, i.e. the older lineages formed during the proto-LT phase, (9–12 Ma), whereas younger tribes would have evolved in the extant lake [90]. Genner et al. [5], based on their molecular clock analysis calibrated using Gondwana fragmentation (see above), suggested LT to be a reservoir of multiple ancient riverine lineages, which adaptively radiated into lacustrine species flocks after the proto-LT area had changed to become a rift lake; this, however, occurred without leaving any riverine descendants. In contrast to the two former scenarios, the recently proposed “Melting Pot Tanganyika” hypothesis of Weiss et al. [17] proposes an independent pre-rift diversification of several LT cichlid precursor lineages in different drainages and precursor lakes of the greater LT area. After river captures in the Neogene and Pleistocene, i.e. during a phase of tectonic rearrangements in a highly dynamic and heterogeneous LT area landscape, secondary contact of those divergent cichlid lineages led to hybridization among them. Support for this scenario comes from evidence for a reticulate phylogenetic history of several LT lineages [17, 119], and, more recently, from the discovery of a Miocene age EAR fossil in Kenya with a mosaic of characters, of which some are present today only in selected LT cichlid tribes [28].

Overall, our divergence age estimates are compatible with both the hypotheses of Genner et al. [5] and Weiss et al. [17], i.e. that LT might represent a reservoir of multiple ancient lineages that have evolved before the origin of the extant LT Tanganyika basin. In combination with the discovery of †*Tugenchromis pickfordi* in the Lake Baringo area of Kenya and the recent evidence for introgression and hybridization between several ancient LT and extant riverine cichlid lineages but also among LT lineages themselves [17, 100] the “melting-pot Tanganyika” hypothesis appears to be favorable. Even though our study provides comparatively robust age estimations for the MRCA of different LT tribes, no age estimates for potential introgression and hybridization events can be provided as only maternally inherited mtDNA data were used in this study.

### Divergence of riverine Lamprologini supports several dispersal events from LT region towards the Congo system

The present study identified for the first time a third basal lamprologine mtDNA clade, whose primary divergence took place latest at around 15.27 Ma (95% HPD: 12.23–18.49 Ma) and hence predate the origin of the extant LT basin under any geological scenario. Interestingly, the third novel clade comprises only lower and central Congo taxa, whereas the two previously known clades contain both Congo basin and LT taxa, with the Congo taxa being deeply nested within the LT species community. Unfortunately, alternative relationships among the three main clades are only weakly supported, rendering any vicariance-based inference about the geographic origin of Lamprologini difficult.

Two different scenarios had previously been suggested for the origin and distribution of Lamprologini. The first suggests that Lamprologini evolved within Lake Tanganyika as a single radiation and subsequently colonized the Congo Basin, possibly via the Lukuga River [90, 120–122]. This scenario had been suggested because *Lamprologus* species of the Congo and Malagarasi-drainage are consistently nested deep within the ‘non-ossified Lamprologines’ of Lake Tanganyika in several studies (e.g. [90, 120–122]). In contrast, the study of Clabaut et al. [76] identified a clade encompassing a sample identified as *L. teugelsi*^1^ and *L. congoensis* as the sister group of all remaining LT Lamprologines in their nuclear DNA data set. This phylogenetic result suggested that Congo Lamprologini seeded the LT Lamprologini radiation, and hence rendering Congo Lamprologini ancestral relict species.

According to the results presented herein, the crown age of the two lineages harbouring predominantly LT taxa (the ‘ossified’ and ‘non-ossified’ Lamprologines), is substantially older than that of the Congo-lineage, a geographic origin of Lamprologini in the proto-LT region appears more likely and hence they appear to have colonized the western and central Congo basin later through multiple dispersal events. A first colonization event in the Late Miocene to Early Pliocene might have seeded the ‘Lower Congo *Lamprologus* clade’; and, interestingly, it falls in the same time range of previously estimated age of the MRCA of the lower Congo endemic radiation of *Nanochromis* and *Steatocranus* [123]. The diversification of the ‘Lower Congo *Lamprologus* clade’ might therefore be linked to the Pliocene origin of the modern lower Congo River rapids, which has been suggested to be correlated with the species-flock formation of *Steatocranus* and *Nanochromis* in the same area [123]. If the Lamprologini origin in the greater LT region is correct, and if the Lower Congo Lamprologini originally were monophyletic as suggested by morphology [124], then only a second colonization event could explain the alternative mtDNA haplotype placement of *L. werneri* in the ‘non-ossified Lamprologines’ clade. Indeed, complete exchange of mtDNA-haplotypes is known for LT endemic Lamprologini and therefore cannot be ruled out until more nuclear data are available for this group [125, 126].

We have included for the first time in a molecular phylogenetic analysis the only Upper Congo (Lualaba) endemic *Lamprologus*, *L. symoensi* from the Upemba Lakes region. Similarly to the *L. teugelsi* case, it appears to be either a descendant of a secondary colonization event (most likely by a member of the ‘non-ossified Lamprologines’ as suggested by morphological data; see Schelly et al. [124]) or *L. symnoensi* captured the mitochondrial genome from dispersing LT Lamprologini. Interestingly, our mtDNA divergence ages estimates of *L. symoensi* and *Telmatochromis* cf. *temporalis* are young at around 2.55 Ma (95% HPD: 1.34–3.87 Ma), roughly similar to those of *Pseudocrenilabrus multicolor* and *P. nicholsi,* the former one a Nilotic species and the latter one an Upper Congo (Lualaba) species: 2.20 Ma (95% HPD: 1.20–3.34 Ma). This coincidence may indicate that the closely neighbouring Upper Congo, Lake Tanganyika and Nile drainage systems were relatively permeable at this time, e.g. through river captures and/or shared headwater areas, allowing the exchange of faunistic elements. This inference is also compatible with established Congo-Nilotic sister group relationships of selected modern Haplochromini [26].

### Age and divergence within riverine Haplochromini and their lacustrine radiations

Originally, the “Out of Tanganyika” hypothesis had suggested that the geographic and genetic cradle of Haplochromini is Lake Tanganyika and that LT Haplochromini secondarily left the lake to seed all other haplochromine radiations in East Africa [37]. Our new node age estimates in combination with an improved riverine Haplochromini taxon sampling enabled us to re-evaluate this hypothesis, as well as the biogeographic and temporal origin of several other Haplochromini radiations, i.e. the modern Haplochromines of the Lake Victoria Region superflock (LVRS), the Lake Malawi species flock, the LT Tropheini.

In line with other more recent phylogenetic studies (e.g. [9]) our molecular clock data suggesting that the onset of the Haplochromini diversification had started already by the Early Miocene (16.64 Ma; 95% HPD: 14.25–19.1). This date substantially predates the presumed tectonic origin of the LT basin by several million years and renders an “Out of Tanganyika” scenario rather unlikely based on our estimates. Taking into account that the Malagarasi-*Orthochromis* and the Haplochromini are resolved as sister lineages and that the earliest split within the Haplochromini is the sister-group relationship of *Ctenochromis pectoralis* endemic to coastal drainages in Kenya and Tanzania, and remaining Haplochromini, it seems more parsimonious that the MRCA of haplochromine cichlids lived east of LT. Nevertheless, a key role of the greater LT region as a reservoir of ancient haplochromine cichlid lineages is shown by the relict-like distribution patterns of the riverine mtDNA lineage of the recently described ‘*Orthochromis*’ *indermauri*, which is estimated to have diverged from other Haplochromini lineages including the *‘Pseudocrenilabrus-*group’, Tropheini plus ‘*H*’*. vanheusdeni*, the Lake Malawi species flock and modern Haplochromini well before the origin of the LT basin in the Early to Middle Miocene.

Undoubtedly, LT with its history of climate-driven lake level fluctuations shaped the evolution of the Tropheini (e.g. [127–129]), but the origin of this LT endemic haplochromine lineage is only partially understood. In previous studies Tropheini had been resolved as the sister group to a clade encompassing the many riverine and modern Haplochromini (including the LVRS) and the Lake Malawi species flock (e.g. [7, 37, 119]). Further, two recent studies found support for a potential ancient hybrid origin for the Tropheini [17, 100]. Therefore, it is quite unexpected that the here newly recognized lineage represented by ‘*H*’*. vanheusdeni*, endemic to the coastal Great Ruaha drainage in eastern Tanzania system, is resolved as the mitochondrial sister group to Tropheini. Divergence of those two lineages is estimated to have occurred in the early or middle Miocene, which indicates a past connection of the proto-Malagarasi drainage system and the Proto-Great Ruaha drainage system at that time. Interestingly, in addition to ‘*H*’*. vanheusdeni* is another biogeographically important lineage known from Ruaha drainage system. Genner et al. [130] recovered *Astatotilapia* sp. ‘Ruaha’ as sister lineage of the Lake Malawi species flock. Unfortunately, our study is missing this taxon, but it underlines the remarkable drainage evolution of the Ruaha.

The MRCA of the megadiverse Lake Malawi species flock is dated to the Pliocene at around 4.07 Ma (95% HDP: 2.93–5.26 Ma). This age is compatible with previous findings based on fossil and Gondwana fragmentation calibration [5], or on secondary calibrations (Genner et al. [131] based on [35]). However, our Pliocene divergence age sharply contrasts with the findings of several other studies dating the age of the Lake Malawi species flock considerably younger, i.e. 0.93–1.64 Ma [16], 0.73–1.0 Ma [15], 0.7–1.5 Ma ([100]; concatenation set) and 0.4–1.2 Ma ([100]; multispecies coalescent model). The young age estimates obtained by the studies of Sturmbauer et al. [16] and Koblmüller et al. [15] appear to be the result of a calibration based on the assumption of Delvaux [24] that Lake Malawi almost completely desiccated between 1.6 Ma until 1.0–0.57 Ma, and on the assumption that an intralacustrine origin of major Lake Malawi cichlid clades (“mbuna”, “utaka”) would have taken place only after hypothetical refilling of the LM. This assumption might be, however, inappropriate, as a recent study recorded continuous sedimentation in Lake Malawi over the last 1.3 Ma, even though 15 severe droughts had intermittently resulted in lake level decreases of more than 400 m [25]. Moreover, the geological and sedimentological age of LM is still poorly understood. The Malawian Rift is bordered by the Rungwe volcanic province, which is estimated to have formed between 5.45 to 8.6 Ma based on K/Ar dating of different volcanic materials [132, 133]. These ages are commonly associated with the onset of rifting of LM rift basin (e.g. [24, 131]). The lower Chiwondo Beds northwest of LM coast are dated to 4 Ma or older based on biostratigraphy and represent the first evidence for lacustrine conditions of LM [134]. Our divergence time estimates for the origin of the LM species flock are thus compatible with the reported onset of lacustrine conditions of LM and which would imply that ancient lineages of the LM species flock survived these droughts. Interestingly, the MRCA of the clade containing predominantly sand-dwelling genera (mean age: 0.69 Ma; 95%HPD: 0.45–0.96 Ma) and the MRCA of the clade containing predominantly rock-dwelling genera (mean age: 0.6 Ma; 95%HPD: 0.39–0.81 Ma) appear to have emerged at around the Mid-Pleistocene restoration of lacustrine conditions in LM The radiation of these clades hence may be the result of increased ecological opportunity and habitat stability in LM [25, 100].

The exact geological age of the largest freshwater lake in world, Lake Victoria (LV), is still debated, but its formation is estimated to 0.4 Ma or 0.8 to 1.6 Ma [135, 136] and paleolimnological data provide evidence for a nearly complete or even complete desiccation of LV during the late Pleistocene (e.g. [135, 137]). These findings fostered doubts whether the LVRS (following Verheyen et al. [96] and Meier et al. [26]) originated before or after the late Pleistocene desiccation events. Divergence estimates of previous studies ranged between 0.1 Ma and 4.42 Ma, i.e. suggesting that the LVRS origin predates the desiccation of LV [96, 100, 138]. Our mitochondrial divergence time estimate dates the LVRS to around 0.31 Ma (95% HPD: 0.12–0.53 Ma) which is roughly compatible with the estimated age of LV; however, our taxon sampling is not fully representative for that flock as several lineages e.g. from Lake Kivu, Lake Edward and Lake Albert are missing. Nevertheless, it still includes *Haplochromis stappersii*, which together with *Haplochromis* sp. “Yaekama” forms the sister clade to the LVRS [26]. We estimated the divergence age for the MRCA of the LVRS and *H*. *stappersii* to around 0.99 Ma (95% HPD: 0.51–1.53 Ma). Moreover, it has recently been shown by Meier et al. [26] that the LVRS might be the result of ancient hybridization events between two haplochromine lineages (a ‘Congolese lineage’ including for example *H*. *stappersii*, and an Upper Nile lineage consisting of ‘*Haplochromis*’ *gracilior* and *Haplochromis pharyngalis*) which should be considered for the divergence time reconstruction of the LVRS.

Through the inclusion of the newly discovered fossil †*Tugenchromis* and the careful selection of additional calibration points, we provide novel and refined divergence age estimates for most haplochromine radiations. These estimates are still preliminary, however, as for a more accurate reconstruction of the evolutionary history, particularly of the younger haplochromine lineages, additional nuclear DNA-data, younger calibration points and additional analysis methods based on population-level sampling, are needed.

## Conclusion

Our study, based on an alignment of ten mitochondrial protein-coding genes including representative taxa of all cichlid subfamilies, resulted in a comparatively well-resolved mitochondrial phylogenetic hypothesis for cichlids with focus on members of the East African radiation. Bayesian divergence time estimates based on eighteen different calibration sets evaluating even extremely young or old age previous age estimates are, nevertheless highly consistent and several novel mtDNA haplotype lineages are recognized. One is a novel third clade of lower Congo *Lamprologus*, and the other two east-central African ones with considerable phylogeographic interest, i.e., ‘*Orthochromis*’ *indermauri* and *Haplochromis vanheusdeni.* Remarkably, all three novel lineages represent riverine taxa with close affinities to important cichlid radiations. This underscores the importance of a fully representative riverine taxon sampling when phylogenetically inferring the evolutionary history and biogeography of cichlid radiations (e.g. [15, 26, 37, 131]).

Although our study is based to a large part on the protein coding genes of the mitochondrial genome we were able to obtain robust minimum ages of divergence ages associated with the origin of the East African cichlid fauna. Moreover, our molecular clock analysis adds addtitional support to several previously ambiguously supported findings. First, divergence age estimates for the MRCA of the African Pseudocrenilabrinae and Neotropical Cichlinae are consilient with the those of teleost-based Matschiner et al. [9], tentatively supporting the dispersal hypothesis, i.e. that seemingly vicariant phylogeography of Cichlidae can be explained by short-distance marine dispersal events (e.g. [7, 9, 63]), but not with long-distance oceanic dispersal. In particular, the sister relationship of African Pseudocrenilabrinae and Neotropical Cichlinae can be explained with an ecologically plausible dispersal scenario covering only short distances across now submerged island chains between the South American and African continents, e.g. the Rio Grande Rise and the Walvis Ridge.

Further, Genner et al.´s [5] “Ancient Reservoir” and the “Melting Pot Tanganyika” hypothesis of [17] are supported by our cichlid age estimates in combination with the recent discovery of a Miocene EAR cichlid fossil in Kenya exhibiting synapomorphies with several extant Lake Tanganyika cichlids [28], and with recent evidence for repeated hybridization among ancient cichlid lineages in Lake Tanganyika [17, 119]. Our divergence time estimates for almost each of the MRCA of all endemic LT tribes predate the estimated origin of the extant LT basin at 5.5 Ma and only Perissidini and Eretmodini might have formed after the formation of LT.

## Additional files

Additional file 1: **Table S1.** Overview of the taxon sampling with corresponding Genbank accession numbers and, where applicable, corresponding repository numbers of specimens and their origin. (DOCX 43 kb)

Additional file 2: **Table S3.** Overview of the taxon sampling for the nuclear markers (RAG1, ENC1, Rh1 and ttna TMO) with corresponding Genbank accession numbers. (DOCX 75 kb)

Additional file 3: Justification for exclusion of several cichlid fossils from calibration. (DOCX 24 kb)

Additional file 4: **Table S2.** Comprehensive list of mean divergence ages and their corresponding 95% HPD age ranges of selected nodes. (Node numbers 1 to 65 correspond to numbers depicted in Fig. 2). (DOCX 32 kb)

## Abbreviations

BPP: Bayesian Posterior Probability
BS: Bootstrap support
EAR: East African Radiation
HPD: Highest posterior density
LM: Lake Malawi
LT: Tanganyika
LV: Lake Victoria
LVRS: Lake Victoria Region Superflock
ML: Maximum likelihood
MRCA: Most recent common ancestor

## Footnotes

1 There seems to be some confusion with the identification of one or more “*L. teugelsi”* samples in previous studies. After the description of *Lamprologus teugelsi* by Schelly et al. [124] samples and sequences previously identified as *L. mocquardi* (e.g. [90]) were renamed as *L. teugelsi* in subsequent studies (e.g. [76, 120]). Unfortunately, no precise sample location for these samples were provided. With the exception of a dubious type locality record in the primary description, *L. teugelsi* is known only from the Inga area of the Lower Congo rapids. Without locality information and reexamination of those specimens it is not possible to clarify whether *L. teugelsi* carries two different mitochondrial haplotypes or if previously analyzed specimens were misidentified.

## References

[1] Kocher TD (2004). Adaptive evolution and explosive speciation: the cichlid fish model. Nat Rev Genet.

[2] Wagner CE, Harmon LJ, Seehausen O (2012). Ecological opportunity and sexual selection together predict adaptive radiation. Nature.

[3] Santos ME, Braasch I, Boileau N, Meyer BS, Sauteur L, Bohne A, Belting HG, Affolter M, Salzburger W (2014). The evolution of cichlid fish egg-spots is linked with a cis-regulatory change. Nat Commun.

[4] McGee MD, Faircloth BC, Borstein SR, Zheng J, Darrin Hulsey C, Wainwright PC, Alfaro ME (2016). Replicated divergence in cichlid radiations mirrors a major vertebrate innovation. Proc R Soc B Biol Sci.

[5] Genner MJ, Seehausen O, Lunt DH, Joyce DA, Shaw PW, Carvalho GR, Turner GF (2007). Age of cichlids: new dates for ancient lake fish radiations. Mol Biol Evol.

[6] Azuma Y, Kumazawa Y, Miya M, Mabuchi K, Nishida M (2008). Mitogenomic evaluation of the historical biogeography of cichlids toward reliable dating of teleostean divergences. BMC Evol Biol.

[7] Friedman M, Keck BP, Dornburg A, Eytan RI, Martin CH, Hulsey CD, Wainwright PC, Near TJ (2013). Molecular and fossil evidence place the origin of cichlid fishes long after Gondwanan rifting. Proc R Soc B Biol Sci.

[8] McMahan CD, Chakrabarty P, Sparks JS, Smith WM, Davis MP (2013). Temporal patterns of diversification across global cichlid biodiversity (Acanthomorpha: Cichlidae). PLoS One.

[9] Matschiner M, Musilová Z, Barth JMI, Starostova Z, Salzburger W, Steel M, Bouckaert R (2016). Bayesian node dating based on probabilities of fossil sampling supports trans-Atlantic dispersal of cichlid fishes. Syst Biol.

[10] Murray AM (2000). Eocene cichlid fishes from Tanzania, East Africa. J Vertebr Paleontol.

[11] Vences M, Freyhof J, Sonnenberg R, Kosuch J, Veith M (2001). Reconciling fossils and molecules: Cenozoic divergence of cichlid fishes and the biogeography of Madagascar. J Biogeogr.

[12] Sparks JS, Smith WL (2004). Phylogeny and biogeography of cichlid fishes (Teleostei: Perciformes: Cichlidae). Cladistics.

[13] López-Fernandez H, Arbour JH, Winemiller KO, Honeycutt RL (2013). Testing for ancient adaptive radiations in neotropical cichlid fishes. Evolution.

[14] Day JJ, Cotton JA, Barraclough TG. Tempo and mode of diversification of Lake Tanganyika cichlid fishes. PLoS One. 2008:3e1730. 10.1371/journal.pone.0001730 PMC2248707 18320049

[15] Koblmüller S, Schliewen UK, Duftner N, Sefc KM, Katongo C, Sturmbauer C (2008). Age and spread of the haplochromine cichlid fishes in Africa. Mol Phylogenet Evol.

[16] Sturmbauer C, Baric S, Salzburger W, Rüber L, Verheyen E (2001). Lake level fluctuations synchronize genetic divergences of cichlid fishes in African Lakes. Mol Biol Evol.

[17] Weiss JD, Cotterill FP, Schliewen UK (2015). Lake Tanganyika--a ‘melting pot’ of ancient and young cichlid lineages (Teleostei: Cichlidae)?. PLoS One.

[18] Cohen AS, Soreghan MJ, Scholz CA (1993). Estimating the age of formation of lakes: an example from Lake Tanganyika, east African rift system. Geology.

[19] Macgregor D (2015). History of the development of the east African rift system: a series of interpreted maps through time. J Afr Earth Sci.

[20] Bauer FU, Glasmacher UA, Ring U, Schumann A, Nagudi B (2010). Thermal and exhumation history of the central Rwenzori Mountains, Western rift of the east African rift system, Uganda. Int J Earth Sci.

[21] Lezzar KE, Tiercelin JJ, Le Turdu C, Cohen AS, Reynolds DJ, Le Gall B (2002). C.A. S. Control of normal fault interaction on the distribution of major Neogene sedimentary depocenters, Lake Tanganyika, East African rift. AAPG Bull.

[22] Roller S, Hornung J, Hinderer M, Ssemmanda I (2010). Middle Miocene to Pleistocene sedimentary record of rift evolution in the southern Albert rift (Uganda). Int J Earth Sci.

[23] Spiegel C, Kohn BP, Belton DX, Gleadow AJW (2007). Morphotectonic evolution of the Central Kenya rift flanks: implications for late Cenozoic environmental change in East Africa. Geology.

[24] Delvaux D. Age of Lake Malawi (Nyasa) and water level fluctuations. Mus roy Afr cent, Tervuren (Belg), Dépt Géol Min. 1995;1995-1996:99–108.

[25] Lyons RP, Scholz CA, Cohen AS, King JW, Brown ET, Ivory SJ, Johnson TC, Deino AL, Reinthal PN, McGlue MM (2015). Continuous 1.3-million-year record of east African hydroclimate, and implications for patterns of evolution and biodiversity. Proc Natl Acad Sci U S A.

[26] Meier JI, Marques DA, Mwaiko S, Wagner CE, Excoffier L, Seehausen O (2017). Ancient hybridization fuels rapid cichlid fish adaptive radiations. Nat Commun.

[27] Cotterill FPD, De Wit MJ (2011). Geoecodynamics and the Kalahari epeirogeny: linking its genomic record, tree of life and palimpsest into a unified narrative of landscape evolution. S Afr J Geol.

[28] Altner M, Schliewen UK, Penk SBR, Reichenbacher B (2017). †Tugenchromis pickfordi, gen. Et sp. nov., from the upper Miocene—a stem-group cichlid of the ‘east African radiation’. J Vertebr Paleontol.

[29] Matschiner Michael (2018). Gondwanan vicariance or trans-Atlantic dispersal of cichlid fishes: a review of the molecular evidence. Hydrobiologia.

[30] Takahashi T (2003). Systematics of Tanganyikan cichlid fishes (Teleostei: Perciformes). Ichthyol Res.

[31] Koblmüller S, Sefc KM, Sturmbauer C (2008). The Lake Tanganyika cichlid species assemblage: recent advances in molecular phylogenetics. Hydrobiologia.

[32] Greenwood PH. Towards a phyletic classification of the `genus´ Haplochromis (Pisces, Cichlidae) and related taxa. Bull British Museum (Natural History) Zool. 1979;35:265–322.

[33] Hoogerhoud RJC (1983). A taxonomic reconsideration of the haplochromine genera Gaurochromis Greenwood, 1980 and Labrochromis Regan, 1920 (Pisces, Cichlidae). Netherlands J Zool.

[34] Schwarzer J, Swartz ER, Vreven E, Snoeks J, Cotterill FP, Misof B, Schliewen UK (2012). Repeated trans-watershed hybridization among haplochromine cichlids (Cichlidae) was triggered by Neogene landscape evolution. Proc R Soc B Biol Sci.

[35] Schwarzer J, Misof B, Tautz D, Schliewen UK (2009). The root of the east African cichlid radiations. BMC Evol Biol.

[36] Dunz AR, Schliewen UK (2013). Molecular phylogeny and revised classification of the haplotilapiine cichlid fishes formerly referred to as “*Tilapia*”. Mol Phylogenet Evol.

[37] Salzburger W, Mack T, Verheyen E, Meyer A (2005). Out of Tanganyika: genesis, explosive speciation, key-innovations and phylogeography of the haplochromine cichlid fishes. BMC Evol Biol.

[38] Koblmüller S, Duftner N, Katongo C, Phiri H, Sturmbauer C (2005). Ancient divergence in bathypelagic lake tanganyika Deepwater cichlids: mitochondrial phylogeny of the tribe bathybatini. J Mol Evol.

[39] Musilová Z, Kalous L, Petrtyl M, Chaloupkova P (2013). Cichlid fishes in the Angolan headwaters region: molecular evidence of the ichthyofaunal contact between the Cuanza and Okavango-Zambezi systems. PLoS One.

[40] Neumann D (2010). Preservation of freshwater fishes in the field. Abc Taxa.

[41] Kawaguchi A, Miya M, Nishida M (2001). Complete mitochondrial DNA sequence of Aulopus japonicas (Telostei: Aulopiformes), a basal Eurypterygii: longer DNA sequences and higher-level relationships. Ichthyol Res.

[42] Kearse M, Moir R, Wilson A, Stones-Havas S, Cheung M, Sturrock S, Buxton S, Cooper A, Markowitz S, Duran C (2012). Geneious basic: an integrated and extendable desktop software platform for the organization and analysis of sequence data. Bioinformatics.

[43] He A, Luo Y, Yang H, Liu L, Li S, Wang C (2011). Complete mitochondrial DNA sequences of the Nile tilapia (Oreochromis niloticus) and blue tilapia (Oreochromis aureus): genome characterization and phylogeny applications. Mol Biol Rep.

[44] Swofford D (2003). PAUP*: phylogenetic analysis using parsimony.

[45] Posada D (2008). jModelTest: Phylogenetic Model Averaging. Mol Phylogenet Evol.

[46] Stamatakis Alexandros (2014). RAxML version 8: a tool for phylogenetic analysis and post-analysis of large phylogenies. Bioinformatics.

[47] Miller MA, Pfeiffer W, Schwartz T (2010). Creating the CIPRES Science Gateway for inference of large phylogenetic trees.

[48] Drummond AJ, Rambaut A (2007). BEAST: Bayesian evolutionary analysis by sampling trees. BMC Evol Biol.

[49] Perez GA, Rican O, Orti G, Bermingham E, Doadrio I, Zardoya R (2007). Phylogeny and biogeography of 91 species of heroine cichlids (Teleostei: Cichlidae) based on sequences of the cytochrome b gene. Mol Phylogenet Evol.

[50] Malabarba MC, Malabarba LR, del Papa C (2010). Gymnogeophagus eocenicus (Perciformes: Cichlidae), an Eocene cichlid from the Lumbrera formation in Argentina. J Vertebr Paleontol.

[51] del Papa C, Kirschbaum A, Powell J, Brod A, Hongn F, Pimentel M (2010). Sedimentological, geochemical and paleontological insights applied to continental omission surfaces: a new approach for reconstructing an eocene foreland basin in NW Argentina. J S Am Earth Sci.

[52] Musilová Z, Říčan O, Říčanová S, Janšta P, Gahura O, Novák J (2015). Phylogeny and historical biogeography of trans-Andean cichlid fishes (Teleostei: Cichlidae). Vertebrate Zool.

[53] Perez PA, Malabarba MC, del Papa C (2010). A new genus and species of Heroini (Perciformes : Cichlidae) from the early Eocene of southern South America. Neotropical Ichthyology.

[54] López-Fernandez H, Winemiller KO, Honeycutt RL (2010). Multilocus phylogeny and rapid radiations in Neotropical cichlid fishes (Perciformes: Cichlidae: Cichlinae). Mol Phylogenet Evol.

[55] Malabarba MC, Malabarba LR (2008). A new cichlid, Tremembichthys garciae (Actinopterygii, Perciformes) from the Eocene - Oligocene of eastern Brazil. Revista Brasileira de Paleontologia.

[56] Oliveira MEB, Garcia MJ, Fernandes MCC (2006). Folíolose grãos de pólen de Fabales na Formação Entre-Córregos, Paleógeno da bacia de Aiuruoca, sudeste de Minas Gerais, Brasil.

[57] Garcia MJ, Santos M, Hasui Y (2000). Palinologia da parte aflorante da Formação Entre-Córregos, Bacia de Aiuruoca, Terciário do Estado de Minas Gerais, Brasil. Revista Universidade Guarulhos.

[58] Lima MR, Salard-Cheboldaeff M, Suguio K, CSF DAC, Brito IM, C F (1985). Étude palynologique de la Formation Tremembé, Tertiaire du Bassin de Taubaté (État de São Paulo, Brasil), d’aprés lês echantillons du sondage n-42 du CNP. *Coletânea de Trabalhos Paleontológicos*.

[59] Riccomini C, Sant’Anna LG, Ferrari AL. Evolução geológica do rift Continental do Sudeste do Brasil. In: Mantesso-Neto V, Bartorelli A, Carneiro CDR, Brito Neves BB, editors*. Geologia do continente Sul-Americano: evolução da obra de Fernando Flávio de Almeida.* SãoPaulo: Beca; 2004. p. 383–406.

[60] Kullander SO. A phylogeny and classification of the South American Cichlid (Teleostei:Perciformes). In: Malabarba LR, Reis R, Vari RP, Lucena ZM, Lucena CA, editors. *Phylogeny and Classification of Neotropical Fishes*. Porto Alegre: Edipucrs; 1998. p. 461–498.

[61] López-Fernandez H, Honeycutt RL, Stiassny MLJ, Winemiller KO (2005). Morphology, molecules, and character congruence in the phylogeny of south American geophagine cichlids (Perciformes, Labroidei). Zool Scr.

[62] Říčan O, Piálek L, Zardoya R, Doadrio I, Zrzavý J, Crame A (2013). Biogeography of the Mesoamerican Cichlidae (Teleostei: Heroini): colonization through the GAARlandia land bridge and early diversification. J Biogeogr.

[63] Murray AM (2001). The fossil record and biogeography of the Cichlidae (Actinopterygii: Labroidei). Biol J Linn Soc.

[64] Harrison T, Msuya CP, Murray AM, Fine Jacobs B, Báez AM, Mundil R, Ludwig KR, Gunnel GF (2001). Paleontological investigations at the Eocene locality of Mahenge in north-Central Tanzania, East Africa. *Eocene biodiversity: unusual occurrences and rarely sampled habitats*.

[65] Murray AM (2000). The Eocene cichlids (Perciformes: Labroidei) of Mahenge, Tanzania.

[66] Greenwood PH (1987). The genera of pelmatochromine fishes (Teleostei, Cichlidae). A phylogenetic review. Bull British Museum (Natural History) Zool.

[67] Schliewen UK, Stiassny ML (2003). Etia nguti, a new genus and species of cichlid fish from the river Mamfue, upper Cross River basin in Cameroon, West-Central Africa. Ichthyological Explor Freshwaters.

[68] Carnevale GLW, Sorbini C (2003). Oreochromis lorenzoi, a new species of tilapiine cichlid from the late Miocene of Central Italy. J Vertebr Paleontol.

[69] Hilgen FJ, Krijgsman W, Langereis CG, Lourens LJ, Santarelli A, Zachariasse WJ (1995). Extending the astronomical (polarity) time scale into the Miocene: earth planet. Earth Planet Sci Lett.

[70] Krijgsman W, Hilgen FJ, Marabini S, Vai GB (1999). New palaeomagnetic and cyclostratigraphic age constraints on the Messinian of the northern Apennines (vena del Gesso Basin, Italy). Mem Soc Geol Ital.

[71] Krijgsman W, Hilgen FJ, Raffi I, Sierro FJ, Wilson DS (1999). Chronology, causes and progression of the Messinian salinity crisis. Letters to Nature.

[72] Trewavas E (1983). Tilapiine fishes of the genera Sarotherodon, Oreochromis and Danakilia.

[73] Pickford MHL (1978). Geology, palaeoenvironments and vertebrate faunas of the mid- Miocene Ngoroa formation, Kenya. Geol Soc Lond, Spec Publ.

[74] Rasmussen C, Reichenbacher B, Lenz O, Altner M, Penk SBR, Prieto J, BrÜSch D (2015). Middle–late Miocene palaeoenvironments, palynological data and a fossil fish Lagerstätte from the Central Kenya rift (East Africa). Geol Mag.

[75] Fine Jacobs Bonnie (2002). Estimation of low-latitude paleoclimates using fossil angiosperm leaves: examples from the Miocene Tugen Hills, Kenya. Paleobiology.

[76] Clabaut C, Salzburger W, Meyer A (2005). Comparative phylogenetic analyses of the adaptive radiation of Lake Tanganyika cichlid fish: nuclear sequences are less homoplasious but also less informative than mitochondrial DNA. J Mol Ecol.

[77] Nishida M (1991). Lake Tanganyika as an evolutionary reservoir of old lineages of east African cichlid fishes: inferences from allozyme data. Experientia.

[78] Schliewen UK, Klee B (2004). Reticulate sympatric speciation in Cameroonian crater lake cichlids. Front Zool.

[79] Schliewen UK, Tautz D, Pääbo S (1994). Sympatric speciation suggested by monophyly of crater lake cichlids. Lett Nature.

[80] Cornen G, Bandet Y, Giresse P, Maley J (1992). The nature and chronostratigraphy of quaternary pyroplastic accumulations from Lake Barombi Mbo (West-Cameroon). J Volcanol Geotherm Res.

[81] Phillips MJ (2009). Branch-length estimation bias misleads molecular dating for a vertebrate mitochondrial phylogeny. Gene.

[82] Lukoschek V, Scott Keogh J, Avise JC (2012). Evaluating fossil calibrations for dating phylogenies in light of rates of molecular evolution: a comparison of three approaches. Syst Biol.

[83] Hugall AF, Lee MS (2004). Molecular claims of Gondwanan age for Australian agamid lizards are untenable. Mol Biol Evol.

[84] Rambaut A, Suchard MA, Xie D, Drummond AJ. Tracer v1.6, Available from http://beast.bio.ed.ac.uk/Tracer. 2014.

[85] Duchêne S, Lanfear R, Ho SY (2014). The impact of calibration and clock-model choice on molecular estimates of divergence times. Mol Biol Evol.

[86] Ho SY (2014). The changing face of the molecular evolutionary clock. Trends Ecol Evol.

[87] Ho SY, Larson G (2006). Molecular clocks: when timesare a-changin’. Trends Genet.

[88] Ho SY, Lanfear R, Bromham L, Phillips MJ, Soubrier J, Rodrigo AG, Cooper A (2011). Time-dependent rates of molecular evolution. Mol Ecol.

[89] Schwarzer J, Lamboj A, Langen K, Misof B, Schliewen UK (2014). Phylogeny and age of chromidotilapiine cichlids (Teleostei: Cichlidae). Hydrobiologia.

[90] Salzburger W, Meyer A, Baric S, Verheyen E, Sturmbauer C (2002). Phylogeny of the Lake Tanganyika cichlid species flock and its relationship to the central and east African haplochromine cichlid fish faunas. Syst Biol.

[91] Kocher TD, Conroy JA, McKaye KR, Stauffer JR, Lockwood SF (1995). Evolution of NADH dehydrogenase subunit 2 in east African cichlid fish. Mol Phylogenet Evol.

[92] Duftner N, Koblmuller S, Sturmbauer C (2005). Evolutionary relationships of the limnochromini, a tribe of benthic Deepwater cichlid fish endemic to Lake Tanganyika, East Africa. J Mol Evol.

[93] Takahashi T, Sota T (2016). A robust phylogeny among major lineages of the east African cichlids. Mol Phylogenet Evol.

[94] Mayer WE, Tichy H, Klein J (1998). Phylogeny of African cichlid fishes as revealed by molecular markers. Heredity.

[95] Joyce DA, Lunt DH, Bills R, Turner GF, Katongo C, Duftner N, Sturmbauer C, Seehausen O (2005). An extant cichlid fish radiation emerged in an extinct Pleistocene lake. Nature.

[96] Verheyen E, Salzburger W, Snoeks J, Meyer A (2003). Origin of the superflock of cichlid fishes from Lake Victoria, East Africa. Science.

[97] Schedel FDB, Katemo Manda B, Chocha Manda A, Abwe E, Vreven EJWMN, Schliewen UK (2018). Description of five new rheophilic Orthochromis species (Teleostei: Cichlidae) from the upper Congo drainage in Zambia and the Democratic Republic of the Congo. Zootaxa.

[98] Cummings MP, Handley SA, Myers DS, Reed DL, Rokas A, Winka K, Rannala B (2003). Comparing bootstrap and posterior probability values in the four-taxon case. Syst Biol.

[99] Erixon P, Svennblad B, Britton T, Oxelman B, Sullivan J (2003). Reliability of Bayesian posterior probabilities and bootstrap frequencies in Phylogenetics. Syst Biol.

[100] Meyer BS, Matschiner M, Salzburger W. Disentangling incomplete lineage sorting and introgression to refine species-tree estimates for Lake Tanganyika cichlid fishes. Systematic Biol. 2016;66:531–550. 10.1093/sysbio/syw069 27539485

[101] Irisarri I, Singh P, Koblmuller S, Torres-Dowdall J, Henning F, Franchini P, Fischer C, Lemmon AR, Lemmon EM, Thallinger GG (2018). Phylogenomics uncovers early hybridization and adaptive loci shaping the radiation of Lake Tanganyika cichlid fishes. Nat Commun.

[102] Říčan O, Piálek P, Dragová K, Novák J (2016). Diversity and evolution of the middle American cichlid fishes (Teleostei: Cichlidae) with revised classification. Vertebrate Zool.

[103] Malabarba MC, Zuleta OD, del Papa C (2006). Proterocara Argentina, a new fossil cichlid from the Lumbrera formation, Eocene of Argentina. J Vertebr Paleontol.

[104] Smith WL, Chakrabarty P, Sparks JS (2008). Phylogeny, taxonomy, and evolution of Neotropical cichlids (Teleostei: Cichlidae: Cichlinae). Cladistics.

[105] Murray AM (2004). Late Eocene and early Oligocene teleost and associated ichthyofauna of the Jebel Qatrani formation, Fayum, Egypt. Palaeontology.

[106] Lippitsch E, Micklich N (1998). Cichlid fish biodiversity in an Oligocene lake. Ital J Zool.

[107] Tamura K, Battistuzzi FU, Billing-Ross P, Murillo O, Filipski A, Kumar S (2012). Estimating divergence times in large molecular phylogenies. Proc Natl Acad Sci.

[108] Lozano-Fernandez J, Dos Reis M, Donoghue PCJ, Pisani D (2017). RelTime rates collapse to a strict clock when estimating the timeline of animal diversification. Genome Biol Evol.

[109] Battistuzzi FU, Tao Q, Jones L, Tamura K, Kumar S (2018). RelTime relaxes the strict molecular clock throughout the phylogeny. Genome Biol Evol.

[110] Chakrabarty P (2004). Cichlid biogeography: comment and review. Fish Fish.

[111] Heine C, Zoethout J, Müller RD (2013). Kinematics of the South Atlantic rift. Solid Earth.

[112] Measey GJ, Vences M, Drewes RC, Chiari Y, Melo M, Bourles B (2007). Freshwater paths across the ocean: molecular phylogeny of the frog Ptychadena newtoni gives insights into amphibian colonization of oceanic islands. J Biogeogr.

[113] de Oliveira FB, Molina EC, Marroig G. Paleogeography of the South Atlantic: a route for Primates and rodents into the New World? In: Garber PA, Estrada A, Bicca-Marques JC, Heymann EW, editors. *South American Primates, developments in Primatology: Progress and Prospects. * New York: Springer; 2009. p. 55–68.

[114] Markwick PJ, Valdes PJ (2004). Palaeo-digital elevation models for use as boundary conditions in coupled ocean–atmosphere GCM experiments: a Maastrichtian (late cretaceous) example. Palaeogeogr Palaeoclimatol Palaeoecol.

[115] Reinthal PN, Stiassny MLJ (1991). The freshwater fishes of Madagascar: a study of an endangered fauna with recomnlendations for a conservation strategy. Conserv Biol.

[116] Ward JA, Wyman RL (1977). Ethology and ecology of cichlid fishes of the genus Etroplus in Sri Lanka: preliminary findings. Environ Biol Fish.

[117] Uchida K, Kaneko T, Miyazaki H, Hasegawa S, Hirano T (2000). Excellent salinity tolerance of Mozambique Tilapia (Oreochromis mossambicus): elevated chloride cell activity in the branchial and Opercular epithelia of the fish adapted to concentrated seawater. Zool Sci.

[118] Tiercelin JJ, Mondeguer A. The geology of the Tanganyika trough. In: Coulter GW, editor. *Lake Tanganyika and its Life*. London: Oxford University Press; 1991. p. 7–48.

[119] Meyer BS, Matschiner M, Salzburger W (2015). A tribal level phylogeny of Lake Tanganyika cichlid fishes based on a genomic multi-marker approach. Mol Phylogenet Evol.

[120] Day JJ, Santini S, Garcia-Moreno J (2007). Phylogenetic relationships of the Lake Tanganyika cichlid tribe Lamprologini: the story from mitochondrial DNA. Mol Phylogenet Evol.

[121] Sturmbauer CE, Verheyen E, Meyer A (1994). Mitochondrial phylogeny of the Lamprologini, the major substrate spawning lineage of cichlid fish from Lake Tanganyika in eastern Africa. Mol Phylogenet Evol.

[122] Sturmbauer C, Salzburger W, Duftner N, Schelly R, Koblmuller S (2010). Evolutionary history of the Lake Tanganyika cichlid tribe Lamprologini (Teleostei: Perciformes) derived from mitochondrial and nuclear DNA data. Mol Phylogenet Evol.

[123] Schwarzer J, Misof B, Ifuta SN, Schliewen UK (2011). Time and origin of cichlid colonization of the lower Congo rapids. PLoS One.

[124] Schelly RC, Stiassny MLJ (2004). Revision of the Congo river *Lamprologus* Schilthuis, 1891 (Teleostei: Cichlidae), with description of two new species. Am Mus Novit.

[125] Schelly R, Salzburger W, Koblmuller S, Duftner N, Sturmbauer C (2006). Phylogenetic relationships of the lamprologine cichlid genus Lepidiolamprologus (Teleostei: Perciformes) based on mitochondrial and nuclear sequences, suggesting introgressive hybridization. Mol Phylogenet Evol.

[126] Nevado B, Koblmuller S, Sturmbauer C, Snoeks J, Usano-Alemany J, Verheyen E (2009). Complete mitochondrial DNA replacement in a Lake Tanganyika cichlid fish. Mol Ecol.

[127] Sturmbauer C, Koblmüller S, Sefc KM, Duftner N (2005). Phylogeographic history of the genus Tropheus, a lineage of rock-dwelling cichlid fishes endemic to Lake Tanganyika. Hydrobiologia.

[128] Koblmüller S, Egger B, Sturmbauer C, Sefc KM (2010). Rapid radiation, ancient incomplete lineage sorting and ancient hybridization in the endemic Lake Tanganyika cichlid tribe Tropheini. Mol Phylogenet Evol.

[129] Sturmbauer C, Börger C, Van Steenberge M, Koblmüller S (2016). A separate lowstand lake at the northern edge of Lake Tanganyika? Evidence from phylogeographic patterns in the cichlid genus Tropheus. Hydrobiologia.

[130] Genner MJ, Ngatunga BP, Mzighani S, Smith A, Turner GF (2015). Geographical ancestry of Lake Malawi’s cichlid fish diversity. Biol Lett.

[131] Genner MJ, Turner GF (2014). Timing of population expansions within the Lake Malawi haplochromine cichlid fish radiation. Hydrobiologia.

[132] Ebinger CJ, Deino AL, Drake RE, Tesha AL (1989). Chronology of volcanism and rift basin propagation: Rungwe Volcanic Province, East Africa. Geol Soc Am Bull.

[133] Ebinger CJ, Deino AL, Tesha AL, Becker T, Ring U (1993). Tectonic controls on rift basin morphology: evolution of the northern Malawi (Nyasa) rift. J Geophys Res.

[134] Betzler C, Ring U (1995). Edimentology of the Malawi rift: facies and stratigraphy oft he Chiwondo beds, northern Malawi. J Hum Evol.

[135] Johnson TC, Scholz CA, Talbot MR, Kelts K, Ricketts RD, Ngobi G, Beuning K, Ssemmanda I, McGill JW (1996). Late Pleistocene desiccation of Lake Victoria and rapid evolution of cichlid fishes. Science.

[136] Kent PE (1944). The Miocene beds of Kavirondo, Kenya. Q J Geol Soc.

[137] Stager JC, Johnson TC (2007). The late Pleistocene desiccation of Lake Victoria and the origin of its endemic biota. Hydrobiologia.

[138] Elmer KR, Reggio C, Wirth T, Verheyen E, Salzburger W, Meyer A (2009). Pleistocene desiccation in East Africa bottlenecked but did not extirpate the adaptive radiation of Lake Victoria haplochromine cichlid fishes. PNAS.

