# Supplementary material for: East African cichlid lineages (Teleostei: Cichlidae) might be older than their ancient host lakes: new divergence estimates for the east African cichlid radiation
Source: BMC Evol Biol. 2019 Apr 25;19:94. doi: 10.1186/s12862-019-1417-0 (PMC6482553; doi:10.1186/s12862-019-1417-0)
Supplement: Supplementary file 1 — Table S1. Overview of the taxon sampling with corresponding Genbank accession numbers and, where applicable, corresponding repository numbers of specimens and their origin. (DOCX 43 kb) [file 12862_2019_1417_MOESM1_ESM.docx]

**SchedelEtal_Table_A1**

Table A.1

Overview of the taxon sampling with corresponding Genbank accession numbers and, where applicable, corresponding repository numbers of specimens and their origin.

Table A.1: Overiew of the taxon sampling with corresponding Genbank accession numbers.(Abbreviations: AS = Aquarium specimen, GB= Genbank, TS= This study, NI= No further locality information available)

| **Subfamily** | **Tribe/Group names used in this study** | **Genus** | **Species** | **Autor** | **ZSM tissue No. / DNA Field number** | **ZSM collection number/or other repository number** | **Sampling location** | **Missing data:** | **Sequence from** | **Genbank accesion no.** |
| --- | --- | --- | --- | --- | --- | --- | --- | --- | --- | --- |
| Ptychochrominae | Paratilapiinae | *Paratilapia* | *polleni* | Bleeker 1868 | - | - | - | - | GB | NC_011170.1 |
| Ptychochrominae | Ptychochrominae | *Katria* | *katria* | (Reinthal & Stiassny 1997) | - | - | - | - | GB | NC_011169.1 |
| Etroplinae | Etroplinae | *Paretroplus* | *maculatus* | Kiener & Maugé 1966 | - | - | - | - | GB | NC_011177.1 |
| Etroplinae | Etroplinae | *Etroplus* | *maculatus* | (Bloch 1795) | - | - | - | - | GB | NC_011179.1 |
| Cichlinae | Retroculini | *Retroculus* | *lapidifer* | (Castelnau 1855) | - | - | - | - | GB | NC_033549 |
| Cichlinae | Astronotini | *Astronotus* | *ocellatus* | (Agassiz 1831) | - | - | - | - | GB | AP009127.1 |
| Cichlinae | Chaetobranchini | *Chaetobranchopsis* | *bitaeniatus* | Ahl 1936 | - | - | - | - | GB | KR150861.1 |
| Cichlinae | Cichlasomatini | *Andinoacara* | *rivulatus* | (Günther 1860) | - | - | - | - | GB | NC_025671.1 |
| Cichlinae | Cichlasomatini | *Nannacara* | *anomala* | Regan 1905 | - | - | - | - | GB | NC_031183 |
| Cichlinae | Cichlasomatini | *Aequidens* | *metae* | Eigenmann 1922 | - | - | - | - | GB | KR150865 |
| Cichlinae | Cichlasomatini | *Andinoacara* | *pulcher* | (Gill 1858) | - | - | - | - | GB | NC_033547 |
| Cichlinae | Cichlasomatini | *Bujurquina* | *mariae* | (Eigenmann 1922) | - | - | - | - | GB | KR150862 |
| Cichlinae | Cichlasomatini | *Cichlasoma* | *dimerus* | (Heckel 1840) | - | - | - | - | GB | NC_033551 |
| Cichlinae | Cichlasomatini | *Krobia* | *guianensis* | (Regan, 1905) | - | - | - | - | GB | NC_031440 |
| Cichlinae | Cichlasomatini | *Laetacara* | *thayeri* | (Steindachner 1875) | - | - | - | - | GB | KR233974 |
| Cichlinae | Heroini | *Paraneetroplus* | *synspilus* | (Hubbs 1935) | - | - | - | - | GB | NC_023526.1 |
| Cichlinae | Heroini | *Hypselecara* | *temporalis* | (Günther 1862) | - | - | - | - | GB | NC_011168.1 |
| Cichlinae | Heroini | *Petenia* | *splendida* | Günther 1862 | - | - | - | - | GB | NC_024835.1 |
| Cichlinae | Heroini | *Pterophyllum* | *scalare* | (Schultze 1823) | - | - | - | - | GB | NC_026535.1 |
| Cichlinae | Heroini | *Pterophyllum* | *altum* | Pellegrin 1903 | - | - | - | - | GB | KT180164 |
| Cichlinae | Heroini | *Symphysodon* | *haraldi* | Schultz 1960 | - | - | - | - | GB | NC_027965 |
| Cichlinae | Heroini | *Symphysodon* | *discus* | Heckel 1840 | - | - | - | - | GB | NC_026689 |
| Cichlinae | Heroini | *Symphysodon* | *aequifasciata* | Pellegrin 1904 | - | - | - | - | GB | KT362183 |
| Cichlinae | Heroini | *Parachromis* | *managuensis* | (Günther 1867) | - | - | - | - | GB | NC_026918 |
| Cichlinae | Heroini | *Cryptoheros* | *cutteri* | (Fowler 1932) | - | - | - | - | GB | KR150878 |
| Cichlinae | Heroini | *Herichthys* | *cyanoguttatus* | Baird & Girard 1854 | - | - | - | - | GB | NC_033546 |
| Cichlinae | Heroini | *Rocio* | *octofasciata* | (Regan 1903) | - | - | - | - | GB | NC_033548 |
| Cichlinae | Heroini | *Thorichthys* | *aureus* | (Günther 1862) | - | - | - | - | GB | NC_031182 |
| Cichlinae | Heroini | *Uaru* | *amphiacanthoides* | Heckel 1840 | - | - | - | - | GB | NC_033550 |
| Cichlinae | Heroini | *Amphilophus* | *citrinellus* | (Günther 1864) | - | - | - | - | GB | KJ562277.1 |
| Cichlinae | Cichlini | *Cichla* | *ocellaris* | Bloch & Schneider 1801 | - | - | - | - | GB | KR150863 |
| Cichlinae | Geophagini | *Mikrogeophagus* | *ramirezi* | (Myers & Harry 1948) | - | - | - | - | GB | NC_031439 |
| Cichlinae | Geophagini | *Geophagus* | *brasiliensis* | (Quoy & Gaimard 1824) | - | - | - | - | GB | NC_031181 |
| Cichlinae | Geophagini | *Geophagus* | *steindachneri* | Eigenmann & Hildebrand 1922 | - | - | - | - | GB | NC_033545 |
| Cichlinae | Geophagini | *Gymnogeophagus* | *balzanii* | (Perugia, 1891) | - | - | - | - | GB | KR150864.1 |
| Pseudocrenilabrinae | Tylochromini | *Tylochromis* | *polylepis* | (Boulenger 1900) | - | - | - | - | GB | NC_011171.1 |
| Pseudocrenilabrinae | Chromidotilapini | *Nanochromis* | *consortus* | Roberts & Stewart, 1976 | DRC-2013/2736 | ZSM-PIS-042667 | Congo river, DRC | - | TS | MK144732 |
| Pseudocrenilabrinae | Chromidotilapini | *Teleogramma* | *depressa* | Roberts & Stewart, 1976 | DRC-2013/1768 | ZSM-PIS-042679 | Congo river, DRC | - | TS | MK144769 |
| Pseudocrenilabrinae | Hemichromini | *Anomalochromis* | *thomasi* | (Boulenger, 1915) | P-AA-1099 | - | AS, NI | - | TS | MK144670 |
| Pseudocrenilabrinae | Hemichromini | *Hemichromis* | *letourneuxi* | Sauvage, 1880 | P-AA-0620 | ZSM-PIS-040946 | AS, (Egypt) | - | TS | MK144717 |
| Pseudocrenilabrinae | Pelmatolapiini | *Pelmatolapia* | *mariae* | (Boulenger, 1899) | B125 | ZSM-PIS-035999 | Iguidi River, Nigeria | - | TS | MK144750 |
| Pseudocrenilabrinae | Gobiocichlini | *Tilapia* | *brevimanus* | Boulenger, 1911 | TI121 | ZSM-PIS-041948 | AS, NI | - | TS | MK144772 |
| Pseudocrenilabrinae | Coptodonini | *Coptodon* | *discolor* | (Günther 1903) | P-AA-1071 | - | AS, (Ghana) | - | TS | MK144688 |
| Pseudocrenilabrinae | Coptodonini | *Coptodon* | *zillii* | (Gervais 1848) | - | - | - | - | GB | NC_026110.1 |
| Pseudocrenilabrinae | Pelmatochromini | *Pelmatochromis* | *buettikoferi* | (Pellegrin, 1900) | P-AA-1017 | ZSM-PIS-043935 | AS, NI | - | TS | MK144749 |
| Pseudocrenilabrinae | Oreochromini | *Stomatepia* | *pindu* | Trewavas 1972 | bar03_pindC26 | - | AS, Lake Barombi-Mbo, Cameroon | - | TS | MK170265 |
| Pseudocrenilabrinae | Oreochromini | *Myaka* | *myaka* | Trewavas 1972 | bar18_myakZ101 | - | AS, Lake Barombi-Mbo, Cameroon | - | TS | MK170262 |
| Pseudocrenilabrinae | Oreochromini | *Sarotherodon* | *lohbergeri* | (Holly, 1930) | bar06_lohbZ011 | - | AS, Lake Barombi-Mbo, Cameroon | - | TS | MK170264 |
| Pseudocrenilabrinae | Oreochromini | *Sarotherodon* | *linnellii* | (Löhnberg, 1903) | bar32_linnAZ05 | - | AS, Lake Barombi-Mbo, Cameroon | - | TS | MK170260 |
| Pseudocrenilabrinae | Oreochromini | *Pungu* | *maclareni* | (Trewavas, 1962) | bar19_pungC25 | - | AS, Lake Barombi-Mbo, Cameroon | - | TS | MK170261 |
| Pseudocrenilabrinae | Oreochromini | *Konia* | *dikume* | Trewavas 1972 | bar07_dikuB06 | - | AS, Lake Barombi-Mbo, Cameroon | - | TS | MK170263 |
| Pseudocrenilabrinae | Oreochromini | *Sarotherodon* | *sanagaensis* | (Linnaeus 1758) | P-AA-1067 | - | AS, (Cameroon) | - | TS | MK144760 |
| Pseudocrenilabrinae | Oreochromini | *Danakilia* | *sp. "Galli Colulli"* | - | P-AA-1059 | - | AS, (Eritrea) | - | TS | MK144693 |
| Pseudocrenilabrinae | Oreochromini | *Sarotherodon* | *mvogoi* | (Thys van den Audenaerde 1965) | P-AA-0600 | ZSM-PIS-040952 | AS, (Dja River, Cameroon) | - | TS | MK144759 |
| Pseudocrenilabrinae | Oreochromini | *Oreochromis* | *cf. urolepis* | (Norman 1922) | DRC-2011/1041 | - | Little Ruaha, Tanzania | - | TS | MK144736 |
| Pseudocrenilabrinae | Oreochromini | *Oreochromis* | *niloticus* | (Linnaeus 1758) | - | - | - | - | GB | GU370126.1 |
| Pseudocrenilabrinae | Oreochromini | *Sarotherodon* | *melanotheron* | Rüppell 1852 | - | - | - | - | GB | NC_015611.1 |
| Pseudocrenilabrinae | Oreochromini | *Oreochromis* | *variabilis* | (Boulenger 1906) | - | - | - | - | GB | NC_026109 |
| Pseudocrenilabrinae | Oreochromini | *Oreochromis* | *esculentus* | (Graham 1928) | - | - | - | - | GB | NC_025555 |
| Pseudocrenilabrinae | Tilapiini | *Tilapia* | *sparrmanii* | Smith 1840 | P-AA-0576 | ZSM-PIS-040917 | AS, (Orange River, South Africa) | COX3 | TS | MK144774 |
| Pseudocrenilabrinae | Tilapiini | *Tilapia* | *ruweti* | (Poll & Thys van den Audenaerde 1965) | P-AA-0578 | ZSM-PIS-040919 | AS, (Katanga, DRC) | - | TS | MK144773 |
| Pseudocrenilabrinae | Tilapiini | *Congolapia* | *crassa* | (Pellegrin 1903) | AMNH 242004 | AMNH 242002 | Luilaka River, DRC | - | TS | MK144686 & MK144687 |
| Pseudocrenilabrinae | Steatocranini | *Steatocranus* | *sp. Redeye* | - | P-AA-0575 | ZSM-PIS-042378 | AS, (Congo River, DRC) | - | TS | MK144767 |
| Pseudocrenilabrinae | Steatocranini | *Steatocranus* | *sp. dwarf* | - | P-AA-1096 | ZSM-PIS-043995 | AS, (Congo River, DRC) | - | TS | MK144768 |
| Pseudocrenilabrinae | Steatocranini | *Steatocranus* | *glaber* | - | P-AA-1123 | - | AS, (Congo River, DRC) MK144730 | - | TS | MK144766 |
| Pseudocrenilabrinae | Boulengerochromis / 'most ancient Tanganyika tribes' | *Boulengerochromis* | *microlepis* | (Boulenger 1899) | P-AA-0502 | ZSM-PIS-040843 | AS, (Lake Tanganyika) | - | TS | MK144682 |
| Pseudocrenilabrinae | Bathybatini / 'most ancient Tanganyika tribes' | *Bathybates* | *vittatus* | Boulenger, 1914 | DRC-2012/3205 | ZSM-PIS-044555 | Lake Tanganyika, Zambia | - | TS | MK144680 |
| Pseudocrenilabrinae | Bathybatini / 'most ancient Tanganyika tribes' | *Bathybates* | *minor* | Boulenger, 1906 | DRC-2012/3188 | ZSM-PIS-044556 | Lake Tanganyika, Zambia | - | TS | MK144679 |
| Pseudocrenilabrinae | Bathybatini / 'most ancient Tanganyika tribes' | *Bathybates* | *leo* | Poll, 1956 | DRC-2012/3193 | ZSM-PIS-044557 | Lake Tanganyika, Zambia | - | TS | MK144675 |
| Pseudocrenilabrinae | Bathybatini / 'most ancient Tanganyika tribes' | *Bathybates* | *graueri* | Steindacher, 1911 | DRC-2012/3207 | ZSM-PIS-044559 | Lake Tanganyika, Zambia | - | TS | MK144678 |
| Pseudocrenilabrinae | Bathybatini / 'most ancient Tanganyika tribes' | *Hemibates* | *stenosoma* | (Boulenger, 1901) | P-AA-0591 | - | Lake Tanganyika | - | TS | MK144716 |
| Pseudocrenilabrinae | Bathybatini / 'most ancient Tanganyika tribes' | *Bathybates* | *ferox* | Boulenger 1898 | P-AA-0593 | - | Lake Tanganyika | - | TS | MK144677 |
| Pseudocrenilabrinae | Bathybatini / 'most ancient Tanganyika tribes' | *Bathybates* | *fasciatus* | Boulenger 1901 | DRC-2011/948 | ZSM-PIS-041479 | Lake Tanganyika, Zambia | - | TS | MK144676 |
| Pseudocrenilabrinae | Trematocarini / 'most ancient Tanganyika tribes' | *Trematocara* | *unimaculatum* | Boulenger, 1901 | DRC-2012/3190 | ZSM-PIS-044561 | Lake Tanganyika, Zambia | - | TS | MK144779 |
| Pseudocrenilabrinae | Trematocarini / 'most ancient Tanganyika tribes' | *Trematocara* | *macrostoma* | Poll, 1952 | DRC-2012/3140 | ZSM-PIS-044575 | Lake Tanganyika, Zambia | - | TS | MK144778 |
| Pseudocrenilabrinae | Trematocarini / 'most ancient Tanganyika tribes' | *Trematocara* | *cf. variabile* | Poll 1952 | P-AA-0586 | ZSM-PIS-042334 | AS, (Lake Tanganyika) | - | TS | MK144777 |
| Pseudocrenilabrinae | Trematocarini / 'most ancient Tanganyika tribes' | *Trematocara* | *cf. nigrifrons* | Boulenger, 1906 | DRC-2012/3266 | ZSM-PIS-044560 | Lake Tanganyika, Zambia | - | TS | MK144776 |
| Pseudocrenilabrinae | Trematocarini / 'most ancient Tanganyika tribes' | *Trematocara* | *cf. marginatum* | Boulenger 1899 | P-AA-0415 | ZSM-PIS-039570 | AS, Lake Tanganyika | - | TS | MK144775 |
| Pseudocrenilabrinae | Lamprologini | *Variabilichromis* | *moorii* | (Boulenger 1898) | P-AA-0491 | ZSM-PIS-040832 | AS, (Lake Tanganyika) | - | TS | MK144784 & MK144785 |
| Pseudocrenilabrinae | Lamprologini | *Telmatochromis* | *cf. temporalis* | Boulenger 1898 | P-AA-0488 | ZSM-PIS-040829 | AS, (Lake Tanganyika) | - | TS | MK144770 |
| Pseudocrenilabrinae | Lamprologini | *Lamprologus* | *werneri* | Poll 1959 | DRC-2008/401 | ZSM-PIS-037841 | Congo River, DRC | - | TS | MK144726 |
| Pseudocrenilabrinae | Lamprologini | *Lamprologus* | *tigripictilis* | Schelly & Stiassny 2004 | DRC-2013/1912 | ZSM-PIS-042661 | Congo River, DRC | ND1, ND2 & ND4 | TS | MK144725 |
| Pseudocrenilabrinae | Lamprologini | *Lamprologus* | *symoensi* | Poll 1976 | DRC-2012/1680 | - | Lovoi River, DRC | - | TS | MK144724 |
| Pseudocrenilabrinae | Lamprologini | *Telmatochromis* | *sp. "Lufubu"* | - | Uncat | - | AS, Lufubu River, (Zambia) | - | TS | MK144771 |
| Pseudocrenilabrinae | Lamprologini | *Lamprologus* | *sp. Kwango* | - | P-AA-0362 | ZSM-PIS-038830 | Kwango River, DRC | - | TS | MK144723 |
| Pseudocrenilabrinae | Lamprologini | *Lamprologus* | *mocquardi* | Pellegrin 1903 | KIS 2008-002 | ZSM-PIS-037545 | Tshopo River, DRC | - | TS | MK144721 |
| Pseudocrenilabrinae | Lamprologini | *Lamprologus* | *markerti* | Tougas & Stiassny 2014 | DRC-2013/2719 | ZSM-PIS-042658 | Congo River, DRC | - | TS | MK144720 |
| Pseudocrenilabrinae | Lamprologini | *Lamprologus* | *lethops* | Roberts & Stewart 1976 | DRC-2008/311 | ZSM-PIS-038320 | Congo River, DRC | - | TS | MK144719 |
| Pseudocrenilabrinae | Lamprologini | *Lamprologus* | *cf. teugelsi* | Schelly & Stiassny 2004 | DRC-2013/2646 | ZSM-PIS-042643 | Congo River, DRC | - | TS | MK144718 |
| Pseudocrenilabrinae | Lamprologini | *Neolamprologus* | *brichardi* | (Poll 1974) | P-AA-0461 | ZSM-PIS-040802 | AS, (Lake Tanganyika) | - | TS | MK144733 |
| Pseudocrenilabrinae | Lamprologini | *Lepidiolamprologus* | *nkambae* | (Staeck 1978) | P-AA-0454 | ZSM-PIS-040795 | AS, (Lake Tanganyika) | - | TS | MK144727 |
| Pseudocrenilabrinae | Lamprologini | *Lamprologus* | *signatus* | Poll 1952 | P-AA-0462 | ZSM-PIS-040803 | AS, (Lake Tanganyika) | - | TS | MK144722 |
| Pseudocrenilabrinae | Lamprologini | *Chalinochromis* | *sp. "Ndobhoi"* | - | P-AA-0537 | ZSM-PIS-040878 | AS, (Lake Tanganyika) | - | TS | MK144684 |
| Pseudocrenilabrinae | Lamprologini | *Altolamprologus* | *calvus* | (Poll 1978) | P-AA-0536 | ZSM-PIS-040877 | AS, (Lake Tanganyika) | - | TS | MK144669 |
| Pseudocrenilabrinae | Eretmodini | *Spathodus* | *erythrodon* | Boulenger 1900 | P-AA-0503 | ZSM-PIS-040844 | AS, (Lake Tanganyika) | - | TS | MK144765 |
| Pseudocrenilabrinae | Eretmodini | *Eretmodus* | *cyanostictus* | Boulenger 1898 | P-AA-0467 | ZSM-PIS-040808 | AS, (Lake Tanganyika) | - | TS | MK144694 |
| Pseudocrenilabrinae | Cyphotilapiini / 'ancient Tanganyika mouthbrooders' | *Cyphotilapia* | *sp. "Blue Kigoma"* | - | P-AA-0474 | ZSM-PIS-040815 | AS, (Lake Tanganyika) | - | TS | MK144691 |
| Pseudocrenilabrinae | Cyphotilapiini / 'ancient Tanganyika mouthbrooders' | *Trematochromis* | *benthicola* | (Matthes 1962) | P-AA-0587 | ZSM-PIS-043236 | AS, (Lake Tanganyika) | - | TS | MK144780 |
| Pseudocrenilabrinae | Limnochromini / 'ancient Tanganyika mouthbrooders' | *Triglachromis* | *otostigma* | (Regan 1920) | P-AA-0506 | ZSM-PIS-040847 | AS, (Lake Tanganyika) | - | TS | MK144781 |
| Pseudocrenilabrinae | Limnochromini / 'ancient Tanganyika mouthbrooders' | *Greenwoodochromis* | *bellcrossi* | (Poll 1976) | P-AA-0585 | ZSM-PIS-042335 | AS, (Lake Tanganyika) | - | TS | MK144697 |
| Pseudocrenilabrinae | Cyprichromini / 'ancient Tanganyika mouthbrooders' | *Paracyprichromis* | *nigripinnis* | (Boulenger 1901) | P-AA-0452 | ZSM-PIS-040793 | AS, (Lake Tanganyika) | - | TS | MK144748 |
| Pseudocrenilabrinae | Cyprichromini / 'ancient Tanganyika mouthbrooders' | *Cyprichromis* | *leptosoma* | (Boulenger 1898) | P-AA-0476 | ZSM-PIS-040817 | AS, (Lake Tanganyika) | - | TS | MK144692 |
| Pseudocrenilabrinae | Perissodini / 'ancient Tanganyika mouthbrooders' | *Perissodus* | *microlepis* | Boulenger 1898 | P-AA-0582 | ZSM-PIS-042332 | AS, (Lake Tanganyika) | - | TS | MK144751 |
| Pseudocrenilabrinae | Perissodini / 'ancient Tanganyika mouthbrooders' | *Haplotaxodon* | *microlepis* | Boulenger 1906 | P-AA-0499 | ZSM-PIS-040840 | AS, (Lake Tanganyika) | - | TS | MK144715 |
| Pseudocrenilabrinae | Benthochromini / 'ancient Tanganyika mouthbrooders' | *Benthochromis* | *tricoti* | (Poll 1948) | P-AA-0492 | ZSM-PIS-040833 | AS, (Lake Tanganyika) | - | TS | MK144681 |
| Pseudocrenilabrinae | Ectodini / 'ancient Tanganyika mouthbrooders' | *Microdontochromis* | *tenuidentatus* | Poll 1951 | P-AA-0855 | ZSM-PIS-042934 | AS, (Lake Tanganyika) | - | TS | MK144731 |
| Pseudocrenilabrinae | Ectodini / 'ancient Tanganyika mouthbrooders' | *Aulonocranus* | *dewindti* | (Boulenger 1899) | P-AA-0534 | ZSM-PIS-040875 | AS, (Lake Tanganyika) | - | TS | MK144672 |
| Pseudocrenilabrinae | Ectodini / 'ancient Tanganyika mouthbrooders' | *Grammatotria* | *lemairii* | Boulenger 1899 | P-AA-0485 | ZSM-PIS-040826 | AS, (Lake Tanganyika) | - | TS | MK144696 |
| Pseudocrenilabrinae | Ectodini / 'ancient Tanganyika mouthbrooders' | *Cyathopharynx* | *furcifer* | (Boulenger 1898) | P-AA-0470 | ZSM-PIS-040811 | AS, (Lake Tanganyika) | - | TS | MK144690 |
| Pseudocrenilabrinae | Ectodini / 'ancient Tanganyika mouthbrooders' | *Xenotilapia* | *flavipinnis* | Poll 1985 | P-AA-0495 | ZSM-PIS-040836 | AS, (Lake Tanganyika) | - | TS | MK144786 |
| Pseudocrenilabrinae | Ectodini / 'ancient Tanganyika mouthbrooders' | *Callochromis* | *macrops* | (Boulenger 1898) | P-AA-0482 | ZSM-PIS-040823 | AS, (Lake Tanganyika) | - | TS | MK144683 |
| Pseudocrenilabrinae | 'Malagarasi-Orthochromis' | *Orthochromis* | *kasuluensis* | De Vos & Seegers 1998 | DRC-2011/1019 | ZSM-PIS-041455 | Ruchugi River, Tanzania | - | TS | MK144739 |
| Pseudocrenilabrinae | 'Malagarasi-Orthochromis' | *Orthochromis* | *luichensis* | De Vos & Seegers 1998 | DRC-2011/1027 | ZSM-PIS-041445 | Mkuti River, Tanzania | - | TS | MK144741 |
| Pseudocrenilabrinae | 'Malagarasi-Orthochromis' | *Orthochromis* | *malagaraziensis* | (David 1937) | DRC-2011/1029 | ZSM-PIS-041469 | Malagarasi River, Tanzania | - | TS | MK144743 |
| Pseudocrenilabrinae | 'Malagarasi-Orthochromis' | *Orthochromis* | *uvinzae* | De Vos & Seegers 1998 | P-AA-0594 | - | Malagarasi River, Tanzania | ND4 | TS | MK144747 |
| Pseudocrenilabrinae | Haplochromini / Tropheini | *Tropheus* | *polli* | Axelrod 1977 | P-AA-0449 | ZSM-PIS-040790 | AS, (Lake Tanganyika) | - | TS | MK144783 |
| Pseudocrenilabrinae | Haplochromini / Tropheini | *'Haplochromis'* | *horei* | (Günther 1894) | P-AA-0484 | - | AS, (Lake Tanganyika) | - | TS | MK144709 |
| Pseudocrenilabrinae | Haplochromini / Tropheini | *Tropheus* | *moorii "moliro"* |  | P-AA-0487 | ZSM-PIS-040828 | AS, (Lake Tanganyika) | - | TS | MK144782 |
| Pseudocrenilabrinae | Haplochromini / Tropheini | *Petrochromis* | *trewavasae* | Poll 1948 | P-AA-0490 | ZSM-PIS-040831 | AS, (Lake Tanganyika) | - | TS | MK144752 |
| Pseudocrenilabrinae | Haplochromini / Tropheini | *Limnotilapia* | *dardennii* | (Boulenger 1899) | P-AA-0644 | ZSM-PIS-040925 | AS, (Lake Tanganyika) | - | TS | MK144728 |
| Pseudocrenilabrinae | Haplochromini / Tropheini | *Lobochilotes* | *labiatus* | (Boulenger 1898) | P-AA-0641 | ZSM-PIS-040922 | AS, (Lake Tanganyika) | - | TS | MK144729 |
| Pseudocrenilabrinae | Haplochromini / Tropheini | *'Gnathochromis'* | *pfefferi* | Boulenger 1898) | P-AA-0642 | ZSM-PIS-040923 | AS, (Lake Tanganyika) | - | TS | MK144695 |
| Pseudocrenilabrinae | Haplochromini / Tropheini | *Tropheus* | *duboisi* | Marlier 1959 | - | - | - | - | GB |  |
| Pseudocrenilabrinae | Haplochromini / Tropheini | *Simochromis* | *pleurospilus* | Nelissen 1978 | P-AA-0365 | ZSM-PIS-043877 | Lake Tanganyika, Zambia | - | TS | MK144764 |
| Pseudocrenilabrinae | Haplochormini / 'Ctenochromis' | *Ctenochormis* | *pectoralis* | (Pfeffer, 1893) | DRC-2011/1059 | ZSM-PIS-041461 | Chemka springs, Tanzania | - | TS | MK144689 |
| Pseudocrenilabrinae | Haplochromini / 'serranochromines-mt-lineage' | *Serranochromis* | *altus* | Winemiller & Kelso-Winemiller 1991 | P-AA-0666 | - | Namibia | - | TS | MK144762 |
| Pseudocrenilabrinae | Haplochromini / 'serranochromines-mt-lineage' | *Serranochromis* | *robustus* | (Günther 1864) | P-AA-0665 | - | Namibia | - | TS | MK144763 |
| Pseudocrenilabrinae | Haplochromini / 'serranochromines-mt-lineage' | *Chetia welwitschi* | *welwitschi* | (Boulenger 1898) | Z35 | - | Luando River, Angola | ND1 | TS | MK144685 |
| Pseudocrenilabrinae | Haplochromini / 'serranochromines-mt-lineage' | *'Orthochromis'* | *torrenticola* | (Thys van den Audenaerde 1963) | DRC-2012/1723 | 11001 tube-no_AB 42885291 | Lufira River, DRC | - | TS | MK144746 |
| Pseudocrenilabrinae | Haplochromini / 'serranochromines-mt-lineage' | *Pharyngochromis* | *sp. "black bars"* | - | ES-08-B-304 | - | Luando River, Angola | - | TS | MK144753 |
| Pseudocrenilabrinae | Haplochromini / 'serranochromines-mt-lineage' | *Haplochromis* | *polli "Lefini"* | Thys van den Audenaerde 1964 | A8-20-1223 | - | Lefini River, Republic of the Congo | - | TS | MK144711 |
| Pseudocrenilabrinae | Haplochromini / 'serranochromines-mt-lineage' | *Haplochromis* | *sp. Kwango* | - | Kw317 | - | Kwango River, DRC | - | TS | MK144712 |
| Pseudocrenilabrinae | Haplochromini / 'serranochromines-mt-lineage' ('Congo-basin-Haplochromini') | *Haplochromis* | *callichromus* | (Poll, 1948) | P-AA-0677/JM08 190 | tissue only | River Fwa, DRC | - | TS | MK144698 |
| Pseudocrenilabrinae | Haplochromini / 'serranochromines-mt-lineage' ('Congo-basin-Haplochromini') | *Schwetzochromis* | *neodon* | Poll, 1948 | P-AA-0678/JM08 191 | - | River Fwa, DRC | - | TS | MK144761 |
| Pseudocrenilabrinae | Haplochromini / 'serranochromines-mt-lineage' ('Congo-basin-Haplochromini') | *Haplochromis* | *cf. bakongo* | (Thys van den Audenaerde, 1964) | A7-037 | ZSM-PIS-042291 | Kwilu River, DRC | - | TS | MK144700 |
| Pseudocrenilabrinae | Haplochromini / 'serranochromines-mt-lineage' 'LML-Orthochromis' | *'Orthochromis'* | *stormsi* | (Boulenger, 1902) | P-AA-0408 | - | AS, (DRC) | - | TS | MK144745 |
| Pseudocrenilabrinae | Haplochromini / 'Pseudocrenilabrus-group' (Northern-Zambian-Orthochromis) | *'Orthochromis'* | *kalungwishiensis* | (Greenwood & Kullander 1994) | DRC-2011/1001 | ZSM-PIS-041431 | Kalungwishi River, Zambia | - | TS | MK144738 |
| Pseudocrenilabrinae | Haplochromini / 'Pseudocrenilabrus-group' (Northern-Zambian-Orthochromis) | *'Orthochromis'* | *luongoensis* | (Greenwood & Kullander 1994) | DRC-2011/976 | ZSM-PIS-041437 | Luongo River, Zambia | - | TS | MK144742 |
| Pseudocrenilabrinae | Haplochromini / 'Pseudocrenilabrus-group' (Northern-Zambian-Orthochromis) | *'Orthochromis'* | *katumbii* | Schedel, Vreven, Katemo Manda, Abwe, Chocha Manda & Schliewen 2018 | DRC-2011/981 | ZSM-PIS-041450 | Luapula River, Zambia | - | TS | MK144740 |
| Pseudocrenilabrinae | Haplochromini / 'Pseudocrenilabrus-group' (Northern-Zambian-Orthochromis) | *'Orthochromis'* | *mporokoso* | Schedel, Vreven, Katemo Manda, Abwe, Chocha Manda & Schliewen 2018 | DRC-2011/1008 | ZSM-PIS-041429 | Mutoloshi river, Zambia | - | TS | MK144744 |
| Pseudocrenilabrinae | Haplochromini / '*Pseudocrenilabrus*-group' | *New Genus* | New Lufubu Cichlid' | - | DRC-2011/958 | ZSM-PIS-041442 | Muwulwe stream, Zambia | - | TS | MK144735 |
| Pseudocrenilabrinae | Haplochromini / '*Pseudocrenilabrus*-group' | *New Genus* | 'New Kalungwishi cichlid' | - | DRC-2011/1002 | ZSM-PIS-041425 | Kalungwishi River, Zambia | - | TS | MK144734 |
| Pseudocrenilabrinae | Haplochromini / '*Pseudocrenilabrus*-group' | *Pseudocrenilabrus* | *nicholsi* | (Pellegrin 1928) | P-AA-0589 | - | AS, NI | COX3 | TS | MK144756 & MK144757 |
| Pseudocrenilabrinae | Haplochromini / '*Pseudocrenilabrus*-group' | *Pseudocrenilabrus* | *multicolor* | (Schöller 1903) | TI-159 | ZSM-PIS-041574 | AS, (Lake Birkat-Abu-Jumas, Egypt) | - | TS | MK144754 & MK144755 |
| Pseudocrenilabrinae | Haplochromini / '*Pseudocrenilabrus*-group' | *Pseudocrenilabrus* | *philander* | (Weber 1897) | P-AA-0577 | ZSM-PIS-040918 | AS, (Orange River, South Africa) | - | TS | MK144758 |
| Pseudocrenilabrinae | Haplochromini */* 'ocellated eggspot Haplochromini' *(Astatoreochromis)* | *Astatoreochromis* | *straeleni* | (Poll 1944) | P-AA-0580 | tissue only | AS, NI | - | TS | MK144671 |
| Pseudocrenilabrinae | Haplochromini / 'ocellated eggspot Haplochromini' (riverine & modern Haplochromini) | *Haplochromis* | *nyererei* | (Witte-Maas & Witte 1985) | - | - | - | - | GB | NC_028011 |
| Pseudocrenilabrinae | Haplochromini / 'ocellated eggspot Haplochromini' (riverine & modern Haplochromini) | *Haplochromis* | *flaviijosephi* | (Lortet, 1883) | P-AA-0412 | - | AS, (Israel) | - | TS | MK144708 |
| Pseudocrenilabrinae | Haplochromini / 'ocellated eggspot Haplochromini' (riverine & modern Haplochromini) | *Haplochromis* | *obliquidens* | (Hilgendrof, 1888) | P-AA-0513 | ZSM-PIS-040854 | AS, (Lake Victoria) | - | TS | MK144710 |
| Pseudocrenilabrinae | Haplochromini / 'ocellated eggspot Haplochromini' (riverine & modern Haplochromini) | *'Haplochromis'* | *fasciatus* | (Perugia, 1892) | BC08-26 | ZSM-PIS-038604 | Lower Congo, DRC | - | TS | MK144707 |
| Pseudocrenilabrinae | Haplochromini / 'ocellated eggspot Haplochromini' (riverine & modern Haplochromini) | *Haplochromis* | *stappersii* | Poll, 1943 | P-AA-0369 | ZSM-PIS-038832 | AS, (DRC) | - | TS | MK144713 |
| Pseudocrenilabrinae | Haplochromini / 'ocellated eggspot Haplochromini' (riverine & modern Haplochromini) | *Haplochromis* | *cf. kilossana "Sonjo"* |  | DRC-2011/1036 | ZSM-PIS-041441 | Sonjo stream, Tanzania | - | TS | MK144704 |
| Pseudocrenilabrinae | Haplochromini / 'ocellated eggspot Haplochromini' (riverine & modern Haplochromini) | *Haplochromis* | *cf. kilossana "Ruaha"* | - | DRC-2011/1039 | voucher only | Little Ruaha, Tanzania | - | TS | MK144703 |
| Pseudocrenilabrinae | Haplochromini / 'ocellated eggspot Haplochromini' (riverine & modern Haplochromini) | *'Haplochromis'* | *demeusii* | (Boulenger 1899) | DRC-2008/531 | ZSM-PIS-038376 | Congo River, DRC | ND1 & ND4 | TS | MK144705 |
| Pseudocrenilabrinae | Haplochromini / 'ocellated eggspot Haplochromini' (riverine & modern Haplochromini) | *Haplochromis* | *cf. brutoni yellow* |  | P-AA-0368 | ZSM-PIS-038838 | AS, (Burundi) | - | TS | MK144701 |
| Pseudocrenilabrinae | Haplochromini / 'ocellated eggspot Haplochromini' (riverine & modern Haplochromini) | *Haplochromis* | *desfontainii* | (Lacepède 1802) | P-AA-0407 | - | AS, (Tunisia) | - | TS | MK144706 |
| Pseudocrenilabrinae | Haplochormini / 'ocellated eggspot Haplochromini' (Lake Malawi species flock) | *Nimbochromis* | *linni* | (Burgess & Axelrod 1975) | - | - | - | - | GB | NC_018558.1 |
| Pseudocrenilabrinae | Haplochormini / 'ocellated eggspot Haplochromini' (Lake Malawi species flock) | *Cheilochromis* | *euchilus* | (Trewavas 1935) | - | - | - | - | GB | NC_020672.1 |
| Pseudocrenilabrinae | Haplochormini / 'ocellated eggspot Haplochromini' (Lake Malawi species flock) | *Cynotilapia* | *afra* | (Günther 1894) | - | - | - | - | GB | NC_018564.1 |
| Pseudocrenilabrinae | Haplochormini / 'ocellated eggspot Haplochromini' (Lake Malawi species flock) | *Rhamphochromis* | *esox* | (Boulenger 1908) | - | - | - | - | GB | NC_018563.1 |
| Pseudocrenilabrinae | Haplochormini / 'ocellated eggspot Haplochromini' (Lake Malawi species flock) | *Genyochromis* | *mento* | Trewavas 1935 | - | - | - | - | GB | NC_018562.1 |
| Pseudocrenilabrinae | Haplochormini / 'ocellated eggspot Haplochromini' (Lake Malawi species flock) | *Dimidiochromis* | *compressiceps* | (Boulenger 1908) | - | - | - | - | GB | NC_018561.1 |
| Pseudocrenilabrinae | Haplochormini / 'ocellated eggspot Haplochromini' (Lake Malawi species flock) | *Pseudotropheus* | *crabro* | (Ribbink & Lewis 1982) | - | - | - | - | GB | NC_018559.1 |
| Pseudocrenilabrinae | Haplochormini / 'ocellated eggspot Haplochromini' (Lake Malawi species flock) | *Petrotilapia* | *nigra* | Marsh 1983 | - | - | - | - | GB | NC_018557.1 |
| Pseudocrenilabrinae | Haplochormini / 'ocellated eggspot Haplochromini' (Lake Malawi species flock) | *Diplotaxodon* | *limnothrissa* | Turner 1994 | - | - | - | - | GB | NC_018556.1 |
| Pseudocrenilabrinae | Haplochormini / 'ocellated eggspot Haplochromini' (Lake Malawi species flock) | *Trematocranus* | *placodon* | (Regan 1922) | - | - | - | - | GB | NC_018555.1 |
| Pseudocrenilabrinae | Haplochormini / 'ocellated eggspot Haplochromini' (Lake Malawi species flock) | *Maylandia* | *zebra* | (Boulenger 1899) | - | - | - | - | GB | KT221043 |
| Pseudocrenilabrinae | Haplochormini / 'ocellated eggspot Haplochromini' (Lake Malawi species flock) | *Fossorochromis* | *rostratus* | (Boulenger 1899) | - | - | - | - | GB | KT715809 |
| Pseudocrenilabrinae | Haplochormini / 'ocellated eggspot Haplochromini' (Lake Malawi species flock) | *Protomelas* | *annectens* | (Regan 1922) | - | - | - | - | GB | NC_027953 |
| Pseudocrenilabrinae | Haplochormini / 'ocellated eggspot Haplochromini' (Lake Malawi species flock) | *Placidochromis* | *longimanus* | (Trewavas 1935) | - | - | - | - | GB | NC_028156 |
| Pseudocrenilabrinae | Haplochormini / 'ocellated eggspot Haplochromini' (Lake Malawi species flock) | *Alticorpus* | *geoffreyi* | Snoeks & Walapa 2004 | - | - | - | - | GB | NC_028033 |
| Pseudocrenilabrinae | Haplochormini / 'ocellated eggspot Haplochromini' (Lake Malawi species flock) | *Copadichromis* | *virginalis* | (Iles 1960) | - | - | - | - | GB | KU144677 |
| Pseudocrenilabrinae | Haplochormini / 'ocellated eggspot Haplochromini' (Lake Malawi species flock) | *Haplochromis* | *callipterus* | (Günther, 1894) | P-AA-0559 | ZSM-PIS-040900 | AS, (Lake Malawi) | - | TS | MK144699 |
| Pseudocrenilabrinae | Haplochormini / 'ocellated eggspot Haplochromini' (Lake Malawi species flock) | *Haplochromis* | *cf. callipterus* | (Günther, 1894) | DRC-2011/945 |  | Kiwira River, Tanzania | - | TS | MK144702 |
| Pseudocrenilabrinae | Haplochormini / 'ocellated eggspot Haplochromini' (Lake Malawi species flock) | *Melanochromis* | *auratus* | Boulenger, 1897 | P-AA-0520 | ZSM-PIS-040861 | AS, (Lake Malawi) | - | TS | MK144730 |
| Pseudocrenilabrinae | Haplochormini / 'ocellated eggspot Haplochromini' (Lake Malawi species flock) | *Alticorpus* | *mentale* | Stauffer & McKaye 1988 | P-AA-0686 |  | AS (Senga Bay, Lake Malawi) | - | TS | MK144668 |
| Pseudocrenilabrinae | Haplochormini / 'ocellated eggspot Haplochromini' (Lake Malawi species flock) | *Aulonocara* | *baenschi* | Meyer & Riehl 1985 | P-AA-0542 | ZSM-PIS-040883 | AS, (Lake Malawi) | - | TS | MK144673 |
| Pseudocrenilabrinae | Haplochormini / 'ocellated eggspot Haplochromini' (Lake Malawi species flock) | *Aulonocara* | *stuartgranti* | Meyer & Riehl 1985 | P-AA-0569 | ZSM-PIS-040910 | AS, (Lake Malawi) | - | TS | MK144674 |
| Pseudocrenilabrinae | Haplochromini / '*Haplochromis*' *vanheusdeni* | *Haplochromis* | *vanheusdeni* | Schedel, Friel & Schliewen 2014 | DRC-2011/1032 | ZSM-PIS-041440 | Sonjo stream, Tanzania | - | TS | MK144714 |
| Pseudocrenilabrinae | Haplochromini /'*Orthochromis*' sp. 'Lufubu ' | *'Orthochromis'* | indermauri | Schedel, Vreven, Katemo Manda, Abwe, Chocha Manda & Schliewen 2018 | 482 | - | Lufubu River, Zambia | - | TS | MK144737 |
