# Supplementary material for: East African cichlid lineages (Teleostei: Cichlidae) might be older than their ancient host lakes: new divergence estimates for the east African cichlid radiation
Source: BMC Evol Biol. 2019 Apr 25;19:94. doi: 10.1186/s12862-019-1417-0 (PMC6482553; doi:10.1186/s12862-019-1417-0)
Supplement: Supplementary file 2 — Table S3. Overview of the taxon sampling for the nuclear markers (RAG1, ENC1, Rh1 and ttna TMO) with corresponding Genbank accession numbers. (DOCX 75 kb) [file 12862_2019_1417_MOESM2_ESM.docx]

**SchedelEtal_Table_A2**

Table A.2

Comprehensive list of mean divergence ages and their corresponding 95% HPD age ranges of selected nodes. (Node numbers 1 to 65 correspond to numbers depicted in Fig. 2)

Table A.2: Comprehensive list of mean divergence ages and their corresponding 95 % HPD age ranges of selected nodes

| **Node corresponding numbers in Fig 2.:** | **Comment to clade (MRCA):** | **Set 1** | **95 % HPD:** |  | **Set 2** | **95 % HPD:** |  | **Set 3** | **95 % HPD:** |  | **Set 4** | **95 % HPD:** |  |
| --- | --- | --- | --- | --- | --- | --- | --- | --- | --- | --- | --- | --- | --- |
|  |  | **Mean age** | **Min** | **Max** | **Mean age** | **Min** | **Max** | **Mean age** | **Min** | **Max** | **Mean age** | **Min** | **Max** |
| **1** | Cichlidae | 103,0314 | 85,925 | 123,253 | 102,8129 | 84,3143 | 122,664 | 92,909 | 85,4575 | 101,9417 | 92,862 | 85,4302 | 101,9017 |
| **2** | (Cichlinae, Pseudocrenilabrinae) | 91,0594 | 77,68 | 105,7391 | 90,7845 | 77,2149 | 105,7982 | 84,3824 | 75,6065 | 93,1207 | 84,3747 | 75,7104 | 93,2522 |
| **3** | Cichlinae | 78,3504 | 67,8887 | 90,1163 | 78,2496 | 67,6889 | 89,8805 | 73,9711 | 66,2894 | 82,457 | 73,9293 | 66,267 | 82,325 |
| **4** | Cichlasomatini 'Andinoacarines' | 18,3675 | 11,111 | 26,2418 | 18,4477 | 11,42 | 26,4938 | 17,9599 | 11,1928 | 25,399 | 17,6049 | 10,6744 | 24,7338 |
| **5** | 'mesoamerican' Heroini | 34,749 | 28,2299 | 40,6297 | 34,9755 | 28,774 | 40,6229 | 34,1784 | 27,9636 | 39,9216 | 34,0646 | 27,5999 | 39,7643 |
| **6** | Cichliasomatini | 51,1962 | 41,9035 | 60,577 | 51,5542 | 41,832 | 61,118 | 49,3258 | 41,595 | 57,7713 | 49,3688 | 41,2132 | 57,48 |
| **7** | Heroini | 54,1364 | 47,9184 | 61,364 | 54,234 | 48,1494 | 61,951 | 52,5851 | 47,5886 | 57,9397 | 52,6589 | 47,4528 | 58,379 |
| **8** | (Heroini, Cichlasomatini) | 62,6612 | 54,6249 | 71,1002 | 62,8211 | 54,8431 | 71,1648 | 60,1759 | 54,2129 | 66,675 | 60,1892 | 53,7458 | 66,6049 |
| **9** | Pseudocrenilabrinae | 65,1957 | 53,487 | 78,4808 | 65,5267 | 53,1246 | 79,3572 | 61,2964 | 51,3702 | 72,1322 | 60,7893 | 50,8741 | 71,1 |
| **10** | Haplotilapiines (sensu Schliewen & Stiassny, 2003) | 49,4034 | 39,6412 | 60,1658 | 49,1858 | 38,9119 | 59,7813 | 46,6448 | 38,4791 | 55,8189 | 45,9644 | 37,9837 | 54,4918 |
| **11** | (Coptodonini, (Pelmatolapiini, (Oreochromini, Austrotilapiines))) | 38,129 | 31,537 | 45,2448 | 38,0037 | 30,8696 | 45,1842 | 36,295 | 30,4736 | 42,3033 | 35,8315 | 30,4065 | 41,5984 |
| **12** | Oreochromini | 24,263 | 17,9067 | 30,9785 | 24,2157 | 17,7417 | 31,0654 | 23,1034 | 16,9407 | 29,3369 | 22,9458 | 17,2702 | 29,1104 |
| **13** | Oreochromini (excluding *Danakilia*) | 21,0608 | 15,2969 | 26,8542 | 21,0392 | 15,3779 | 27,2053 | 20,0534 | 14,5942 | 25,758 | 19,9561 | 14,7906 | 25,533 |
| **14** | Australotilapiines (sensu Schwarzer et al., 2009) | 33,9358 | 28,2124 | 39,9501 | 33,8822 | 27,9198 | 40,3155 | 32,3748 | 27,535 | 37,6323 | 31,9814 | 27,1654 | 36,9166 |
| **15** | Steatocranini | 12,8987 | 8,3909 | 18,2 | 12,8818 | 8,764 | 17,847 | 12,2352 | 8,5 | 16,6371 | 12,1582 | 7,9201 | 16,6782 |
| **16** | (Steatocranini, East African Radiation) | 32,544 | 27,317 | 38,4861 | 32,4349 | 26,5944 | 38,5929 | 30,9955 | 26,2513 | 35,9657 | 30,6184 | 26,588 | 35,4011 |
| **17** | East African Radiation (EAR) | 30,4951 | 25,3205 | 36,301 | 30,3915 | 25,1794 | 36,4966 | 29,0431 | 24,5178 | 33,5194 | 28,7062 | 24,4255 | 33,1473 |
| **18** | Trematocarini | 17,0964 | 12,5055 | 22,1645 | 17,0424 | 12,2655 | 22,707 | 16,2143 | 12,411 | 20,5476 | 16,1268 | 11,8884 | 20,4611 |
| **19** | (Bathybatini, Trematocarini) | 25,7518 | 20,7101 | 30,8822 | 25,6662 | 20,4901 | 31,681 | 24,5671 | 20,358 | 29,1061 | 24,2698 | 20,1653 | 28,8611 |
| **20** | Bathybatini (including *Hemibates*) | 21,8406 | 16,5847 | 27,1086 | 21,7805 | 16,5298 | 27,807 | 20,8004 | 16,1823 | 25,5116 | 20,6204 | 16,884 | 25,3372 |
| **21** | *Bathybates* | 13,9889 | 9,8023 | 18,6524 | 13,9755 | 9,7151 | 18,3333 | 13,29 | 9,3283 | 17,4526 | 13,2418 | 9,5317 | 17,3035 |
| **22** | *Bathybates* (excluding *Bathybates minor*) | 8,6338 | 5,8734 | 11,6455 | 8,6251 | 5,8639 | 11,5727 | 8,2221 | 5,7157 | 11,226 | 8,2037 | 5,7664 | 10,6679 |
| **23** | East African Radiation (EAR) excluding Boulengerochromini | 29,4319 | 24,3716 | 34,6971 | 29,3305 | 24,398 | 34,9172 | 28,0253 | 23,7922 | 32,5131 | 27,7076 | 23,7215 | 32,1685 |
| **24** | MVhL-clade (sensu Takahashi et al., 2001) | 25,0685 | 20,828 | 29,5935 | 24,965 | 20,7508 | 29,7204 | 23,837 | 20,1617 | 27,6863 | 23,5998 | 20,1767 | 27,3334 |
| **25** | Lamprologini | 16,2073 | 12,6477 | 20,1361 | 15,9178 | 12,1573 | 19,8331 | 15,4217 | 12,491 | 18,9624 | 15,2719 | 12,2265 | 18,4911 |
| **26** | 'Lacustrine lamprologines' | 15,1388 | 11,702 | 18,8035 | 14,9001 | 11,2828 | 18,5234 | 14,3785 | 11,2267 | 17,8476 | 14,2833 | 11,2605 | 17,3269 |
| **27** | 'Non-ossfied lacustrine Lamprologines' | 13,2597 | 10,78 | 16,7905 | 12,989 | 9,7272 | 16,5448 | 12,6094 | 9,51 | 15,8583 | 12,5084 | 9,7489 | 15,512 |
| **28** | 'Ossified lamprologines' | 11,2456 | 7,693 | 14,9579 | 11,0441 | 7,3831 | 14,7351 | 10,6442 | 7,2621 | 14,1902 | 10,6591 | 7,3866 | 13,973 |
| **29** | 'Lower Congo *Lamprologus* clade' | 6,9406 | 4,5489 | 9,7441 | 6,9117 | 4,4129 | 9,592 | 6,6791 | 4,4964 | 9,624 | 6,6156 | 4,3062 | 9,485 |
| **30** | (*Telmatochromis* sp. 'Lufubu', *Neolamprologus brichardi*) | 4,5901 | 2,4431 | 6,7965 | 4,5464 | 2,4972 | 6,7496 | 4,3704 | 2,3328 | 6,5517 | 4,3182 | 2,3457 | 6,352 |
| **31** | (*Lamprologus werneri*, (*Telmatochromis* cf. *temporalis*, *Lamprologus symoensi*)) | 5,4159 | 3,4452 | 7,5176 | 5,3018 | 3,4156 | 7,2329 | 5,1464 | 3,3493 | 7,881 | 5,0549 | 3,3551 | 6,877 |
| **32** | (*Telmatochromis* cf. *temporalis*, *Lamprologus symoensi*) | 2,7234 | 1,3893 | 4,1686 | 2,6684 | 1,3284 | 4,548 | 2,593 | 1,3717 | 3,9915 | 2,5472 | 1,343 | 3,8675 |
| **33** | Eretmodini | 8,0802 | 4,2006 | 12,4994 | 8,0349 | 4,172 | 12,3852 | 7,7239 | 4,174 | 11,9787 | 7,5983 | 3,9246 | 11,9937 |
| **34** | C-lineage (sensu Clabaut et al., 2005), Eretmodini | 24,1548 | 20,28 | 28,5347 | 24,115 | 19,7751 | 28,4844 | 22,9658 | 19,3443 | 26,674 | 22,8029 | 19,4036 | 26,3169 |
| **35** | Cyphotilapini | 15,2585 | 8,9481 | 20,8567 | 15,2713 | 9,561 | 21,1935 | 14,076 | 8,3053 | 19,5293 | 14,1571 | 8,7094 | 19,2454 |
| **36** | Limnochromini | 11,3639 | 5,6366 | 17,6506 | 11,4877 | 5,7576 | 17,2823 | 10,7198 | 5,5278 | 16,2953 | 10,7087 | 5,2009 | 16,303 |
| **37** | Perisodini | 6,5862 | 3,2939 | 10,3296 | 6,4384 | 3,665 | 10,426 | 6,2373 | 3,1438 | 9,903 | 6,182 | 3,1552 | 9,5889 |
| **38** | Cyprichromini | 11,0176 | 6,4104 | 15,571 | 11,1376 | 6,4607 | 15,6473 | 10,5417 | 6,1837 | 15,301 | 10,3791 | 6,1993 | 14,5342 |
| **39** | 'benthopelagic LT clade' | 17,159 | 13,956 | 21,3044 | 17,1382 | 13,1076 | 21,526 | 16,2878 | 12,474 | 19,8685 | 16,1399 | 12,8333 | 19,5432 |
| **40** | Ectodini | 14,9233 | 11,3377 | 18,5871 | 14,7893 | 11,3431 | 18,382 | 14,1907 | 10,9164 | 17,3813 | 14,055 | 11,1834 | 17,695 |
| **41** | 'Malagarasi-*Orthochromis*' | 12,1714 | 8,5121 | 16,34 | 12,1478 | 8,5526 | 16,2083 | 11,6559 | 8,1208 | 15,1794 | 11,4479 | 8,629 | 14,9036 |
| **42** | ('Malagarasi-*Orthochromis*', Haplochromini) | 19,6492 | 16,3851 | 23,2901 | 19,57 | 16,2256 | 23,1 | 18,733 | 15,8818 | 21,692 | 18,467 | 15,8856 | 21,2612 |
| **43** | Haplochromini | 17,6971 | 14,6583 | 20,9198 | 17,6208 | 14,6593 | 20,8665 | 16,8943 | 14,2194 | 19,5351 | 16,6421 | 14,2542 | 19,155 |
| **44** | Haplochromini (excluding *Ctenochromis pectoralis*) | 16,454 | 13,6574 | 19,3919 | 16,3975 | 13,594 | 19,3555 | 15,7069 | 13,2759 | 18,1736 | 15,4883 | 13,1513 | 17,7521 |
| **45** | ('Congo-basin-Haplochromini', ('serranochromines-mt-lineage', 'LML-*Orthochromis*')) | 14,2469 | 11,4514 | 17,1186 | 14,2162 | 11,3478 | 17,105 | 13,5814 | 11,1478 | 16,622 | 13,4147 | 11,1822 | 15,854 |
| **46** | ('serranochromines-mt-lineage sensu stricto', *Chetia welwitschi)* | 9,8572 | 7,3944 | 12,3945 | 9,831 | 7,4351 | 12,3651 | 9,292 | 7,87 | 11,4924 | 9,2535 | 7,1194 | 11,412 |
| **47** | 'serrranochromines-mt-lineage sensu stricto' | 5,8737 | 4,0244 | 7,8182 | 5,8097 | 3,9564 | 7,7449 | 5,4292 | 3,7413 | 7,1305 | 5,4628 | 3,7896 | 7,2423 |
| **48** | (*Serranochromis altus*, *Pharyngochromis* sp. "black bars") | 4,7099 | 2,8688 | 6,7622 | 4,639 | 2,8198 | 6,6586 | 4,3461 | 2,6451 | 6,1296 | 4,3927 | 2,7571 | 6,2778 |
| **49** | ('*Orthochromis*' sp. 'Lufubu ', ('*Pseudocrenilabrus*-group', 'ocellated eggspot Haplochromini') | 16,0932 | 13,3085 | 18,9535 | 16,0463 | 13,3045 | 18,9507 | 14,9137 | 13,556 | 17,866 | 15,1486 | 12,9122 | 17,4564 |
| **50** | *Pseudocrenilabrus*-group' | 12,5659 | 9,909 | 15,2359 | 12,5606 | 9,932 | 15,4046 | 12,0204 | 9,7076 | 14,4622 | 11,8174 | 9,6311 | 14,1797 |
| **51** | (*Pseudocrenilabrus multicolor*, *Pseudocrenilabrus nicholsi*) | 2,3689 | 1,2459 | 3,6697 | 2,3382 | 1,2272 | 3,5984 | 2,2422 | 1,1783 | 3,4217 | 2,2022 | 1,1975 | 3,3443 |
| **52** | 'ocellated eggspot Haplochromini' | 15,0248 | 12,4072 | 17,8169 | 14,9847 | 12,318 | 17,733 | 14,326 | 12,465 | 16,7076 | 14,1369 | 12,168 | 16,4227 |
| **53** | 'ocellated eggspot Haplochromini' excluding *Astatoreochromis* | 12,5302 | 10,3124 | 15,683 | 12,5318 | 10,2071 | 15,121 | 11,8974 | 9,7621 | 13,9741 | 11,7761 | 9,7748 | 13,6684 |
| **54** | (Tropheini, '*Haplochromis*' *vanheusdeni*) | 11,1926 | 8,8344 | 13,5885 | 11,1863 | 8,7906 | 13,6773 | 10,6342 | 8,4185 | 12,7737 | 10,5133 | 8,4675 | 12,6104 |
| **55** | (*Tropheus moorii*, *Tropheus polli*) | 3,7483 | 1,7315 | 6,639 | 3,6915 | 1,6931 | 6,856 | 3,5393 | 1,5761 | 5,7291 | 3,4942 | 1,6332 | 5,6435 |
| **56** | Tropheini (excluding Tropheus moorii, Tropheus polli) | 7,665 | 5,733 | 9,735 | 7,6713 | 5,7379 | 9,7632 | 7,2867 | 5,4685 | 9,2169 | 7,2172 | 5,4373 | 8,9939 |
| **57** | Tropheini | 9,2372 | 7,841 | 11,5957 | 9,2361 | 7,1237 | 11,5901 | 8,7769 | 6,812 | 10,8895 | 8,6892 | 6,7747 | 10,6991 |
| **58** | (*Haplochromis demeusii*, *Haplochromis fasciatus*) | 5,6672 | 3,5472 | 7,9615 | 5,6838 | 3,4159 | 7,9225 | 5,3349 | 3,2305 | 7,4917 | 5,3526 | 3,354 | 7,404 |
| **59** | 'riverine & modern Haplochromini' | 10,035 | 8,644 | 12,2799 | 10,0896 | 7,9089 | 12,1005 | 9,5252 | 7,6775 | 11,4845 | 9,4235 | 7,7173 | 11,2312 |
| **60** | (LVRS, *Haplochromis stappersii*) | 1,0395 | 0,5321 | 1,6302 | 1,0508 | 0,5433 | 1,6442 | 0,9967 | 0,5183 | 1,545 | 0,9859 | 0,509 | 1,5256 |
| **61** | Lake Victoria Region Superflock (LVRS) | 0,3266 | 0,1246 | 0,5667 | 0,3276 | 0,1218 | 0,5713 | 0,3113 | 0,1206 | 0,5417 | 0,3077 | 0,1191 | 0,5306 |
| **62** | ('riverine & modern Haplochromini', Lake Malawi species flock) | 10,621 | 8,5545 | 12,89 | 10,6732 | 8,5395 | 12,8865 | 10,0865 | 8,1462 | 12,614 | 9,9765 | 8,1991 | 11,7758 |
| **63** | Lake Malawi species flock | 4,264 | 3,607 | 5,5929 | 4,287 | 3,403 | 5,6091 | 4,0573 | 2,9278 | 5,301 | 4,07 | 2,9339 | 5,2628 |
| **64** | 'primarily Mbuna taxa' | 0,6322 | 0,4145 | 0,8758 | 0,6327 | 0,413 | 0,8617 | 0,6032 | 0,4089 | 0,8267 | 0,5993 | 0,3949 | 0,8147 |
| **65** | 'primarily LM Sanddweller' | 0,7338 | 0,473 | 1,33 | 0,731 | 0,4656 | 1,233 | 0,6962 | 0,451 | 0,9659 | 0,6903 | 0,4488 | 0,9572 |
|  |  |  |  |  |  |  |  |  |  |  |  |  |  |

| **Node corresponding numbers in Fig 2.:** | **Comment to clade (MRCA):** | **Set 5** | **95 % HPD:** |  | **Set 6** | **95 % HPD:** |  | **Set 7** | **95 % HPD:** |  | **Set 8** | **95 % HPD:** |  |
| --- | --- | --- | --- | --- | --- | --- | --- | --- | --- | --- | --- | --- | --- |
|  |  | **Mean age** | **Min** | **Max** | **Mean age** | **Min** | **Max** | **Mean age** | **Min** | **Max** | **Mean age** | **Min** | **Max** |
| **1** | Cichlidae | 103,6848 | 85,6301 | 122,9166 | 93,274 | 85,4002 | 102,4513 | 104,1082 | 85,6632 | 123,9736 | 93,2104 | 85,4449 | 102,4271 |
| **2** | (Cichlinae, Pseudocrenilabrinae) | 91,7446 | 77,9443 | 106,6794 | 84,8826 | 76,5248 | 94,2266 | 91,9409 | 77,7873 | 106,9621 | 84,8171 | 76,347 | 93,8839 |
| **3** | Cichlinae | 79,063 | 67,8009 | 90,5431 | 74,4524 | 66,6786 | 82,7105 | 79,2137 | 67,654 | 90,9685 | 74,3603 | 66,4562 | 82,5124 |
| **4** | Cichlasomatini 'Andinoacarines' | 18,5274 | 11,104 | 26,4913 | 18,0824 | 10,8372 | 26,483 | 18,9232 | 11,7219 | 26,5407 | 17,7725 | 10,6339 | 25,446 |
| **5** | 'mesoamerican' Heroini | 35,0694 | 29,103 | 41,1643 | 34,1895 | 27,9559 | 39,8818 | 34,9988 | 28,8924 | 40,9946 | 34,2787 | 28,335 | 39,886 |
| **6** | Cichliasomatini | 52,1127 | 42,406 | 61,6908 | 49,7885 | 41,519 | 57,7185 | 52,0745 | 42,5108 | 61,5742 | 49,5828 | 41,3605 | 57,8812 |
| **7** | Heroini | 54,6295 | 47,9578 | 61,5361 | 52,8767 | 47,6946 | 58,341 | 54,5133 | 48,1306 | 61,5916 | 52,8357 | 47,4432 | 58,1884 |
| **8** | (Heroini, Cichlasomatini) | 63,3726 | 55,1311 | 72,3032 | 60,5364 | 54,3839 | 67,3209 | 63,3877 | 54,9757 | 72,821 | 60,4394 | 54,2358 | 67,361 |
| **9** | Pseudocrenilabrinae | 65,2345 | 52,8642 | 78,8787 | 62,1443 | 51,4572 | 72,7788 | 65,3317 | 53,2266 | 78,728 | 61,3039 | 51,2464 | 71,9941 |
| **10** | Haplotilapiines (sensu Schliewen & Stiassny, 2003) | 49,8615 | 39,5815 | 60,4877 | 47,1746 | 38,4535 | 56,137 | 50,2465 | 40,768 | 60,7638 | 46,927 | 38,7406 | 56,1012 |
| **11** | (Coptodonini, (Pelmatolapiini, (Oreochromini, Austrotilapiines))) | 38,8283 | 32,179 | 46,1837 | 36,7293 | 31,801 | 42,5118 | 38,9062 | 32,0043 | 46,0303 | 36,5761 | 30,5762 | 42,6148 |
| **12** | Oreochromini | 24,9202 | 18,1795 | 31,9354 | 23,4666 | 17,519 | 29,9749 | 25,7485 | 18,7495 | 33,0478 | 24,2218 | 17,9327 | 30,7696 |
| **13** | Oreochromini (excluding *Danakilia*) | 21,6379 | 15,7189 | 27,7343 | 20,2795 | 14,8553 | 26,314 | 22,2055 | 15,9775 | 28,5516 | 20,856 | 15,2954 | 27,1575 |
| **14** | Australotilapiines (sensu Schwarzer et al., 2009) | 34,6769 | 28,6847 | 40,8388 | 32,7992 | 27,9637 | 37,7047 | 34,6915 | 28,7716 | 40,9993 | 32,6515 | 27,5093 | 37,9188 |
| **15** | Steatocranini | 13,2715 | 8,5265 | 18,6518 | 12,6029 | 8,1365 | 17,5001 | 13,1277 | 8,4308 | 18,2823 | 12,6186 | 8,1175 | 17,4297 |
| **16** | (Steatocranini, East African Radiation) | 33,2426 | 27,4475 | 39,3033 | 31,3825 | 26,8836 | 36,1939 | 33,1739 | 27,2476 | 39,22 | 31,2257 | 26,3333 | 36,3282 |
| **17** | East African Radiation (EAR) | 31,1712 | 25,7794 | 36,9931 | 29,41 | 25,689 | 33,7635 | 29,9798 | 25,5157 | 36,6569 | 29,2595 | 24,7728 | 34,678 |
| **18** | Trematocarini | 17,4141 | 12,3523 | 22,702 | 16,4894 | 12,3434 | 21,27 | 17,4298 | 12,5512 | 22,4533 | 16,2829 | 11,8838 | 20,8522 |
| **19** | (Bathybatini, Trematocarini) | 26,3555 | 21,2005 | 31,6138 | 24,8673 | 20,8733 | 29,309 | 26,2226 | 21,2867 | 31,6968 | 24,7154 | 20,2772 | 29,2924 |
| **20** | Bathybatini (including *Hemibates*) | 22,3604 | 17,812 | 27,8607 | 21,1206 | 16,7391 | 25,6946 | 22,2677 | 17,783 | 27,8998 | 21,0021 | 16,4469 | 25,8622 |
| **21** | *Bathybates* | 14,2347 | 9,6574 | 18,8185 | 13,6781 | 9,6863 | 17,8524 | 14,2342 | 10,35 | 18,947 | 13,4139 | 9,5357 | 17,437 |
| **22** | *Bathybates* (excluding *Bathybates minor*) | 8,7971 | 6,858 | 11,877 | 8,414 | 5,7992 | 11,3668 | 8,7768 | 5,9603 | 11,7554 | 8,331 | 5,8185 | 11,161 |
| **23** | East African Radiation (EAR) excluding Boulengerochromini | 30,0919 | 24,8065 | 35,6417 | 28,3877 | 24,1974 | 32,6063 | 29,9798 | 24,5474 | 35,3186 | 28,2291 | 23,8045 | 32,78 |
| **24** | MVhL-clade (sensu Takahashi et al., 2001) | 25,6707 | 21,2301 | 30,3266 | 24,1944 | 20,6427 | 27,9891 | 25,4912 | 21,1808 | 30,3063 | 24,0392 | 20,1106 | 27,8402 |
| **25** | Lamprologini | 16,4365 | 12,7571 | 20,3599 | 15,6463 | 12,3935 | 19,392 | 16,3908 | 12,7246 | 20,2481 | 15,5513 | 12,4466 | 19,66 |
| **26** | 'Lacustrine lamprologines' | 15,3761 | 11,8225 | 19,695 | 14,6354 | 11,5543 | 17,9383 | 15,3112 | 11,8093 | 19,244 | 14,516 | 11,5511 | 17,833 |
| **27** | 'Non-ossfied lacustrine Lamprologines' | 13,473 | 10,1252 | 16,899 | 12,8143 | 9,7299 | 15,8976 | 13,4291 | 10,492 | 16,8094 | 12,7164 | 9,8032 | 15,798 |
| **28** | 'Ossified lamprologines' | 11,4288 | 7,6798 | 15,2494 | 10,8778 | 7,3768 | 14,4036 | 11,3764 | 7,6954 | 15,2772 | 10,8213 | 7,2953 | 14,2435 |
| **29** | 'Lower Congo *Lamprologus* clade' | 7,1051 | 4,6742 | 9,7914 | 4,4662 | 4,4442 | 9,4483 | 7,0126 | 4,6667 | 9,5682 | 6,707 | 4,5254 | 9,1703 |
| **30** | (*Telmatochromis* sp. 'Lufubu', *Neolamprologus brichardi*) | 4,708 | 2,5813 | 6,9381 | 4,4662 | 2,4656 | 6,6327 | 4,6197 | 2,5091 | 6,9 | 4,4404 | 2,4181 | 6,4455 |
| **31** | (*Lamprologus werneri*, (*Telmatochromis* cf. *temporalis*, *Lamprologus symoensi*)) | 5,4768 | 3,5947 | 7,5519 | 5,2 | 3,432 | 7,103 | 5,4916 | 3,5955 | 7,6156 | 5,1531 | 3,395 | 7,49 |
| **32** | (*Telmatochromis* cf. *temporalis*, *Lamprologus symoensi*) | 2,7534 | 1,4761 | 4,2367 | 2,5945 | 1,3651 | 3,9614 | 2,757 | 1,4458 | 4,1932 | 2,5808 | 1,3819 | 3,9672 |
| **33** | Eretmodini | 8,1704 | 4,1712 | 12,9724 | 7,75 | 3,9294 | 12,442 | 8,2695 | 4,978 | 12,7326 | 7,6449 | 3,9225 | 11,8982 |
| **34** | C-lineage (sensu Clabaut et al., 2005), Eretmodini | 24,8103 | 20,5758 | 29,3516 | 23,3698 | 19,9962 | 27,1276 | 24,6196 | 20,4644 | 29,2546 | 23,2242 | 19,5463 | 27,581 |
| **35** | Cyphotilapini | 15,5681 | 9,3915 | 21,3191 | 14,6823 | 8,9326 | 20,284 | 15,2739 | 9,56 | 21,1455 | 14,4163 | 9,89 | 19,5827 |
| **36** | Limnochromini | 11,7808 | 5,7262 | 18,1511 | 11,1836 | 5,3251 | 17,132 | 11,5522 | 5,812 | 17,9105 | 11,0085 | 5,5681 | 16,6599 |
| **37** | Perisodini | 6,5806 | 3,2081 | 10,2326 | 6,3867 | 3,1555 | 9,9179 | 6,6606 | 3,4588 | 10,5612 | 6,3479 | 3,2527 | 9,9063 |
| **38** | Cyprichromini | 11,2925 | 6,4384 | 16,1351 | 10,7553 | 6,3973 | 15,2368 | 11,1996 | 6,5435 | 16,2083 | 10,5442 | 6,3348 | 15,24 |
| **39** | 'benthopelagic LT clade' | 17,5571 | 13,3348 | 21,8438 | 16,636 | 13,397 | 20,1563 | 17,3533 | 13,1146 | 21,6271 | 16,4365 | 12,5583 | 20,582 |
| **40** | Ectodini | 15,1496 | 11,5774 | 18,7712 | 14,315 | 11,1197 | 17,4553 | 15,1566 | 11,6877 | 19,342 | 14,3092 | 11,244 | 17,5475 |
| **41** | 'Malagarasi-*Orthochromis*' | 12,4283 | 8,4658 | 16,3663 | 11,7021 | 8,135 | 15,2341 | 12,3878 | 8,5092 | 16,3513 | 11,6299 | 8,264 | 15,1649 |
| **42** | ('Malagarasi-*Orthochromis*', Haplochromini) | 20,0847 | 16,6039 | 23,7223 | 18,9739 | 16,1999 | 21,8362 | 19,9378 | 16,5407 | 23,5972 | 18,8302 | 15,8591 | 21,8836 |
| **43** | Haplochromini | 18,071 | 14,9841 | 21,4256 | 17,0929 | 14,5976 | 19,7878 | 17,9497 | 14,8019 | 21,2933 | 16,9554 | 14,2789 | 19,7972 |
| **44** | Haplochromini (excluding *Ctenochromis pectoralis*) | 16,7945 | 13,8821 | 19,8581 | 15,9 | 13,5858 | 18,3686 | 14,464 | 13,7312 | 19,7597 | 15,7637 | 13,2371 | 18,3448 |
| **45** | ('Congo-basin-Haplochromini', ('serranochromines-mt-lineage', 'LML-*Orthochromis*')) | 14,5582 | 11,8091 | 17,7123 | 13,7477 | 11,3102 | 16,2824 | 14,464 | 11,5893 | 17,3842 | 13,5894 | 11,299 | 16,1369 |
| **46** | ('serranochromines-mt-lineage sensu stricto', *Chetia welwitschi)* | 10,0346 | 7,6299 | 12,6355 | 9,5098 | 7,3292 | 11,7813 | 9,9719 | 7,5265 | 12,5215 | 9,3559 | 7,803 | 11,5518 |
| **47** | 'serrranochromines-mt-lineage sensu stricto' | 5,8997 | 3,966 | 7,848 | 5,6124 | 3,911 | 7,3686 | 5,8599 | 4,0049 | 7,8046 | 5,5229 | 3,7687 | 7,2666 |
| **48** | (*Serranochromis altus*, *Pharyngochromis* sp. "black bars") | 4,7265 | 2,8324 | 6,836 | 4,4868 | 2,6675 | 6,3268 | 4,6998 | 2,8141 | 6,6733 | 4,4127 | 2,5907 | 6,172 |
| **49** | ('*Orthochromis*' sp. 'Lufubu ', ('*Pseudocrenilabrus*-group', 'ocellated eggspot Haplochromini') | 16,4296 | 13,5617 | 19,4102 | 15,5595 | 13,2209 | 17,9248 | 16,3131 | 13,3009 | 19,2752 | 15,4209 | 12,9537 | 18,49 |
| **50** | *Pseudocrenilabrus*-group' | 12,8544 | 10,1499 | 15,6417 | 12,1683 | 9,8736 | 14,5597 | 12,7522 | 10,1759 | 15,6354 | 12,0906 | 9,5543 | 14,5788 |
| **51** | (*Pseudocrenilabrus multicolor*, *Pseudocrenilabrus nicholsi*) | 2,3915 | 1,1956 | 3,64 | 2,2824 | 1,2145 | 3,5239 | 2,3824 | 1,2345 | 3,6305 | 2,2443 | 1,1733 | 3,4046 |
| **52** | 'ocellated eggspot Haplochromini' | 15,329 | 12,6241 | 18,2121 | 14,5209 | 12,2646 | 16,8018 | 15,2318 | 12,4725 | 18,1675 | 14,3994 | 12,846 | 16,9525 |
| **53** | 'ocellated eggspot Haplochromini' excluding *Astatoreochromis* | 12,7502 | 10,447 | 15,3021 | 12,0436 | 10,11 | 14,1455 | 12,7186 | 10,2786 | 15,2292 | 12,0024 | 9,9315 | 14,2209 |
| **54** | (Tropheini, '*Haplochromis*' *vanheusdeni*) | 11,3771 | 8,9389 | 13,8055 | 10,7499 | 8,6169 | 13,48 | 11,375 | 8,9062 | 13,925 | 10,7197 | 8,5154 | 12,9782 |
| **55** | (*Tropheus moorii*, *Tropheus polli*) | 3,8029 | 1,7212 | 6,2277 | 3,6597 | 1,6774 | 5,9346 | 3,8238 | 1,6619 | 6,3225 | 3,6108 | 1,5892 | 5,7863 |
| **56** | Tropheini (excluding Tropheus moorii, Tropheus polli) | 7,8166 | 5,8352 | 9,9373 | 7,3717 | 5,5764 | 9,2484 | 7,8187 | 5,7598 | 9,9861 | 7,3674 | 5,4919 | 9,2534 |
| **57** | Tropheini | 9,4136 | 7,1866 | 11,7164 | 8,888 | 6,8788 | 10,9946 | 9,3894 | 7,1597 | 11,8104 | 8,8552 | 6,788 | 11,53 |
| **58** | (*Haplochromis demeusii*, *Haplochromis fasciatus*) | 5,8101 | 3,4079 | 8,2606 | 5,4126 | 3,3019 | 7,5636 | 5,7515 | 3,5011 | 8,31 | 5,398 | 3,2706 | 7,6009 |
| **59** | 'riverine & modern Haplochromini' | 10,192 | 8,808 | 12,4682 | 9,6233 | 7,8366 | 11,5399 | 10,1922 | 8,187 | 12,4634 | 9,6069 | 7,6779 | 11,5291 |
| **60** | (LVRS, *Haplochromis stappersii*) | 1,0644 | 0,5325 | 1,658 | 1,0077 | 0,5292 | 1,5588 | 1,0591 | 0,5359 | 1,6457 | 0,9943 | 0,508 | 1,5245 |
| **61** | Lake Victoria Region Superflock (LVRS) | 0,3337 | 0,1273 | 0,5831 | 0,3154 | 0,1181 | 0,5411 | 0,3328 | 0,1253 | 0,5699 | 0,3121 | 0,119 | 0,5388 |
| **62** | ('riverine & modern Haplochromini', Lake Malawi species flock) | 10,7921 | 8,5851 | 13,1092 | 10,1959 | 8,342 | 12,161 | 10,7879 | 8,5261 | 13,839 | 10,1696 | 8,2227 | 12,1754 |
| **63** | Lake Malawi species flock | 4,3332 | 3,11 | 5,7072 | 4,0819 | 3,314 | 5,3534 | 4,2951 | 3,554 | 5,5836 | 4,0875 | 2,9995 | 5,3602 |
| **64** | 'primarily Mbuna taxa' | 0,6457 | 0,427 | 0,8886 | 0,6103 | 0,4077 | 0,8312 | 0,6391 | 0,4118 | 0,8812 | 0,6055 | 0,4102 | 0,8317 |
| **65** | 'primarily LM Sanddweller' | 0,7464 | 0,4797 | 1,423 | 0,7076 | 0,4494 | 0,9854 | 0,7403 | 0,4733 | 1,318 | 0,7034 | 0,4586 | 0,9744 |
|  |  |  |  |  |  |  |  |  |  |  |  |  |  |

| **Node corresponding numbers in Fig 2.:** | **Comment to clade (MRCA):** | **Set 9** | **95 % HPD:** |  | **Set 10** | **95 % HPD:** |  | **Set 11** | **95 % HPD:** |  | **Set 12** | **95 % HPD:** |  |
| --- | --- | --- | --- | --- | --- | --- | --- | --- | --- | --- | --- | --- | --- |
|  |  | **Mean age** | **Min** | **Max** | **Mean age** | **Min** | **Max** | **Mean age** | **Min** | **Max** | **Mean age** | **Min** | **Max** |
| **1** | Cichlidae | 103,0894 | 84,2172 | 122,3057 | 92,9711 | 85,3868 | 102,114 | 104,4308 | 83,7692 | 126,5378 | 91,7119 | 84,6398 | 96,9757 |
| **2** | (Cichlinae, Pseudocrenilabrinae) | 90,9442 | 77,1613 | 105,8514 | 84,4227 | 75,6592 | 93,2742 | 91,489 | 76,871 | 107,8344 | 79,3897 | 67,9824 | 87,9394 |
| **3** | Cichlinae | 78,397 | 67,8142 | 90,1985 | 73,9588 | 66,1469 | 82,1858 | 78,714 | 67,852 | 90,736 | 72,4224 | 61,5803 | 80,4004 |
| **4** | Cichlasomatini 'Andinoacarines' | 18,7745 | 11,3166 | 26,7701 | 17,6835 | 10,7581 | 24,5567 | 18,386 | 11,2159 | 26,3605 | 14,3319 | 6,6226 | 23,2352 |
| **5** | 'mesoamerican' Heroini | 34,79 | 28,4403 | 40,6937 | 34,1032 | 27,6064 | 39,6574 | 34,9492 | 28,3825 | 41,1708 | - | - | - |
| **6** | Cichliasomatini | 51,6042 | 42,3878 | 61,958 | 49,3781 | 40,7845 | 57,2408 | 51,5738 | 41,7137 | 61,2391 | 47,1511 | 36,104 | 58,6055 |
| **7** | Heroini | 54,3294 | 48,1799 | 61,1562 | 52,5954 | 47,5397 | 57,9581 | 54,4907 | 48,3662 | 61,778 | 52,8728 | 45,9849 | 60,3204 |
| **8** | (Heroini, Cichlasomatini) | 62,9255 | 54,9753 | 71,5053 | 60,0834 | 53,8518 | 66,7033 | 63,0742 | 54,8284 | 72,267 | 59,2129 | 51,177 | 67,8409 |
| **9** | Pseudocrenilabrinae | 64,9288 | 52,515 | 77,7699 | 61,5123 | 51,261 | 71,9159 | 65,1465 | 52,3598 | 78,1283 | 62,838 | 48,1732 | 74,1302 |
| **10** | Haplotilapiines (sensu Schliewen & Stiassny, 2003) | 49,2591 | 39,6156 | 60,18 | 46,9646 | 38,1797 | 55,8188 | 49,3542 | 39,2055 | 59,8315 | 50,6943 | 38,1498 | 61,134 |
| **11** | (Coptodonini, (Pelmatolapiini, (Oreochromini, Austrotilapiines))) | 38,0351 | 31,1966 | 44,9841 | 36,2922 | 30,4892 | 42,4248 | 38,2441 | 31,3458 | 45,4255 | 43,9208 | 33,94 | 52,3033 |
| **12** | Oreochromini | 24,2871 | 17,8951 | 31,3173 | 23,1816 | 17,3265 | 29,4496 | 24,198 | 17,6725 | 31,791 | 19,7151 | 10,3178 | 28,1502 |
| **13** | Oreochromini (excluding *Danakilia*) | 21,0441 | 15,3175 | 27,3321 | 20,1775 | 14,9789 | 25,9667 | 21,002 | 15,3031 | 27,796 | 5,7093 | 8,3983 | 20,7019 |
| **14** | Australotilapiines (sensu Schwarzer et al., 2009) | 33,8965 | 28,1514 | 39,9444 | 32,3635 | 27,477 | 37,429 | 34,1275 | 28,8639 | 41,3927 | - | - | - |
| **15** | Steatocranini | 12,837 | 8,2382 | 17,9584 | 12,2936 | 7,85 | 17,164 | 12,9896 | 8,4692 | 18,2546 | 16,8605 | 7,6385 | 23,51 |
| **16** | (Steatocranini, East African Radiation) | 32,4148 | 26,646 | 38,1358 | 30,9167 | 26,1264 | 35,7407 | 32,668 | 26,8568 | 38,6266 | - | - | - |
| **17** | East African Radiation (EAR) | 30,3584 | 25,1313 | 35,8706 | 28,9435 | 24,4583 | 33,5099 | 30,6151 | 25,1916 | 36,1727 | 35,6133 | 26,567 | 41,7713 |
| **18** | Trematocarini | 17,0655 | 12,6069 | 21,9876 | 16,1574 | 11,7493 | 20,5954 | 17,1103 | 12,5457 | 22,1099 | 18,5358 | 9,7349 | 24,5185 |
| **19** | (Bathybatini, Trematocarini) | 25,5861 | 20,7445 | 30,7598 | 24,4265 | 20,2325 | 28,7512 | 25,8627 | 20,7471 | 30,9219 | 29,4275 | 21,144 | 35,5252 |
| **20** | Bathybatini (including *Hemibates*) | 21,6883 | 16,5823 | 27,323 | 20,686 | 16,2412 | 25,4915 | 21,9092 | 17,364 | 27,3559 | 25,1332 | 16,4639 | 31,27 |
| **21** | *Bathybates* | 13,9754 | 9,727 | 18,5996 | 13,2997 | 9,7057 | 17,1438 | 14,0175 | 9,8812 | 18,6319 | 17,7982 | 10,2503 | 23,756 |
| **22** | *Bathybates* (excluding *Bathybates minor*) | 8,6587 | 5,8953 | 11,6467 | 8,2629 | 5,7 | 10,8918 | 8,6658 | 5,9245 | 11,6504 | 12,2565 | 10,2503 | 23,756 |
| **23** | East African Radiation (EAR) excluding Boulengerochromini | 29,3097 | 24,3513 | 34,7278 | 27,9383 | 23,6294 | 32,3576 | 30,6151 | 24,3062 | 34,899 | 33,9088 | 25,351 | 39,7257 |
| **24** | MVhL-clade (sensu Takahashi et al., 2001) | 25,0597 | 20,7862 | 29,4737 | 23,8757 | 20,2473 | 27,7393 | 25,1493 | 20,5689 | 29,6529 | 29,349 | 21,8478 | 33,8616 |
| **25** | Lamprologini | 16,1284 | 12,5622 | 20,11 | 15,4028 | 12,668 | 18,8329 | 16,1342 | 12,3659 | 19,8831 | 20,7814 | 12,9575 | 26,4286 |
| **26** | 'Lacustrine lamprologines' | 15,0783 | 11,6994 | 18,711 | 14,3747 | 11,1516 | 17,6503 | 15,1209 | 11,5399 | 18,7281 | - | - | - |
| **27** | 'Non-ossfied lacustrine Lamprologines' | 13,2124 | 9,9149 | 16,5613 | 12,5947 | 9,5224 | 15,7504 | 13,2127 | 9,9925 | 16,7494 | 13,3208 | 5,9493 | 18,7354 |
| **28** | 'Ossified lamprologines' | 11,1887 | 7,5749 | 15,329 | 10,7487 | 7,3257 | 14,3744 | 11,2136 | 7,5603 | 14,969 | 14,6062 | 7,8935 | 18,8621 |
| **29** | 'Lower Congo *Lamprologus* clade' | 6,9899 | 4,5476 | 9,6322 | 6,5901 | 4,2614 | 9,1144 | 6,9925 | 4,619 | 9,6873 | 10,515 | 4,5405 | 14,3436 |
| **30** | (*Telmatochromis* sp. 'Lufubu', *Neolamprologus brichardi*) | 4,5775 | 2,4451 | 6,7943 | 4,3484 | 2,4351 | 6,4688 | 4,5393 | 2,3873 | 6,7584 | 6,8675 | 2,102 | 8,9181 |
| **31** | (*Lamprologus werneri*, (*Telmatochromis* cf. *temporalis*, *Lamprologus symoensi*)) | 5,3669 | 3,4858 | 7,3703 | 5,1229 | 3,3633 | 7,703 | 5,3672 | 3,4219 | 7,3889 | 6,8076 | 2,3204 | 8,6367 |
| **32** | (*Telmatochromis* cf. *temporalis*, *Lamprologus symoensi*) | 2,6999 | 1,3861 | 4,139 | 2,5745 | 1,3399 | 3,9966 | 2,6881 | 1,4221 | 4,1516 | 4,9852 | 1,1172 | 6,2476 |
| **33** | Eretmodini | 8,0403 | 3,9862 | 12,6206 | 7,7238 | 3,9151 | 11,7866 | 7,9973 | 3,8509 | 12,5243 | 10,7552 | 2,7882 | 17,726 |
| **34** | C-lineage (sensu Clabaut et al., 2005), Eretmodini | 24,2213 | 19,9589 | 28,4377 | 23,0672 | 19,3815 | 26,645 | 24,2756 | 19,8406 | 28,589 | 27,4879 | 20,4455 | 31,6814 |
| **35** | Cyphotilapini | 15,3929 | 9,23 | 21,95 | 14,4864 | 8,797 | 20,2498 | 15,2344 | 9,2187 | 20,9964 | 14,3252 | 3,6857 | 22,1086 |
| **36** | Limnochromini | 11,3748 | 5,4616 | 17,7866 | 11,0501 | 5,7134 | 16,6835 | 11,4348 | 5,8262 | 17,1702 | 12,9789 | 3,922 | 19,4793 |
| **37** | Perisodini | 6,68 | 3,3331 | 10,3511 | 6,2817 | 3,1382 | 9,7931 | 6,5094 | 3,1905 | 10,3443 | 8,9426 | 2,3315 | 13,1778 |
| **38** | Cyprichromini | 11,1138 | 6,5369 | 15,9432 |  | 6,2137 | 14,6532 | 11,1859 | 6,444 | 15,9377 | 10,5258 | 3,1376 | 15,3797 |
| **39** | 'benthopelagic LT clade' | 17,265 | 13,4168 | 21,2159 | 16,333 | 12,721 | 20,1247 | 17,1849 | 13,576 | 21,2478 | 17,7439 | 9,8505 | 22,6888 |
| **40** | Ectodini | 14,9568 | 11,5784 | 18,5632 | 14,1557 | 11,624 | 17,2345 | 14,8876 | 11,4888 | 18,4781 | 17,7932 | 10,5881 | 22,5974 |
| **41** | 'Malagarasi-*Orthochromis*' | 12,2882 | 8,5261 | 16,1541 | 11,6293 | 8,411 | 15,1348 | 12,1698 | 8,4335 | 16,1352 | 16,9094 | 9,7775 | 21,1214 |
| **42** | ('Malagarasi-*Orthochromis*', Haplochromini) | 19,7464 | 16,4751 | 23,1472 | 18,7661 | 15,8988 | 21,6539 | 19,6274 | 16,209 | 22,9615 | - | - | - |
| **43** | Haplochromini | 17,7617 | 14,7823 | 20,9498 | 16,8686 | 14,254 | 19,6401 | 17,6855 | 14,5768 | 20,7659 | 23,618 | 17,4043 | 27,889 |
| **44** | Haplochromini (excluding *Ctenochromis pectoralis*) | 16,5188 | 13,7466 | 19,5054 | 15,6877 | 13,2595 | 18,2269 | 16,4615 | 13,5737 | 19,3872 | - | - | - |
| **45** | ('Congo-basin-Haplochromini', ('serranochromines-mt-lineage', 'LML-*Orthochromis*')) | 14,332 | 11,4284 | 17,1389 | 13,5677 | 11,912 | 16,928 | 14,2942 | 11,4708 | 17,1256 | 20,4375 | 14,241 | 23,8682 |
| **46** | ('serranochromines-mt-lineage sensu stricto', *Chetia welwitschi)* | 9,8881 | 7,4986 | 12,3479 | 9,3661 | 7,1741 | 11,7743 | 9,9042 | 7,3958 | 12,472 | 15,0749 | 8,9747 | 18,3729 |
| **47** | 'serrranochromines-mt-lineage sensu stricto' | 5,8328 | 4,57 | 7,6749 | 5,5253 | 3,6817 | 7,3522 | 5,8659 | 3,9804 | 7,9316 | 12,8071 | 7,566 | 15,8782 |
| **48** | (*Serranochromis altus*, *Pharyngochromis* sp. "black bars") | 4,6659 | 2,847 | 6,6666 | 4,4185 | 2,5414 | 6,3045 | 4,7009 | 2,7185 | 6,7043 | - | - | - |
| **49** | ('*Orthochromis*' sp. 'Lufubu ', ('*Pseudocrenilabrus*-group', 'ocellated eggspot Haplochromini') | 16,1571 | 13,4707 | 19,1633 | 15,343 | 12,9636 | 17,9027 | 16,102 | 13,2584 | 18,9773 | - | - | - |
| **50** | *Pseudocrenilabrus*-group' | 12,5872 | 9,8474 | 15,3283 | 11,9427 | 9,4583 | 14,3389 | 12,5698 | 9,856 | 15,3691 | 18,1664 | 12,109 | 21,7649 |
| **51** | (*Pseudocrenilabrus multicolor*, *Pseudocrenilabrus nicholsi*) | 2,3246 | 1,2281 | 3,5768 | 2,2199 | 1,1794 | 3,3611 | 2,3371 | 1,1936 | 3,5831 | 5,1759 | 1,2697 | 6,5882 |
| **52** | 'ocellated eggspot Haplochromini' | 15,0724 | 12,4626 | 17,9367 | 14,321 | 11,9422 | 16,7346 | 15,0387 | 12,3002 | 17,8022 | - | - | - |
| **53** | 'ocellated eggspot Haplochromini' excluding *Astatoreochromis* | 12,5376 | 10,2573 | 15,161 | 11,9378 | 9,7973 | 14,303 | 12,5841 | 10,1627 | 15,1033 | 20,6677 | 14,5543 | 23,9388 |
| **54** | (Tropheini, '*Haplochromis*' *vanheusdeni*) | 11,1891 | 8,8397 | 13,7015 | 10,6505 | 8,3977 | 12,8232 | 11,2415 | 8,7948 | 13,6789 | - | - | - |
| **55** | (*Tropheus moorii*, *Tropheus polli*) | 3,7521 | 1,6923 | 6,1286 | 3,6101 | 1,6251 | 5,9388 | 3,752 | 1,6935 | 6,1618 | 4,9781 | 0,6964 | 7,1259 |
| **56** | Tropheini (excluding Tropheus moorii, Tropheus polli) | 7,647 | 5,7265 | 9,7197 | 7,2654 | 5,4249 | 9,1927 | 7,7435 | 5,7513 | 9,742 | 9,7501 | 4,2976 | 12,8509 |
| **57** | Tropheini | 9,215 | 7,542 | 11,5463 | 8,7677 | 6,7038 | 10,8992 | 9,3076 | 7,1226 | 11,5811 | 12,1765 | 5,6067 | 16,3577 |
| **58** | (*Haplochromis demeusii*, *Haplochromis fasciatus*) | 5,6898 | 3,4738 | 8,351 | 5,3254 | 3,2271 | 7,4785 | 5,6868 | 3,4082 | 8,594 | 11,3036 | 4,6044 | 14,9515 |
| **59** | 'riverine & modern Haplochromini' | 10,0422 | 8,223 | 12,2579 | 9,5344 | 7,6322 | 11,3613 | 10,0924 | 7,8671 | 12,286 | 16,8463 | 11,351 | 19,7489 |
| **60** | (LVRS, *Haplochromis stappersii*) | 1,0419 | 0,5327 | 1,6261 | 0,9852 | 0,5164 | 1,5251 | 1,0507 | 0,5315 | 1,6301 | 4,2276 | 0,9647 | 4,8764 |
| **61** | Lake Victoria Region Superflock (LVRS) | 0,3279 | 0,1236 | 0,5667 | 0,3105 | 0,1202 | 0,5379 | 0,3293 | 0,1293 | 0,5805 | 2,8209 | 0,2768 | 2,7147 |
| **62** | ('riverine & modern Haplochromini', Lake Malawi species flock) | 10,6316 | 8,5427 | 12,8718 | 10,0992 | 8,1561 | 12,9 | 10,679 | 8,4716 | 13,89 | 18,2685 | 12,3491 | 21,2229 |
| **63** | Lake Malawi species flock | 4,2656 | 3,538 | 5,6052 | 4,0541 | 2,9227 | 5,2641 | 4,2738 | 3,184 | 5,5319 | 9,0419 | 4,1586 | 11,4273 |
| **64** | 'primarily Mbuna taxa' | 0,6354 | 0,4169 | 0,8807 | 0,6055 | 0,408 | 0,832 | 0,6371 | 0,416 | 0,8798 | 3,4416 | 0,7778 | 3,3607 |
| **65** | 'primarily LM Sanddweller' | 0,7312 | 0,4714 | 1,261 | 0,6972 | 0,4577 | 0,9699 | 0,7354 | 0,4771 | 1,349 | 3,5186 | 0,6514 | 3,712 |
|  |  |  |  |  |  |  |  |  |  |  |  |  |  |

| **Node corresponding numbers in Fig 2.:** | **Comment to clade (MRCA):** | **Set 13** | **95 % HPD:** |  | **Set 14** |  |  | **Set 15** | **95 % HPD:** |  | **Set 16** | **95 % HPD:** |  |
| --- | --- | --- | --- | --- | --- | --- | --- | --- | --- | --- | --- | --- | --- |
|  |  | **Mean age** | **Min** | **Max** | **Mean age** | **Min** | **Max** | **Mean age** | **Min** | **Max** | **Mean age** | **Min** | **Max** |
| **1** | Cichlidae | 93,4734 | 85,4063 | 102,9087 | 92,108 | 85,2362 | 100,7099 | 96,0404 | 86,694 | 107,9671 | 104,0297 | 84,4337 | 125,2984 |
| **2** | (Cichlinae, Pseudocrenilabrinae) | 85,1187 | 76,2481 | 94,6985 | 82,9929 | 74,494 | 92,0815 | 87,8469 | 77,9275 | 99,2532 | 91,526 | 76,8573 | 107,8149 |
| **3** | Cichlinae | 74,8662 | 67,7 | 83,48 | 72,5068 | 64,2306 | 80,5734 | 77,1871 | 67,8104 | 86,8294 | 78,7869 | 67,2298 | 91,8313 |
| **4** | Cichlasomatini 'Andinoacarines' | 17,7859 | 11,1443 | 24,9764 | 17,3223 | 10,6906 | 24,4194 | 18,9913 | 11,7717 | 26,4599 | 18,6481 | 10,8057 | 26,6901 |
| **5** | 'mesoamerican' Heroini | 34,3164 | 28,132 | 39,9891 | 33,8509 | 27,4699 | 39,7193 | 35,604 | 29,1482 | 40,5638 | 35,1332 | 28,826 | 41,0733 |
| **6** | Cichliasomatini | 50,8111 | 2,9803 | 59,1 | 48,344 | 40,2993 | 56,3371 | 51,8976 | 43,2124 | 60,8086 | 51,9173 | 41,9817 | 61,8856 |
| **7** | Heroini | 53,1822 | 47,9342 | 59,1735 | 51,9202 | 47,1204 | 57,1522 | 54,2341 | 48,304 | 60,3285 | 54,6044 | 48,923 | 61,5492 |
| **8** | (Heroini, Cichlasomatini) | 61,0349 | 54,7023 | 68,1745 | 58,9488 | 52,8794 | 65,6931 | 62,5772 | 55,3315 | 70,3756 | 63,169 | 54,608 | 72,3819 |
| **9** | Pseudocrenilabrinae | 61,2562 | 51,368 | 72,1997 | 59,7755 | 49,4516 | 70,1536 | 65,2975 | 53,7682 | 77,25 | 64,3226 | 51,5073 | 78,5413 |
| **10** | Haplotilapiines (sensu Schliewen & Stiassny, 2003) | 47,2722 | 38,194 | 56,1638 | 45,7331 | 37,4267 | 54,4448 | 50,357 | 40,811 | 60,1859 | 48,8242 | 38,9077 | 59,974 |
| **11** | (Coptodonini, (Pelmatolapiini, (Oreochromini, Austrotilapiines))) | 36,6851 | 30,394 | 42,8192 | 35,4626 | 29,7122 | 41,3681 | 39,4615 | 32,9449 | 46,4826 | 37,9887 | 31,0154 | 44,9783 |
| **12** | Oreochromini | 23,4479 | 17,2402 | 29,6741 | 22,5864 | 16,7085 | 28,981 | 25,3804 | 18,5007 | 32,5144 | 24,1776 | 17,6234 | 31,1064 |
| **13** | Oreochromini (excluding *Danakilia*) | 20,355 | 14,864 | 25,9039 | 19,5615 | 14,5555 | 25,2943 | 21,9884 | 15,9864 | 28,4825 | 20,9735 | 15,2176 | 27,0583 |
| **14** | Australotilapiines (sensu Schwarzer et al., 2009) | 32,7319 | 27,6362 | 38,282 | 31,6396 | 26,5795 | 36,6268 | 35,2139 | 29,7746 | 41,3737 | 33,8349 | 27,874 | 39,8065 |
| **15** | Steatocranini | 12,5112 | 8,3534 | 17,3963 | 12,0562 | 8,0271 | 16,5187 | 13,3058 | 8,7015 | 18,3112 | 13,0202 | 8,2734 | 18,1212 |
| **16** | (Steatocranini, East African Radiation) | 31,3203 | 26,3649 | 36,552 | 30,2849 | 25,4179 | 35,1269 | 33,7063 | 28,1544 | 39,4891 | 32,4264 | 26,766 | 38,3846 |
| **17** | East African Radiation (EAR) | 29,3451 | 24,5411 | 34,3 | 28,3896 | 23,8322 | 32,8671 | 31,595 | 26,4402 | 36,989 | 30,4017 | 24,9816 | 35,8718 |
| **18** | Trematocarini | 16,4598 | 12,1245 | 21,266 | 16,0841 | 11,6353 | 20,5647 | 17,6933 | 13,1628 | 22,7063 | 16,8709 | 11,7898 | 21,9472 |
| **19** | (Bathybatini, Trematocarini) | 24,8412 | 20,421 | 29,4777 | 23,9837 | 19,586 | 28,3222 | 26,7349 | 21,8452 | 31,8235 | 25,6884 | 20,7719 | 31,0823 |
| **20** | Bathybatini (including *Hemibates*) | 21,0637 | 16,3355 | 25,8215 | 20,293 | 15,5687 | 24,791 | 22,6878 | 17,6542 | 28,1338 | 21,851 | 16,6486 | 27,2003 |
| **21** | *Bathybates* | 13,4674 | 9,6191 | 17,7304 | 12,971 | 9,922 | 16,8239 | 14,4959 | 10,1804 | 19,465 | 13,9574 | 9,5326 | 18,4511 |
| **22** | *Bathybates* (excluding *Bathybates minor*) | 8,3133 | 5,7148 | 11,738 | 8,0194 | 5,5348 | 10,723 | 8,9221 | 6,1202 | 11,9097 | 8,6388 | 5,9111 | 11,6568 |
| **23** | East African Radiation (EAR) excluding Boulengerochromini | 28,3206 | 23,7862 | 32,8825 | 27,3934 | 22,9945 | 31,755 | 30,4877 | 25,4035 | 35,6983 | 29,3395 | 24,1429 | 34,67 |
| **24** | MVhL-clade (sensu Takahashi et al., 2001) | 24,1437 | 20,3924 | 28,446 | 23,2877 | 19,5866 | 27,0851 | 25,9342 | 21,6804 | 30,4668 | 25,0253 | 20,5144 | 29,5995 |
| **25** | Lamprologini | 15,4116 | 12,1175 | 18,9306 | 14,9147 | 11,7986 | 18,2658 | 16,7679 | 13,1643 | 20,7073 | 16,0776 | 12,448 | 19,9053 |
| **26** | 'Lacustrine lamprologines' | 14,4122 | 11,4036 | 17,8468 | 13,9467 | 10,8704 | 17,0547 | 15,6558 | 12,2585 | 19,5161 | 15,0148 | 11,5306 | 18,6825 |
| **27** | 'Non-ossfied lacustrine Lamprologines' | 12,6344 | 9,7534 | 15,9359 | 12,2089 | 9,4505 | 15,2291 | 13,7366 | 10,505 | 17,3477 | 13,1577 | 9,8621 | 16,6146 |
| **28** | 'Ossified lamprologines' | 10,7179 | 7,3684 | 14,1885 | 10,3075 | 7,0108 | 13,6036 | 11,6964 | 7,865 | 15,7618 | 11,1145 | 7,2229 | 14,9217 |
| **29** | 'Lower Congo *Lamprologus* clade' | 6,6728 | 4,5078 | 9,1579 | 6,469 | 4,2613 | 8,8665 | 7,1923 | 4,7472 | 9,8842 | 6,9055 | 4,4706 | 9,4773 |
| **30** | (*Telmatochromis* sp. 'Lufubu', *Neolamprologus brichardi*) | 4,4199 | 2,452 | 6,4931 | 4,2785 | 2,3494 | 6,3988 | 4,7749 | 2,6074 | 7,852 | 4,5543 | 2,3937 | 6,7216 |
| **31** | (*Lamprologus werneri*, (*Telmatochromis* cf. *temporalis*, *Lamprologus symoensi*)) | 5,1277 | 3,4229 | 6,983 | 5,0082 | 3,2747 | 6,8268 | 5,5812 | 3,5725 | 7,6737 | 5,3527 | 3,4722 | 7,3833 |
| **32** | (*Telmatochromis* cf. *temporalis*, *Lamprologus symoensi*) | 2,5601 | 1,3021 | 3,8754 | 2,5245 | 1,2752 | 3,8012 | 2,7948 | 1,5127 | 4,2907 | 2,683 | 1,4681 | 4,1349 |
| **33** | Eretmodini | 7,74 | 3,8798 | 11,9258 | 7,4597 | 3,667 | 11,5411 | 8,2341 | 3,9299 | 12,756 | 8,1466 | 4,1244 | 12,667 |
| **34** | C-lineage (sensu Clabaut et al., 2005), Eretmodini | 23,3065 | 19,6565 | 27,632 | 22,4866 | 18,9275 | 26,1642 | 25,464 | 21,315 | 29,5321 | 24,1536 | 19,9622 | 28,7574 |
| **35** | Cyphotilapini | 14,8152 | 9,875 | 20,372 | 14,1648 | 8,4048 | 19,131 | 15,7469 | 9,6567 | 21,3721 | 14,7908 | 8,6089 | 20,4742 |
| **36** | Limnochromini | 11,1454 | 5,4206 | 16,5889 | 10,4673 | 5,0228 | 15,9866 | 11,95 | 5,8224 | 17,9158 | 11,3264 | 5,609 | 17,0563 |
| **37** | Perisodini | 6,3141 | 3,2945 | 9,7743 | 5,9889 | 3,185 | 9,2393 | 6,7606 | 3,301 | 10,8215 | 6,5066 | 3,1466 | 10,2155 |
| **38** | Cyprichromini | 10,6407 | 6,3983 | 15,186 | 10,2765 | 5,8686 | 14,4745 | 11,3036 | 6,5112 | 15,9119 | 10,8838 | 6,2977 | 15,5282 |
| **39** | 'benthopelagic LT clade' | 16,3922 | 12,7398 | 20,2132 | 16,0528 | 12,4607 | 19,5154 | 17,6246 | 13,647 | 21,6662 | 16,9921 | 12,8849 | 21,0508 |
| **40** | Ectodini | 14,374 | 11,1473 | 17,7026 | 13,8768 | 10,9074 | 17,104 | 15,29 | 11,9354 | 18,9039 | 14,8591 | 11,2674 | 18,4901 |
| **41** | 'Malagarasi-*Orthochromis*' | 11,611 | 8,1123 | 15,2764 | 11,2644 | 7,9687 | 14,6677 | 12,5195 | 8,6654 | 16,2451 | 12,258 | 8,5885 | 16,1053 |
| **42** | ('Malagarasi-*Orthochromis*', Haplochromini) | 18,9424 | 16,347 | 21,8402 | 18,2059 | 15,2881 | 21,0708 | 20,2476 | 17,924 | 23,7922 | 19,6337 | 16,3059 | 23,2943 |
| **43** | Haplochromini | 17,0243 | 14,3797 | 19,7609 | 16,3597 | 13,6824 | 18,9916 | 18,2025 | 15,1853 | 21,3321 | 17,7458 | 14,6034 | 21,1154 |
| **44** | Haplochromini (excluding *Ctenochromis pectoralis*) | 15,839 | 13,3581 | 18,3743 | 15,2065 | 12,7668 | 17,7082 | 16,9269 | 14,1265 | 19,9313 | 16,522 | 13,6184 | 19,6819 |
| **45** | ('Congo-basin-Haplochromini', ('serranochromines-mt-lineage', 'LML-*Orthochromis*')) | 13,7361 | 11,2599 | 16,3209 | 13,137 | 10,6682 | 15,5461 | 14,6577 | 11,8674 | 17,5129 | 14,3025 | 11,3844 | 17,3072 |
| **46** | ('serranochromines-mt-lineage sensu stricto', *Chetia welwitschi)* | 9,449 | 7,2165 | 11,7809 | 9,0791 | 6,9368 | 11,347 | 10,1737 | 7,7893 | 12,7693 | 9,88071 | 7,447 | 12,4748 |
| **47** | 'serrranochromines-mt-lineage sensu stricto' | 5,5872 | 3,9033 | 7,4133 | 5,3628 | 3,7449 | 7,159 | 6,0131 | 4,2416 | 7,9578 | 5,8455 | 3,9778 | 7,8031 |
| **48** | (*Serranochromis altus*, *Pharyngochromis* sp. "black bars") | 4,494 | 2,7461 | 6,3392 | 4,2939 | 2,5672 | 6,1037 | 4,8315 | 2,9671 | 6,7587 | 4,6891 | 2,8036 | 6,6827 |
| **49** | ('*Orthochromis*' sp. 'Lufubu ', ('*Pseudocrenilabrus*-group', 'ocellated eggspot Haplochromini') | 15,0508 | 12,6105 | 17,4934 | 14,8739 | 12,4853 | 17,3453 | 16,879 | 13,3465 | 18,9591 | 16,1588 | 13,2512 | 19,2094 |
| **50** | *Pseudocrenilabrus*-group' | 12,06 | 9,6827 | 14,5452 | 11,58 | 9,2509 | 13,9762 | 12,9296 | 10,233 | 15,6808 | 12,6255 | 9,811 | 15,3792 |
| **51** | (*Pseudocrenilabrus multicolor*, *Pseudocrenilabrus nicholsi*) | 2,2656 | 1,1961 | 3,4417 | 2,1523 | 1,1121 | 3,3497 | 2,4348 | 1,2927 | 3,7191 | 2,3363 | 1,2012 | 3,5559 |
| **52** | 'ocellated eggspot Haplochromini' | 14,4569 | 12,1188 | 16,958 | 13,8704 | 11,5597 | 16,275 | 15,4583 | 12,7356 | 18,2097 | 15,0892 | 12,3075 | 18,393 |
| **53** | 'ocellated eggspot Haplochromini' excluding *Astatoreochromis* | 12,0377 | 9,9727 | 14,237 | 11,5911 | 9,593 | 13,7813 | 12,8838 | 10,4652 | 15,3644 | 12,583 | 10,1875 | 15,1832 |
| **54** | (Tropheini, '*Haplochromis*' *vanheusdeni*) | 10,75 | 8,5919 | 13,74 | 10,3751 | 8,26 | 12,5213 | 11,5088 | 9,659 | 14,928 | 11,2181 | 8,8287 | 13,8062 |
| **55** | (*Tropheus moorii*, *Tropheus polli*) | 3,5856 | 1,6117 | 5,8241 | 3,498 | 1,5795 | 5,8268 | 3,8477 | 1,706 | 6,2274 | 3,7048 | 1,6169 | 6,0446 |
| **56** | Tropheini (excluding Tropheus moorii, Tropheus polli) | 7,3463 | 5,5598 | 9,1711 | 7,1785 | 6,6951 | 10,7204 | 7,8971 | 5,8606 | 9,9668 | 7,6732 | 5,7038 | 9,6821 |
| **57** | Tropheini | 8,8599 | 6,8625 | 10,9827 | 8,63 | 8,26 | 12,5213 | 9,4976 | 7,3674 | 11,9462 | 9,2415 | 7,329 | 11,5365 |
| **58** | (*Haplochromis demeusii*, *Haplochromis fasciatus*) | 5,4322 | 3,3271 | 7,5675 | 5,2664 | 3,1579 | 7,3319 | 5,8525 | 3,6061 | 8,871 | 5,69 | 3,2981 | 7,9598 |
| **59** | 'riverine & modern Haplochromini' | 9,612 | 7,791 | 11,56 | 9,2803 | 7,3562 | 11,2116 | 10,3138 | 8,2463 | 12,5611 | 10,0861 | 7,938 | 12,4122 |
| **60** | (LVRS, *Haplochromis stappersii*) | 1,0084 | 0,5254 | 1,5831 | 0,9716 | 0,5196 | 1,5213 | 1,763 | 0,5664 | 1,6829 | 1,0403 | 0,5287 | 1,6169 |
| **61** | Lake Victoria Region Superflock (LVRS) | 0,3152 | 0,1157 | 0,5469 | 0,3045 | 0,1197 | 0,5296 | 0,3381 | 0,1315 | 0,5862 | 0,3269 | 0,1251 | 0,5646 |
| **62** | ('riverine & modern Haplochromini', Lake Malawi species flock) | 10,1767 | 8,2627 | 12,1443 | 9,8274 | 7,92 | 11,8562 | 10,9172 | 8,7594 | 13,2041 | 10,6771 | 8,4864 | 13,0764 |
| **63** | Lake Malawi species flock | 4,1222 | 2,9624 | 5,3716 | 3,967 | 2,8262 | 5,1609 | 4,3668 | 3,947 | 5,7055 | 4,2387 | 3,185 | 5,5637 |
| **64** | 'primarily Mbuna taxa' | 0,6102 | 0,4047 | 0,8339 | 0,588 | 0,3938 | 0,8125 | 0,6548 | 0,4408 | 0,909 | 0,6331 | 0,4094 | 0,874 |
| **65** | 'primarily LM Sanddweller' | 0,7068 | 0,4713 | 0,9899 | 0,6798 | 0,434 | 0,9518 | 0,7539 | 0,4822 | 1,616 | 0,7318 | 0,4664 | 1,0237 |
| **F (Barombi Mbo radiation)** |  |  |  |  |  |  |  | 1.7612 | 1.1716 | 2.4064 |  |  |  |

| **Node corresponding numbers in Fig 2.:** | **Comment to clade (MRCA):** | **Set 17** | **95 % HPD:** |  | **Set 18** | **95 % HPD:** |  |
| --- | --- | --- | --- | --- | --- | --- | --- |
|  |  | **Mean age** | **Min** | **Max** | **Mean age** | **Min** | **Max** |
| **1** | Cichlidae | 93,1397 | 85,4354 | 102,3311 | 111,7279 | 89,7237 | 136,9253 |
| **2** | (Cichlinae, Pseudocrenilabrinae) | 85,0008 | 76,495 | 94,2449 | 99,1959 | 80,6552 | 121,4571 |
| **3** | Cichlinae | 74,5431 | 66,6023 | 82,726 | 83,117 | 67,9816 | 100,4426 |
| **4** | Cichlasomatini 'Andinoacarines' | 18,5483 | 11,5622 | 25,8828 | 11,2236 | 4,6033 | 18,6962 |
| **5** | 'mesoamerican' Heroini | 34,3556 | 28,3262 | 39,9881 | 26,1645 | 17,8869 | 34,6349 |
| **6** | Cichliasomatini | 51,1057 | 43,6204 | 59,1513 | 33,5759 | 26,5397 | 42,1941 |
| **7** | Heroini | 52,5045 | 47,5196 | 58,2148 | 48,7782 | 43,0864 | 55,116 |
| **8** | (Heroini, Cichlasomatini) | 60,6922 | 54,1887 | 67,4089 | 59,0664 | 50,1625 | 69,0007 |
| **9** | Pseudocrenilabrinae | 61,6035 | 51,0282 | 72,2752 | 65,937 | 50,2488 | 82,6537 |
| **10** | Haplotilapiines (sensu Schliewen & Stiassny, 2003) | 46,931 | 38,4571 | 56,1764 | 51,7218 | 38,8007 | 65,577 |
| **11** | (Coptodonini, (Pelmatolapiini, (Oreochromini, Austrotilapiines))) | 36,2591 | 30,55 | 42,3341 | - | - | - |
| **12** | Oreochromini | 23,0597 | 17,3184 | 29,4375 | - | - | - |
| **13** | Oreochromini (excluding *Danakilia*) | 20,0208 | 14,9102 | 25,8087 | 18,0508 | 10,0596 | 27,2606 |
| **14** | Australotilapiines (sensu Schwarzer et al., 2009) | 32,336 | 27,4251 | 37,2554 | 38,5032 | 29,3434 | 48,9465 |
| **15** | Steatocranini | 12,3606 | 8,189 | 17,0323 | 7,2786 | 1,5968 | 14,2828 |
| **16** | (Steatocranini, East African Radiation) | 30,9618 | 26,2121 | 35,7999 | - | - | - |
| **17** | East African Radiation (EAR) | 29,0148 | 24,6139 | 33,554 | 31,2377 | 23,6361 | 39,5027 |
| **18** | Trematocarini | 16,2787 | 12,134 | 20,886 | - | - | - |
| **19** | (Bathybatini, Trematocarini) | 24,5124 | 20,1422 | 28,7381 | 18,4013 | 8,9423 | 28,0521 |
| **20** | Bathybatini (including *Hemibates*) | 20,8293 | 16,3384 | 25,4008 | - | - | - |
| **21** | *Bathybates* | 13,3824 | 9,5711 | 17,4699 | - | - | - |
| **22** | *Bathybates* (excluding *Bathybates minor*) | 8,321 | 5,7296 | 10,9658 | 3,5199 | 0,0508 | 8,9029 |
| **23** | East African Radiation (EAR) excluding Boulengerochromini | 28,0044 | 23,8142 | 32,476 | - | - | - |
| **24** | MVhL-clade (sensu Takahashi et al., 2001) | 23,7586 | 20,2542 | 27,4303 | 28,665 | 21,4606 | 36,0061 |
| **25** | Lamprologini | 15,404 | 12,2363 | 18,7212 | 16,1592 | 10,8644 | 22,0851 |
| **26** | 'Lacustrine lamprologines' | 14,4004 | 11,2965 | 17,4509 | - | - | - |
| **27** | 'Non-ossfied lacustrine Lamprologines' | 12,6267 | 9,7538 | 15,5898 | - | - | - |
| **28** | 'Ossified lamprologines' | 10,7055 | 7,5656 | 14,2421 | - | - | - |
| **29** | 'Lower Congo *Lamprologus* clade' | 6,5814 | 4,3037 | 8,8893 | - | - | - |
| **30** | (*Telmatochromis* sp. 'Lufubu', *Neolamprologus brichardi*) | 4,3966 | 2,5273 | 6,5373 | - | - | - |
| **31** | (*Lamprologus werneri*, (*Telmatochromis* cf. *temporalis*, *Lamprologus symoensi*)) | 5,155 | 3,3659 | 7,0883 | - | - | - |
| **32** | (*Telmatochromis* cf. *temporalis*, *Lamprologus symoensi*) | 2,5762 | 1,3417 | 3,8915 | - | - | - |
| **33** | Eretmodini | 7,5933 | 3,9609 | 11,7869 | 0,9978 | 0 | 3,2251 |
| **34** | C-lineage (sensu Clabaut et al., 2005), Eretmodini | 22,9379 | 19,6416 | 26,5627 | - | - | - |
| **35** | Cyphotilapini | 14,2163 | 8,8533 | 19,2146 | - | - | - |
| **36** | Limnochromini | 10,8778 | 5,4295 | 16,1437 | - | - | - |
| **37** | Perisodini | 6,2121 | 3,1807 | 9,5906 | 12,6215 | 5,5788 | 20,0182 |
| **38** | Cyprichromini | 10,5991 | 6,4301 | 15,1157 | 8,7073 | 3,3387 | 14,5712 |
| **39** | 'benthopelagic LT clade' | 16,2835 | 12,927 | 19,6557 | - | - | - |
| **40** | Ectodini | 14,0656 | 11,841 | 17,1722 | 16,5413 | 10,7887 | 22,2136 |
| **41** | 'Malagarasi-*Orthochromis*' | 11,6191 | 8,3234 | 15,0425 | - | - | - |
| **42** | ('Malagarasi-*Orthochromis*', Haplochromini) | 18,5909 | 15,8998 | 21,4448 | - | - | - |
| **43** | Haplochromini | 16,7709 | 14,2491 | 19,3877 | - | - | - |
| **44** | Haplochromini (excluding *Ctenochromis pectoralis*) | 15,6043 | 13,1944 | 18,641 | - | - | - |
| **45** | ('Congo-basin-Haplochromini', ('serranochromines-mt-lineage', 'LML-*Orthochromis*')) | 11,886 | 11,0197 | 15,9706 | - | - | - |
| **46** | ('serranochromines-mt-lineage sensu stricto', *Chetia welwitschi)* | 9,3494 | 7,0896 | 11,5855 | - | - | - |
| **47** | 'serrranochromines-mt-lineage sensu stricto' | 5,5091 | 3,8623 | 7,3446 | - | - | - |
| **48** | (*Serranochromis altus*, *Pharyngochromis* sp. "black bars") | 4,4161 | 2,6918 | 6,24 | - | - | - |
| **49** | ('*Orthochromis*' sp. 'Lufubu ', ('*Pseudocrenilabrus*-group', 'ocellated eggspot Haplochromini') | 15,2646 | 12,896 | 17,6896 | - | - | - |
| **50** | *Pseudocrenilabrus*-group' | 11,886 | 9,5355 | 14,2871 | - | - | - |
| **51** | (*Pseudocrenilabrus multicolor*, *Pseudocrenilabrus nicholsi*) | 2,2222 | 1,1233 | 3,4042 | - | - | - |
| **52** | 'ocellated eggspot Haplochromini' | 14,2479 | 11,9742 | 16,6437 | - | - | - |
| **53** | 'ocellated eggspot Haplochromini' excluding *Astatoreochromis* | 11,9257 | 9,8099 | 13,9919 | - | - | - |
| **54** | (Tropheini, '*Haplochromis*' *vanheusdeni*) | 10,6304 | 8,5408 | 12,8588 | - | - | - |
| **55** | (*Tropheus moorii*, *Tropheus polli*) | 3,5457 | 1,6615 | 5,7965 | - | - | - |
| **56** | Tropheini (excluding Tropheus moorii, Tropheus polli) | 7,2854 | 5,4745 | 9,1975 | - | - | - |
| **57** | Tropheini | 8,7861 | 6,7883 | 10,8889 | 10,4482 | 6,0452 | 15,2956 |
| **58** | (*Haplochromis demeusii*, *Haplochromis fasciatus*) | 5,3419 | 3,2791 | 7,5023 | - | - | - |
| **59** | 'riverine & modern Haplochromini' | 9,5655 | 7,6801 | 11,4783 | - | - | - |
| **60** | (LVRS, *Haplochromis stappersii*) | 0,9936 | 0,5349 | 1,5504 | - | - | - |
| **61** | Lake Victoria Region Superflock (LVRS) | 0,3119 | 0,1262 | 0,5448 | 1,1873 | 0,0323 | 2,9057 |
| **62** | ('riverine & modern Haplochromini', Lake Malawi species flock) | 10,1217 | 8,2901 | 12,1586 | - | - | - |
| **63** | Lake Malawi species flock | 4,0412 | 2,9992 | 5,2618 | 7,7811 | 4,1755 | 11,7381 |
| **64** | 'primarily Mbuna taxa' | 0,6033 | 0,4067 | 0,8207 | - | - | - |
| **65** | 'primarily LM Sanddweller' | 0,6955 | 0,4459 | 0,9647 | - | - | - |
|  |  |  |  |  |  |  |  |
