# Supplementary material for: East African cichlid lineages (Teleostei: Cichlidae) might be older than their ancient host lakes: new divergence estimates for the east African cichlid radiation
Source: BMC Evol Biol. 2019 Apr 25;19:94. doi: 10.1186/s12862-019-1417-0 (PMC6482553; doi:10.1186/s12862-019-1417-0)
Supplement: Supplementary file 3 — Justification for exclusion of several cichlid fossils from calibration. (DOCX 24 kb) [file 12862_2019_1417_MOESM3_ESM.docx]

**Appendix A. Supplementary material**

Appendix S1: Justification for exclusion of several fossils from calibration

Several fossils, which had previously been used in cichlid molecular clock analyses, were excluded from the analysis and have not been used as calibration points due to their questionable or uncertain phylogenetic positions and/or because of lack of mtDNA mitogenome sequences in our dataset:

- *cf*. *Pelmatochromis* spp. from the lower Miocene in Uganda [1]. Reason for exclusion: disarticulated fossil, hence no meristic counts available. Presence of six tubules on the lacrimal contrast with all extant pelmatochromines or chromidotilapiines, which strongly suggests a wrong generic placement of this fossil by Van Couvering (meanwhile three of the four compared *Pelmatochromis* species in the original study have been reassigned to several chromidotilapiine genera).
- ?*Tylochromis* from the Jebel Qatrani Formation in Egypt. Only known from a single lower pharyngeal jaw with hypertrophied median teeth [2, 3]. Therefore, no information on skeleton morphology or meristics is available, which is crucial for unambiguous phylogenetic placement. The same is true for the recently discovered fossil assigned to ?*Tylochromis* from Dur At-Talah (middle Eocene), Libya [4].
- *Oreochromis* †*martyni* was described first as *Sarotherodon* †*martyni* from the Ngorora Formation in Kenya which was assigned to upper Miocene 9.3-12 Ma based on K/AR dates [1, 5]. Holotype and reference specimens are only partially complete, therefore we follow here a more conservative approach not including it our analysis as a robust phylogenetic position is not available for this taxon.

In addition we excluded several fossil taxa as calibration points, because corresponding lineages were not represented in our mitogenome dataset:

- †*Proterocara argentina* was described along with †*Gymnogeophagus eocenicus* [6] and †*Plesioheros chaulidus* [7] from the Lumbrera formation in Northwestern Argentina. It was first suggested to be a member of a clade including the Geophagini, Cichlasomatini and Chaetobranchini [8], but later its affinities were revised and it was inferred to be related with *Crenicichla* and *Teleocichla* [9]. As our taxon sampling is lacking *Crenicichla* and *Teleocichla* we did not include †*Proterocara argentina* in our analysis.
- *Nandopsis* †*woodring*i from Hispaniola was first described as '*Cichlasoma' woodringi* and later transferred to *Nandopsis* [10]. Its age is estimated based on co-distributed plant fossils to middle or late Miocene (5.33 – 15.97 Ma). As our taxon sampling does not include the genus *Nandopsis* it was excluded from our analysis.
- Three fossil specimens LNK.tel7, LNK.Gi2, and LNK.Gi6 from the Baid formation (Ad Darb locality, Tihamat Asir, SW Saudi Arabia) were suggested to be related with ?*Heterochromis* based on a predorsal count higher than one and several squamation characters (e.g. oval form and strongly ctenoid). A robust phylogenetic placement is however still missing [11]. Lippitsch and Micklich [11] suggested an Oligocene age for the Baid formation whereas another study assigned the formation to Early Miocene based on K-Ar ages [12]. As a robust phylogenetic placement is lacking, as the age of the Baid formation is only tentatively known and because our data set is lacking *Heterochromis* we did not use it for calibration.

**References**

1. Van Couvering JAH: **Fossil cichlid fish of Africa**. *Special Pap Paleontol.* 1982, **29**:1–103.

2. Murray AM: **Lower pharyngeal jaw of a cichlid fissh (Actinopterygii; Labroidei) from an Early Oligocene site in the Fayum, Egypt**. *J Vert Paleontol*. 2002, **22**(2):453–455.

3. Murray AM: **Late Eocene and early Oligocene teleost and associated ichthyofauna of the Jebel Qatrani Formation, Fayum, Egypt**. *Palaeontol*. 2004, **47**:711–724.

4. Otero O, Pinton A, Cappetta H, Adnet S, Valentin X, Salem M, Jaeger JJ: **A Fish Assemblage from the Middle Eocene from Libya (Dur At-Talah) and the Earliest Record of Modern African Fish Genera**. *PLoS One* 2015, **10**(12):e0144358.

5. Murray AM, Stewart KM: **A new species of tilapiine cichlid from the Pliocene, Middle Awash, Ethiopia**. *J Vert Paleontol.* 1999, **19**:293–301.

6. Malabarba MC, Malabarba LR, del Papa C: **Gymnogeophagus eocenicus (Perciformes: Cichlidae), an Eocene cichlid from the Lumbrera Formation in Argentina**. *J Vert Paleontol*. 2010, **30**(2):341–350.

7. Perez PA, Malabarba MC, del Papa C: **A new genus and species of Heroini (Perciformes : Cichlidae) from the early Eocene of southern South America**. *Neotrop Ichthyol*. 2010, **8**(3):631–642.

8. Malabarba MC, Zuleta OD, del Papa C: **Proterocara argentina, a new fossil cichlid from the Lumbrera Formation, Eocene of Argentina**. *J Vert Paleontol*. 2006, **26**(2):267–275.

9. Smith WL, Chakrabarty P, Sparks JS: **Phylogeny, taxonomy, and evolution of Neotropical cichlids (Teleostei: Cichlidae: Cichlinae)**. *Cladistics* 2008, **24**:625–641.

10. Chakrabarty P: **Taxonomic status of the Hispaniolan Cichlidae**. *Occasional pap Mus Zool University Michigan* 2006, **737**:1–17.

11. Lippitsch E, Micklich N: **Cichlid fish biodiversity in an Oligocene lake**. *Ital J Zool*. 1998, **65**:185–188.

12. Brown GF, Schmidt DL, Huffman AC: **Geology of the Arabian Peninsula; shield area of western Saudi Arabia**. *US Geol Surv Prof Pap.* 1998, **560-A**.
