# Supplementary material for: East African cichlid lineages (Teleostei: Cichlidae) might be older than their ancient host lakes: new divergence estimates for the east African cichlid radiation
Source: BMC Evol Biol. 2019 Apr 25;19:94. doi: 10.1186/s12862-019-1417-0 (PMC6482553; doi:10.1186/s12862-019-1417-0)
Supplement: Supplementary file 4 — Table S2. Comprehensive list of mean divergence ages and their corresponding 95% HPD age ranges of selected nodes. (Node numbers 1 to 65 correspond to numbers depicted in Fig. 2). (DOCX 32 kb) [file 12862_2019_1417_MOESM4_ESM.docx]

**SchedelEtal_Table_A3**

Table A.3

Overview of the taxon sampling for the nuclear markers (RAG1, ENC1, Rh1 and ttna TMO) with corresponding Genbank accession numbers.

Table A.3: Overiew of the taxon sampling for the nuclear markers with corresponding Genbank accession numbers.

| **Subfamily** | **Tribe/Group names used in this study** | **Genus** | **Species** | **Autor** | **RAG1** | **ENC1** | **Rh1** | **ttna TMO** | **Comment: deposited in GenBank as** |
| --- | --- | --- | --- | --- | --- | --- | --- | --- | --- |
| Etroplinae | Etroplinae | *Etroplus* | *maculatus* | (Bloch 1795) | EF095672 | KX347684 | - | AY662818 |  |
| Etroplinae | Etroplinae | *Paretroplus* | *maculatus* | Kiener & Maugé 1966 | JX189870 | - | - | AY662824 |  |
| Ptychochrominae | Paratilapiinae | *Paratilapia* | *polleni* | Bleeker 1868 | JX189869 | JX188938 | - | DQ119251 |  |
| Cichlinae | Retroculini | *Retroculus* | *xinguensis* | Gosse 1971 | KF557159 | MF175577 | MF078373 | AY662857 |  |
| Cichlinae | Astronotini | *Astronotus* | *ocellatus* | (Agassiz 1831) | EF095671 | - | - | AY662851 |  |
| Cichlinae | Chaetobranchini | *Chaetobranchopsis* | *orbicularis* | (Steindachner 1875) | - | MF175571 | MF078368 | AY662852 |  |
| Cichlinae | Cichlasomatini | *Aequidens* | *diadema* | (Heckel 1840) | EU706374 | MF175531 | MF078377 | - |  |
| Cichlinae | Cichlasomatini | *Aequidens* | *metae ZM-2008* | Eigenmann 1922 | EU706376 | KX347666 | - | - |  |
| Cichlinae | Cichlasomatini | *Aequidens* | *tetramerus* | (Heckel, 1840) | EU706386 | MF175530 | MF078378 | - |  |
| Cichlinae | Cichlasomatini | *Andinoacara* | *rivulatus* | (Günther 1860) | KF557140 | MF175523 | MF078379 | - |  |
| Cichlinae | Cichlasomatini | *Bujurquina* | *apoparuana* | Kullander 1986 | - | MF175520 | MF078380 | - |  |
| Cichlinae | Cichlasomatini | *Cichlasoma* | *dimerus* | (Heckel 1840) | EU706366 | MF175529 | MF078428 | - |  |
| Cichlinae | Cichlasomatini | *Krobia* | *xinguensis* | Kullander 2012 | - | MF175534 | MF078385 | - |  |
| Cichlinae | Cichlasomatini | *Laetacara* | *dorsigera* | (Heckel 1840) | EU706387 | MF175524 | MF078386 | - |  |
| Cichlinae | Cichlasomatini | *Laetacara* | *thayeri* | (Steindachner 1875) | EU706401 | MF175525 | MF078387 | - |  |
| Cichlinae | Cichlasomatini | *Nannacara* | *anomala* | Regan 1905 | EU706391 | MF175535 | MF078388 | - |  |
| Cichlinae | Cichlasomatini | *Nannacara* | *taenia* | Regan 1912 | EU706393 | MF175536 | MF078390 | - |  |
| Cichlinae | Heroini | *Herichthys* | *cyanoguttatus* | Baird & Girard 1854 | JX213234 | MF175492 | MF078367 | - |  |
| Cichlinae | Heroini | *Hypselecara* | *temporalis* | (Günther 1862) | KF557148 | KF556772 | - | DQ119248 |  |
| Cichlinae | Heroini | *Parachromis* | *dovii* | (Günther 1864) | GU595414 | MF175481 | MF078451 | DQ119233 |  |
| Cichlinae | Heroini | *Parachromis* | *managuensis* | (Günther 1867) | GU595411 | KF556781 | KP715365 | - |  |
| Cichlinae | Heroini | *Petenia* | *splendida* | Günther 1862 | GU595412 | MF175486 | MF078454 | - |  |
| Cichlinae | Heroini | *Pterophyllum* | *leopoldi* | (Gosse, 1963) | KF557158 | MF175517 | MF078456 | - |  |
| Cichlinae | Heroini | *Rocio* | *octofasciata* | (Regan 1903) | GU595401 | MF175506 | MF078426 | AF113072 |  |
| Cichlinae | Heroini | *Symphysodon* | *discus* | Heckel 1840 | EU497298 | MF175509 | MF078458 | - |  |
| Cichlinae | Heroini | *Thorichthys* | *meeki* | Brind, 1918 | EF362590 | MF175494 | MF078462 | AY279772 |  |
| Cichlinae | Heroini | *Uaru* | *amphiacanthoides* | Heckel 1840 | KF557164 | MF175510 | MF078464 | AF113068 |  |
| Cichlinae | Heroini | *Amphilophus* | *citrinellus* | (Günther 1864) | GU595398 | MF175476 | MF078423 | DQ119227 |  |
| Cichlinae | Heroini | *Astatheros* | *macracanthus* | (Günther 1864) | EF362591 | MF175505 | MF078425 | - | *Amphilophus macracanthus* |
| Cichlinae | Heroini | *Herichthys* | *carpintis* | (Jordan & Snyder 1899) | EF362585 | JX213221 | - | DQ119230 |  |
| Cichlinae | Heroini | *Vieja* | *maculicauda* | (Regan, 1905) | EF362592 | MF175489 | MF078452 | - | *Paraneetroplus maculicauda* |
| Cichlinae | Cichlini | *Cichla* | *temensis* | Humboldt 1821 | - | MF175574 | MF078371 | AY662853 |  |
| Cichlinae | Geophagini | *Geophagus* | *brasiliensis* | (Quoy & Gaimard 1824) | EU706360 | - | - | EU888082 |  |
| Cichlinae | Geophagini | *Geophagus* | *steindachneri* | Eigenmann & Hildebrand 1922 | - | MF175565 | MF078407 | - |  |
| Cichlinae | Geophagini | *Gymnogeophagus* | *balzanii* | (Perugia, 1891) | - | MF175564 | - | - |  |
| Cichlinae | Geophagini | *Gymnogeophagus* | *setequedas* | Reis, Malabarba & Pavanelli 1992 | - | MF175563 | - | - |  |
| Cichlinae | Geophagini | *Mikrogeophagus* | *ramirezi* | (Myers & Harry 1948) | KF557154 | MF175567 | MF078416 | - |  |
| Pseudocrenilabrinae | Tylochromini | *Tylochromis* | *polylepis* | (Boulenger 1900) | DQ012231 | KX347703 | KP130828 | KP130700 |  |
| Pseudocrenilabrinae | Hemichromini | *Hemichromis* | *elongatus* | (Guichenot 1861) | KF557109 | GQ168315 | - | GQ168189 |  |
| Pseudocrenilabrinae | Hemichromini | *Hemichromis* | *letourneuxi* | Sauvage, 1880 | KF557110 | KX347688 | - | - |  |
| Pseudocrenilabrinae | Chromidotilapini | *Benitochromis* | *batesii* | (Boulenger 1901) | KF557095 | KF556715 | - | - |  |
| Pseudocrenilabrinae | Chromidotilapini | *Nanochromis* | *parilus* | Roberts & Stewart 1976 | KF557116 | KF556736 | - | GQ168191 |  |
| Pseudocrenilabrinae | Etiini | *Etia* | *nguti* | Schliewen & Stiassny 2003 | - | GQ168280 | - | AY662862 |  |
| Pseudocrenilabrinae | Pelmatolapiini | *Pelmatolapia* | *mariae* | (Boulenger, 1899) | KF557134 | KF556755 | - | JX910764 |  |
| Pseudocrenilabrinae | Gobiocichlini | *Tilapia* | *brevimanus* | Boulenger, 1911 | - | GQ168331 | - | GQ168205 |  |
| Pseudocrenilabrinae | Coptodonini | *Coptodon* | *discolor* | (Günther 1903) | - | GQ168304 | - | GQ168178 |  |
| Pseudocrenilabrinae | Coptodonini | *Coptodon* | *zillii* | (Gervais 1848) | - | GQ168339 | - | GQ168213 |  |
| Pseudocrenilabrinae | Pelmatochromini | *Pelmatochromis* | *buettikoferi* | (Pellegrin, 1900) | - | GQ168286 | - | GQ168160 |  |
| Pseudocrenilabrinae | Oreochromini | *Konia* | *eisentrauti* | (Trewavas 1962) | KF557111 | KF556731 | - | JX910783 |  |
| Pseudocrenilabrinae | Oreochromini | *Myaka* | *myaka* | Trewavas 1972 | KF557115 | KF556735 | - | - |  |
| Pseudocrenilabrinae | Oreochromini | *Oreochromis* | *niloticus* | (Linnaeus 1758) | AB915550 | GQ168283 | AY775108 | GQ168157 |  |
| Pseudocrenilabrinae | Oreochromini | *Oreochromis* | *tanganicae* | (Günther 1894) | KF557119 | KF556739 | KP130815 | KP130687 |  |
| Pseudocrenilabrinae | Oreochromini | *Pungu* | *maclareni* | (Trewavas, 1962) | KF557126 | KF556746 | - | - |  |
| Pseudocrenilabrinae | Oreochromini | *Sarotherodon* | *galilaeus* | (Linnaeus, 1758) | KF557128 | KF556748 | - | JX910757 |  |
| Pseudocrenilabrinae | Oreochromini | *Sarotherodon* | *melanotheron* | Rüppell 1852 | - | GQ168290 | AB084940 | GQ168164 |  |
| Pseudocrenilabrinae | Oreochromini | *Stomatepia* | *pindu* | Trewavas 1972 | KF557132 | KF556753 | - | - |  |
| Pseudocrenilabrinae | Oreochromini | *Tristramella* | *simonis* | (Günther 1864) | - | GQ168316 | - | GQ168190 |  |
| Pseudocrenilabrinae | Tilapiini | *Congolapia* | *bilineata* | (Pellegrin 1900) | 'Lefini' | GQ168278 | 'Lefini' | GQ168152 |  |
| Pseudocrenilabrinae | Tilapiini | *Tilapia* | *ruweti* | (Poll & Thys van den Audenaerde 1965) | KF557135 | KF556756 | - | JX910751 |  |
| Pseudocrenilabrinae | Tilapiini | *Tilapia* | *sparrmanii* | Smith 1840 | KF557136 | KF556757 | KP130844 | KP130716 |  |
| Pseudocrenilabrinae | Steatocranini | *Steatocranus* | *tinanti* | (Poll 1939) | KF557130 | KF556751 | - | AY662873 |  |
| Pseudocrenilabrinae | Steatocranini | *Steatocranus* | *ubanguiensis* | Roberts & Stewart 1976 | KF557131 | KF556752 | - | GQ168203 |  |
| Pseudocrenilabrinae | Boulengerochromis / 'most ancient Tanganyika tribes' | *Boulengerochromis* | *microlepis* | (Boulenger 1899) | KF557097 | KF556717 | AB084928 | KP130686 |  |
| Pseudocrenilabrinae | Bathybatini / 'most ancient Tanganyika tribes' | *Bathybates* | *fasciatus* | Boulenger 1901 | KF557094 | KF556714 | - | - |  |
| Pseudocrenilabrinae | Bathybatini / 'most ancient Tanganyika tribes' | *Bathybates* | *graueri* | Steindacher, 1911 | KP131318 | KX347672 | KP130817 | KP130689 |  |
| Pseudocrenilabrinae | Trematocarini / 'most ancient Tanganyika tribes' | *Trematocara* | *nigrifrons* | Boulenger, 1906 | KP131317 | KP131198 | KP130816 | KP130688 |  |
| Pseudocrenilabrinae | Lamprologini | *Altolamprologus* | *calvus* | (Poll 1978) | KF557091 | KF556711 | AB084924 | - |  |
| Pseudocrenilabrinae | Lamprologini | *Julidochromis* | *ornatus* | Boulenger 1898 | DQ012237 | KP131216 | KP130834 | KP130706 |  |
| Pseudocrenilabrinae | Lamprologini | *Lamprologus* | *callipterus* | Boulenger 1906 | FJ706526 | KX347689 | KP130808 | KP130680 |  |
| Pseudocrenilabrinae | Lamprologini | *Lepidiolamprologus* | *elongatus* | (Boulenger 1898) | FJ706502 | KP131191 | KP130809 | KP130681 |  |
| Pseudocrenilabrinae | Lamprologini | *Neolamprologus* | *caudopunctatus* | (Poll 1978) | KP131308 | KP131189 | KP130807 | KP130679 |  |
| Pseudocrenilabrinae | Lamprologini | *Neolamprologus* | *pulcher* | (Trewavas & Poll 1952) | KP131313 | KX347691 | KP130812 | KP130684 |  |
| Pseudocrenilabrinae | Lamprologini | *Neolamprologus* | *tetracanthus* | (Boulenger 1899) | FJ706485 | KP131193 | KP130811 | KP130683 |  |
| Pseudocrenilabrinae | Lamprologini | *Telmatochromis* | *dhonti* | (Boulenger 1919) | KX327255 | KX336091 | - | KX329277 |  |
| Pseudocrenilabrinae | Lamprologini | *Variabilichromis* | *moorii* | (Boulenger 1898) | FJ706503 | GQ168336 | KP130813 | GQ168187 |  |
| Pseudocrenilabrinae | Eretmodini | *Eretmodus* | *cyanostictus* | Boulenger 1898 | KF557103 | KF556724 | KP130818 | KP130690 |  |
| Pseudocrenilabrinae | Eretmodini | *Spathodus* | *marlieri* | Poll 1950 | KF557129 | KF556750 | - | - |  |
| Pseudocrenilabrinae | Cyphotilapiini / 'ancient Tanganyika mouthbrooders' | *Cyphotilapia* | *frontosa* | (Boulenger 1906) | DQ012219 | KX347683 | AB084929 | KX328641 |  |
| Pseudocrenilabrinae | Cyphotilapiini / 'ancient Tanganyika mouthbrooders' | *Trematochromis* | *benthicola* | (Matthes 1962) | KP131320 | KP131202 | KP130820 | KP130692 |  |
| Pseudocrenilabrinae | Limnochromini / 'ancient Tanganyika mouthbrooders' | *Gnathochromis* | *permaxillaris* | (David 1936) | KM263626 | KP131211 | AB084932 | KP130701 |  |
| Pseudocrenilabrinae | Limnochromini / 'ancient Tanganyika mouthbrooders' | *Greenwoodochromis* | *abeelei* | (Poll 1949) | KM263625 | KX347690 | KP130827 | KP130699 | *Limnochromis abeelei* |
| Pseudocrenilabrinae | Cyprichromini / 'ancient Tanganyika mouthbrooders' | *Cyprichromis* | *leptosoma* | (Boulenger 1898) | DQ012234 | KF556722 | AB084930 | KP130678 |  |
| Pseudocrenilabrinae | Cyprichromini / 'ancient Tanganyika mouthbrooders' | *Paracyprichromis* | *brieni* | (Poll 1981) | KF557121 | KF556741 | - | - |  |
| Pseudocrenilabrinae | Perissodini / 'ancient Tanganyika mouthbrooders' | *Haplotaxodon* | *microlepis* | Boulenger 1898 | KF557107 | KF556729 | KP130830 | KP130702 |  |
| Pseudocrenilabrinae | Perissodini / 'ancient Tanganyika mouthbrooders' | *Perissodus* | *microlepis* | Boulenger 1898 | DQ012244 | KX347694 | KP130831 | KP130703 |  |
| Pseudocrenilabrinae | Benthochromini / 'ancient Tanganyika mouthbrooders' | *Benthochromis* | *tricoti* | (Poll 1948) | KF557096 | KF556716 | AB084927 | - |  |
| Pseudocrenilabrinae | Ectodini / 'ancient Tanganyika mouthbrooders' | *Aulonocranus* | *dewindti* | (Boulenger 1899) | KF557093 | KF556713 | KP130801 | KP130673 |  |
| Pseudocrenilabrinae | Ectodini / 'ancient Tanganyika mouthbrooders' | *Callochromis* | *macrops* | (Boulenger 1898) | KM263620 | KP131184 | KP130802 | KP130674 |  |
| Pseudocrenilabrinae | Ectodini / 'ancient Tanganyika mouthbrooders' | *Grammatotria* | *lemairii* | Boulenger 1899 | DQ012242 | KP131185 | KP130803 | KP130675 |  |
| Pseudocrenilabrinae | Ectodini / 'ancient Tanganyika mouthbrooders' | *Ophthalmotilapia* | *ventralis* | (Boulenger 1898) | KF557118 | KF556738 | AY775109 | KP130676 |  |
| Pseudocrenilabrinae | Ectodini / 'ancient Tanganyika mouthbrooders' | *Xenotilapia* | *spiloptera* | Poll & Stewart 1975 | KX327371 | KP131187 | KP130805 | KP130677 |  |
| Pseudocrenilabrinae | Haplochromini / Tropheini | *Ctenochromis* | *horei* | (Günther 1894) | DQ012250 | KX347681 | KP130822 | KP130694 |  |
| Pseudocrenilabrinae | Haplochromini / Tropheini | *Gnathochromis* | *pfefferi* | Boulenger 1898) | KF557104 | KF556725 | KP130823 | KP130695 |  |
| Pseudocrenilabrinae | Haplochromini / Tropheini | *Limnotilapia* | *dardennii* | (Boulenger 1899) | KF557113 | KF556733 | - | - |  |
| Pseudocrenilabrinae | Haplochromini / Tropheini | *Lobochilotes* | *labiatus* | (Boulenger 1898) | DQ012210 | KP131206 | KP130824 | KP130696 |  |
| Pseudocrenilabrinae | Haplochromini / Tropheini | *Petrochromis* | *polyodon* | Boulenger, 1898 | KF557122 | KF556742 | - | - |  |
| Pseudocrenilabrinae | Haplochromini / Tropheini | *Pseudosimochromis* | *curvifrons* | (Poll, 1942) | KF557125 | KF556745 | - | - |  |
| Pseudocrenilabrinae | Haplochromini / Tropheini | *Pseudotropheus* | *sp. 'acei'* |  | KP131335 | KX347697 | GQ422475 | KP130713 |  |
| Pseudocrenilabrinae | Haplochromini / Tropheini | *Tropheus* | *duboisi* | Marlier 1959 | KF557137 | KF556758 | AY775111 | - |  |
| Pseudocrenilabrinae | Haplochromini / Tropheini | *Tropheus* | *moorii* | Boulenger 1898 | KP131323 | HM050026 | KP130825 | KP130697 |  |
| Pseudocrenilabrinae | Haplochromini / 'serranochromines-mt-lineage' | *Serranochromis* | *macrocephalus* | (Boulenger 1899) | KP131328 | KP131214 | KP130832 | KP130704 |  |
| Pseudocrenilabrinae | Haplochromini / 'Pseudocrenilabrus-group' (Northern-Zambian-Orthochromis) | *Orthochromis* | *luongoensis* | (Greenwood & Kullander 1994) | KF557120 | KF556740 | - | - |  |
| Pseudocrenilabrinae | Haplochromini / '*Pseudocrenilabrus*-group' | New Luongo cichlid |  |  | KF557099 | KF556719 | - | - | *Chetia mola* |
| Pseudocrenilabrinae | Haplochromini / '*Pseudocrenilabrus*-group' | *Pseudocrenilabrus* | *multicolor* | (Schöller 1903) | DQ012215 | KX347695 | - | - |  |
| Pseudocrenilabrinae | Haplochromini / '*Pseudocrenilabrus*-group' | *Pseudocrenilabrus* | *philander* | (Weber 1897) | KM263622 | KX347696 | KP130819 | KP130691 |  |
| Pseudocrenilabrinae | Haplochormini / 'ocellated eggspot Haplochromini' (Lake Malawi species flock) | *Cynotilapia* | *afra* | (Günther 1894) | - | KX347682 | AY775118 | - |  |
| Pseudocrenilabrinae | Haplochormini / 'ocellated eggspot Haplochromini' (Lake Malawi species flock) | *Maylandia* | *pulpican* | (Tawil 2002) | KX327023 | KP131224 | KP130842 | KP130714 | *Cynotilapia pulpican* |
| Pseudocrenilabrinae | Haplochormini / 'ocellated eggspot Haplochromini' (Lake Malawi species flock) | *Dimidiochromis* | *compressiceps* | (Boulenger 1908) | AB915557 | - | AY775059 | - |  |
| Pseudocrenilabrinae | Haplochormini / 'ocellated eggspot Haplochromini' (Lake Malawi species flock) | *Maylandia* | *zebra* | (Boulenger 1899) | DQ012249 | - | AY775114 | AY662872 |  |
| Pseudocrenilabrinae | Haplochormini / 'ocellated eggspot Haplochromini' (Lake Malawi species flock) | *Melanochromis* | *auratus* | Boulenger, 1897 | DQ012220 | - | AY775115 | - |  |
| Pseudocrenilabrinae | Haplochormini / 'ocellated eggspot Haplochromini' (Lake Malawi species flock) | *Rhamphochromis* | *esox* | (Boulenger 1908) | KP131336 | KX347700 | KP130843 | KP130715 |  |
| Pseudocrenilabrinae | Haplochromini / 'ocellated eggspot Haplochromini' (riverine & modern Haplochromini) | *Haplochromis* | *burtoni* | (Günther 1894) | DQ012245 | KF556712 | AB084925 | KP130672 |  |
| Pseudocrenilabrinae | Haplochromini / 'ocellated eggspot Haplochromini' (riverine & modern Haplochromini) | *Haplochromis* | *cf. Stappersii BSM2014* | Poll, 1943 | KP131333 | KP131221 | KP130839 | KP130711 |  |
| Pseudocrenilabrinae | Haplochromini / 'ocellated eggspot Haplochromini' (riverine & modern Haplochromini) | *Haplochromis* | *pyrrhocephalus* | Witte & Witte-Maas 1987 | AB915558 | - | AB667037 | - |  |
| Pseudocrenilabrinae | Haplochromini / 'ocellated eggspot Haplochromini' (riverine & modern Haplochromini) | *Haplochromis* | *sauvagei* | (Pfeffer 1896) | KP131332 | KP131220 | KP130838 | KP130710 |  |
| Pseudocrenilabrinae | Haplochromini / 'ocellated eggspot Haplochromini' (riverine & modern Haplochromini) | *Haplochromis* | *sp. Chipwa* |  | KX326611 | KX335259 | - | KX328453 |  |
| Pseudocrenilabrinae | Haplochromini / 'ocellated eggspot Haplochromini' (riverine & modern Haplochromini) | *Pundamilia* | *nyererei* | (Witte-Maas & Witte 1985) | KM263628 | - | KP130837 | KP130709 |  |
